# Propargylation of CoQ0 through the Redox Chain Reaction

**DOI:** 10.1021/acs.joc.1c02685

**Published:** 2021-12-21

**Authors:** Robert Pawlowski, Maciej Stodulski, Jacek Mlynarski

**Affiliations:** Institute of Organic Chemistry, Polish Academy of Sciences, Kasprzaka 44/52, 01-224 Warsaw, Poland

## Abstract

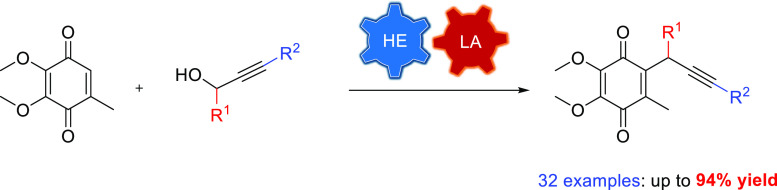

An efficient catalytic propargylation of CoQ0 is described by employing the cooperative effect of Sc(OTf)_3_ and Hantzsch ester. It is suggested to work through the redox chain reaction, which involves hydroquinone and dimeric propargylic moiety intermediates. A broad range of propargylic alcohols can be converted into the appropriate derivatives of CoQ0 containing triple bonds in good to excellent yields. The mechanism of the given transformation is also discussed.

## Introduction

Quinones are an important class of compounds for most living organisms because they participate in the cellular aerobic respiration process (e.g., *ubiquinone*);^1^ serve as electron acceptors in electron transport chains in photosynthesis (e.g., *plastoquinone* and *phylloquinone*); participate in the blood coagulation process, preventing excessive bleeding (vitamin *K*); control binding of calcium in bones; and more.^2,3^ Not surprisingly, many of their synthetic derivatives have been of pharmacological interest and extensively studied as drug candidates in the fight against cancers (e.g., *Daunorubicin*), microorganisms (e.g., *Rhein* and *Mepron*), and more.^4^ The most common and widely used strategy for derivatization of quinones involves a multistep process that required reduction of the corresponding p-quinone and then reoxidation to the corresponding p-quinone.^5−13^ An alternative attempt involves utilization of chloromethylated quinone intermediate and metal-catalyzed cross-coupling reaction with metalorganic reagents.^14−18^ These processes, although effective, are time-consuming and not economically friendly, involve few steps, and generate many byproducts. To overcome some of the abovementioned problems, radical C-H functionalization with boronic acids and other coupling reagents has been elaborated.^19−22^ However, direct functionalization of p-quinones in a one-step process remains challenging. In this context, Li and colleagues described an electrophilic alkylation of p-quinones by various allyl or benzyl acetates through a redox chain reaction.^23,24^ This Lewis acid-catalyzed Friedel–Crafts alkylation process led to the formation of many allyl and benzyl derivatives in reasonable yields. In addition, Lu demonstrated that this transformation can also be achieved in purely organocatalytic fashion, although with very limited scope.^25^ However, propargylation of p-quinones is yet more challenging and remains unknown, which is undesirable while a propargylic motif is common in many natural products, its derivatives, and synthetic intermediates.^26^ Herein, we report the first direct intermolecular propargylation of CoQ0 using various propargylic alcohols by a dual catalysis concept that involves the application of metal triflate and Hantzch ester through the redox chain reaction mechanism.

## Results and Discussion

To develop a practical catalytic system for propargylation of p-quinones, we began our studies by establishing a set of appropriate reagents, catalysts, and reaction conditions. As a model, we chose reaction between p-quinone **1** and propargylic alcohol **2**. Our set of choice was based on studies in the literature that reported that reaction between aromatic derivatives and appropriate propargylic alcohols can proceed easily.^26^ Therefore, we postulated that it should be possible to reduce in situ p-quinone to hydroquinone and combine the reaction of the redox chain according to studies in the literature with our previous findings to enforce propargylation of quinones.^23^ First, we tried to determine the optimal reaction conditions based on previous studies by our group.^27^ We began our course by examining a series of catalytic systems. After many trials, we found that the best results can be achieved by treating compounds **1** and **2** with Sc(OTf)_3_ and Hantzsch ester in dichloromethane (DCM) for 48 h. Under these reaction conditions, we obtained desired product **3**, however, in very poor yield (33%, entry 4, Table 1). Further experiments revealed that both Sc(OTf)_3_ and Hantzsch ester were necessary to promote the propargylation process successfully (Table 1, entry 1). Two catalysts were most effective in this transformation: Sc(OTf)_3_ and InBr_3_ (Table 1, entries 4, 12); however, results were not satisfactory. Encouraged by our findings, different solvents were probed next to examine their impact on the reaction results. Our optimization studies showed that the best results can be achieved using acetonitrile as a solvent. In these conditions, after 48 h at room temperature, the desired product was isolated in 38% yield.

**Table 1:**
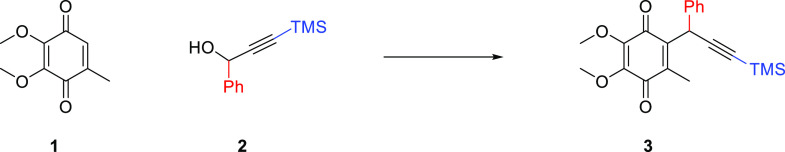
Optimization of the Reaction Conditions for the Direct Propargylation of CoQ0^a^

no	catalyst (10 mol %)	solvent	temperature (°C)	time (h)	yield (%)^b^
1	-	DCM	rt	48	0
2	DPP	DCM	rt	48	0
3	*N*-Tf amide	DCM	rt	48	0
4	Sc(OTf)_3_	DCM	rt	48	33
5	Zn(OTf)_2_	DCM	rt	48	0
6	Er(OTf)_3_	DCM	rt	48	0
7	Y(OTf)_3_	DCM	rt	48	0
8	La(OTf)_3_	DCM	rt	48	0
9	Bi(OTf)_3_	DCM	rt	48	5
10	Cu(OTf)_2_	DCM	rt	48	trace
11	AgOTf	DCM	rt	48	0
12	InCl_3_	DCM	rt	48	30
13	Sc(OTf)_3_	DCM	rt	48	0
14	Sc(OTf)_3_	MeCN	rt	48	38
15	Sc(OTf)_3_ (1 equiv)	DCM	rt	48	15
**16**	**Sc(OTf)_3_**	**MeCN**	**60**	**24**	**86**
17	Sc(OTf)_3_	THF	60	48	0
18	Sc(OTf)_3_	PhMe	60	48	0
19	Sc(OTf)_3_	PhCF_3_	60	48	0
20	Sc(OTf)_3_	DCE	60	48	15

a Unless otherwise indicated, all reactions were performed as follows: reaction scale: 0.15 mmol, 10 mol % catalyst, HE 5 mol %, Ar, 1 mL solvent, temp. 60 °C, reaction time 24 h.

b Isolated yield.

Other polar or nonpolar solvents hampered the reaction or blocked it completely (see Supporting Information for more results). Therefore, to facilitate the product **3** formation, the impact of the temperature on the reaction result was examined next. It turned out that the running reaction in acetonitrile at 60 °C led to the formation of p-quinone **3** in an 86% yield. The reaction conditions allowed us to significantly reduce the reaction time to 24 h (Table 1, entry 16).

**Scheme 1:**
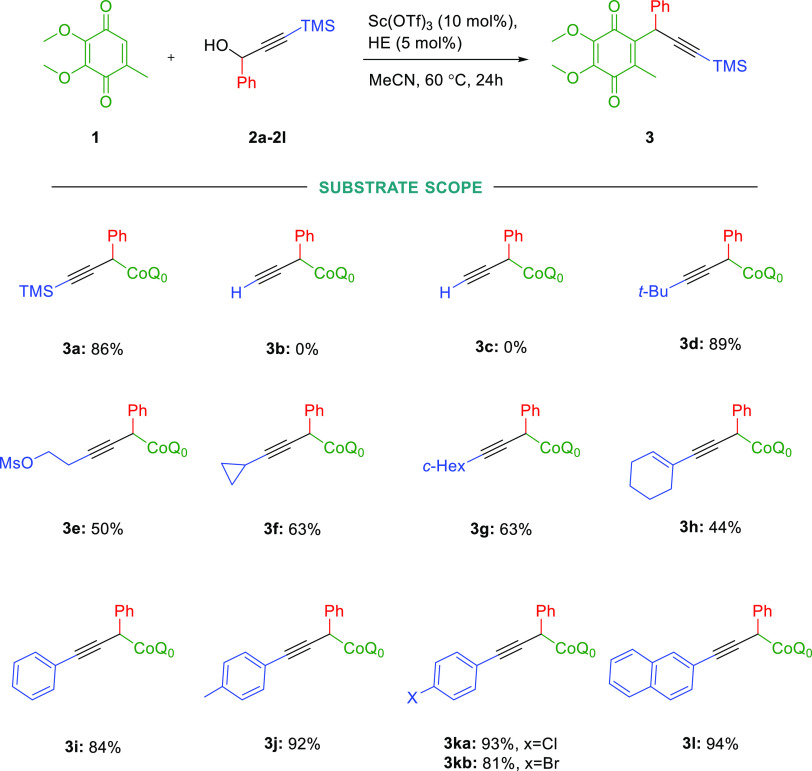
Direct Propargylation of CoQ0 by Various Propargyl Derivatives Unless otherwise indicated, all reactions were performed as follows: reaction scale: 0.15 mmol, 10 mol % of catalyst, HE 5 mol %, Ar, MeCN 1 mL, 60 °C, reaction time 24 h.

Therefore, after a screening of dozens of reaction conditions, the optimal conditions for direct propargylation of p-quinone were identified as Sc(OTf)_3_ (10 mol %) and Hantzsch ester (5 mol %) in acetonitrile at 60 °C and the reaction time of 24 h. With the optimized reaction condition in hand, we surveyed the reaction scope using a series of propargyl derivatives. First, we focused our attention on examining the variation of the terminal substituent of the triple bond (Scheme 1). The examined process generally occurred in good to very good yields (up to 94%); however, to our surprise, derivative **2b** gave no product at all, which might indicate that different activation mechanisms of propargylic alcohols were involved. We observed a similar result when acetylated reagent **2c** was taken. However, alkyl (**2d**–**2e**), cycloalkyl, (**2e**–**2h**), and various aryl substituted groups (**2h**–**2L**) were well tolerated.

**Scheme 2:**
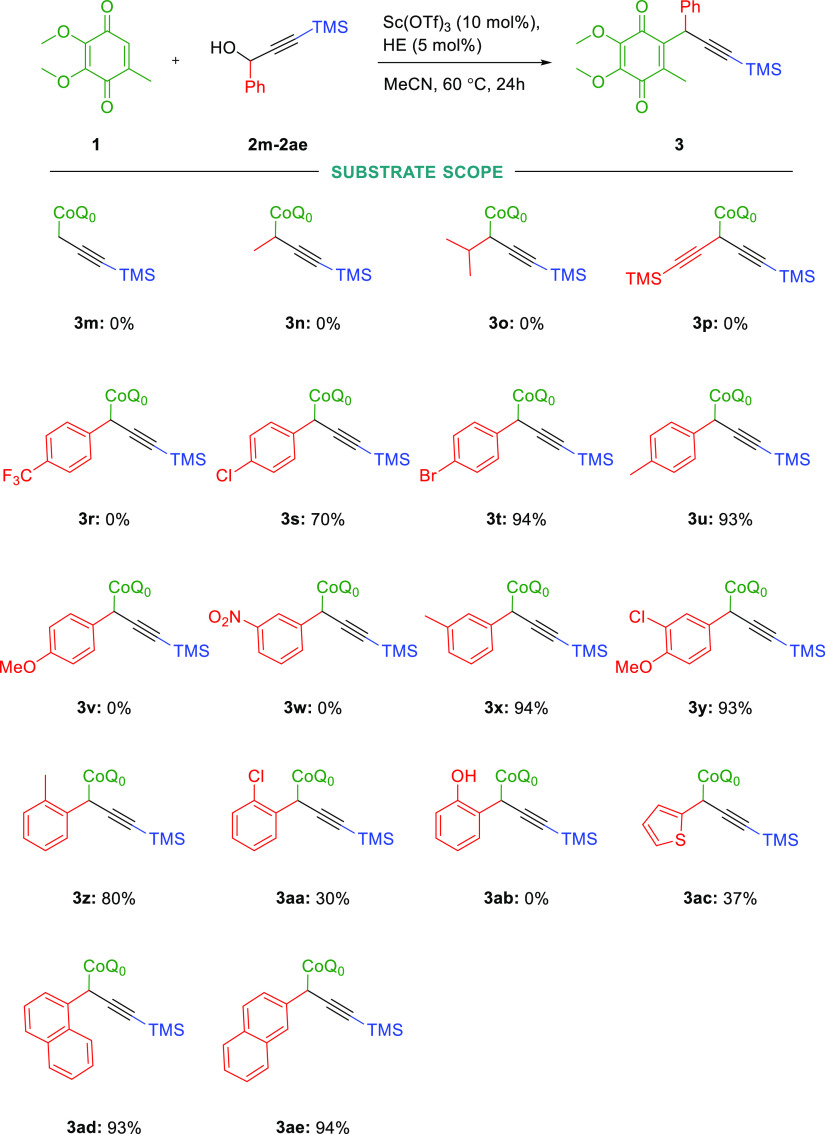
Direct Propargylation of CoQ0 by Various Propargyl Derivatives Unless otherwise indicated, all reactions were performed as follows: reaction scale: 0.15 mmol, 10 mol % of catalyst, HE 5 mol %, Ar, MeCN 1 mL, 60 °C, reaction time 24 h.

In order to show a broader application of the examined transformation, we turned our attention into examining the substituents next to the hydroxyl group (Scheme 2). As expected, derivate **2m** without the phenyl group and derivatives containing alkyl groups (**2n**–**2p**) did not give any product at all.

This indicated that the reaction takes place through the carbocationic intermediate, and the aryl group is necessary to stabilize it. Therefore, we focused our attention on testing variation of the aromatic functionality of derivatives **2r**–**2ae**. The phenyl group or naphtyl that was not substituted with this protocol (**2ad** and **2ae**) gave very good results (up to 94% yield). Variation of the aromatic functionality showed that weak electron-donating groups like methyl (**2u**, **2x**, and **2z**) led to the corresponding products in very good yields (up to 93%). In particular, propargyl alcohols containing halogen substituents (**2s**, **2t**, **2y**, and **2aa**) were also accepted in this transformation, leading to the corresponding products in reasonable to excellent yields (30–94%). Substituents in the o- and m-positions were also accepted in this transformation. However, strongly electron-donating groups containing oxygen atoms (**2v** and **2ab**) gave no product at all. The same result has been observed for strongly electron-withdrawn groups, such as CF_3_ (**2r**) and NO_2_ (**2w**). These observations gave us a hint that reaction might occur via *a* dimeric form of propargylic alcohol, and its formation depends on the electronic nature of the reagent.

**Scheme 3:**
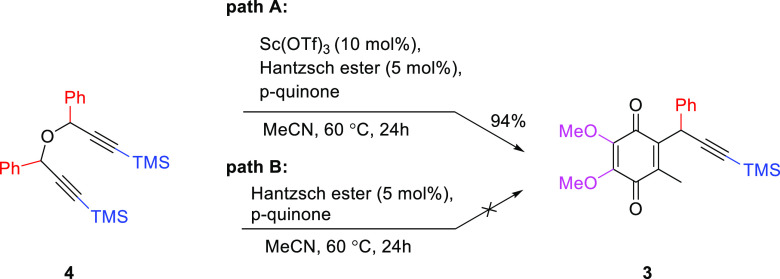
Control Experiments for the Propargylation of CoQ0 Unless otherwise indicated, all reactions were performed as follows: reaction scale: 0.15 mmol, 10 mol % of catalyst, HE 5 mol %, Ar, MeCN 1 mL, temp. 60 °C, reaction time 24 h; isolated yields.

To obtain more information about the possible mechanistic path of the described transformation, several additional experiments were carried out (Scheme 3). First, the reaction between the dimeric form of propargyl alcohol **4** and p-quinone **1** was studied under standard reaction conditions (path A). In this example, the formation of the desired product was observed in almost quantitative yield, which might indicate that dimer **4** is reversibly converted to propargyl carbocation in the presence of Lewis acid. To prove that, a second experiment was carried out under the same reaction conditions, but without the addition of Sc(OTf)_3_.

**Scheme 4:**
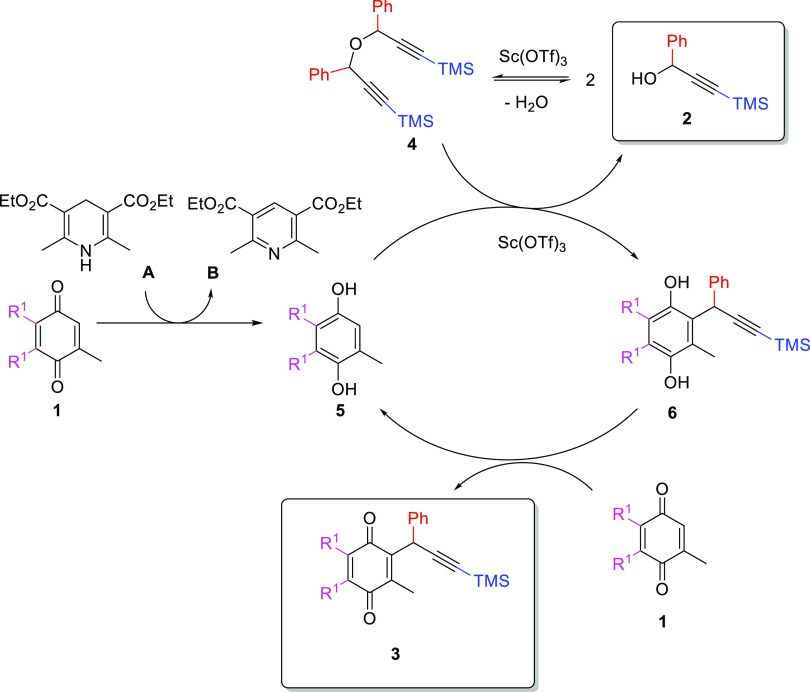
Proposed Reaction Mechanism for the Propargylation of CoQ0

To our delight, we did not observe formation of the desired product, which confirmed our hypothesis. To study the mechanism of this transformation in more detail, additional MS experiments of the reaction mixture were carried out to clarify its pathway. We noticed that the mass of dimer **4** (MW 413.17) appears in the raw reaction mixture (liquid chromatography mass spectrometry analysis of the raw reaction mixture), which supports our hypothesis. Based on the experiments and studies performed in the literature, a plausible reaction pathway of the process is depicted in Scheme 4. The presented transformation proceeds in a similar manner to the previous one presented by Li^23,24^ and previously described by us in aldehyde allylation that involves dimeric forms of allyl alcohols.^27^

A proposed reaction mechanism begins with the reduction of CoQ0 **1** by Hantzsch ester **A** to hydroquinone **5**. Separately, Sc(OTf)_3_ catalyzes the reversible formation of dimeric intermediate **4** from propargyl alcohol **2**, which is a starting material. The equilibrium that generates **4** from **2** requires two equivalents of the former and releases one molecule of water. Subsequently, electrophilic aromatic substitution catalyzed by Sc(OTf)_3_ takes place between hydroquinone **5** and dimer **4** or its carbocationic intermediate. This process leads to the formation of the hydroquinone derivative **6** and also re-generates one molecule of **2**. Then, a redox chain reaction occurs, in which the hydrogen atom is transferred between intermediate **6** and p-quinone **1** to form final product **3** and hydroquinone **5**, which participate in the next catalytic cycle. In this way, a small amount of Hantzsch ester **A** is only necessary to initiate the process at the beginning of the reaction.

## Conclusions

In summary, we have disclosed the first direct propargylation protocol for the synthesis of CoQ0. The given protocol showed a broad substrate scope, relatively mild reaction conditions, and good to excellent results. The presented studies depicted that propargylation of CoQ0 can be achieved in one single step from simple reagents without the need for its preliminary functionalization, which is excellent in terms of atom economy. We showed that many structurally varied propargyl alcohols can be converted using a 10 mol % Sc(OTf)_3_ catalyst in the presence of 5 mol % Hantzsch ester. In addition, a mechanistic experiment revealed the role of the catalyst and led to the proposed mechanism of this transformation. Performed experiments gave rise to the fact that reaction involves formation of dimeric propargylic intermediates and runs through a redox chain reaction. We believe that the application of this concept in other contexts will lead to the discovery of new synthetically useful reactions, while many quinones are important from a medicinal and biochemical point of view. Further studies toward a detailed mechanism, its stereoselective variant, and broader exploration of the presented strategy are currently in progress in our laboratory.

## Experimental Section

### General Information

Aldehydes, acetylenes, 2,3-dimethoxy-5-methyl-p-benzoquinone, diethyl 1,4-dihydro-2,6-dimethyl-3,5-pyridinedicarboxylate, and other reagents were purchased from Sigma Aldrich, Alfa Aesar, TCI, or ABCR and used without further purification. All reactions involving air-and moisture-sensitive materials were performed under an argon atmosphere in oven-dried glassware with magnetic stirring. Solvents were dried prior to use. Tetrahydrofuran (THF) and PhMe were distilled from Na and benzophenone and CH_2_Cl_2_ from CaH_2_. Column chromatography was performed with Kiesel gel (230–400 mesh). Analytical thin-layer chromatography was performed with 60 F254 aluminum plates of silica gel (Merck) with UV light visualization and charring with aqueous KMnO_4_ or Pancaldi reagent [(NH_4_)_6_MoO_4_, Ce(SO_4_)_2_, H_2_SO_4_, and H_2_O]. NMR analyses were performed with Bruker 400 MHz Avance III, Bruker DRX 500 Avance, or Varian 200 MHz spectrometers. Chemical shifts are calibrated using residual solvent signals (CDCl_3_: δ(H) = 7.26, δ(C) = 77.16) or TMS and are reported in ppm. Infrared spectra (IR) were recorded on a FT-IR-1600-Perkin Elmer spectrophotometer and are reported in frequency of absorption cm^–1^. High-resolution mass spectra were in general recorded on ESI-MS-TOF (MicrOTOF II, Bruker, Germany). When heating is indicated in the procedure, the reaction was performed using an aluminum block with a thermocouple and Heidolph hotplate.

#### General Procedure A for Synthesis of 1-Aryl-3-(trimethylsilyl)prop-2-yn-1-ol’s

Solution of trimethylsilylacetylene (0.83 mL, 6.0 mmol, 1.2 equiv) in anhydrous THF (10 mL) was cooled to −78 °C, 2 M solution of *n*-butyllithium in hexanes (2.8 mL, 5.5 mmol; 1.1 equiv) was added dropwise, and solution was stirred with cooling under an Ar atmosphere for 1 h. Then, solution of benzaldehyde (0.51 mL, 5.0 mmol, 1.0 equiv) in anhydrous THF (5 mL) was added dropwise and solution was warmed to room temperature for 0.5 h. Then, water (20 mL) was added for 2 h and the mixture was extracted with EtOAc (3 × 30 mL). Combined organic phases was washed with brine (20 mL), dried over anhydrous Na_2_SO_4_, filtered, and concentrated by rotary evaporation. The residue was purified by silica flash column chromatography using *n*-hexane/EtOAc as a solvent system.

#### General Procedure B for Synthesis of 2,3-Dimethoxy-5-methyl-6-(1-phenyl-3-(trimethylsilyl)prop-2-yn-1-yl)cyclohex-2-ene-1,4-dione

A 4 mL screw cap vial was charged with 2,3-dimethoxy-5-methyl-p-benzoquinone (27 mg, 0.15 mmol, 1.0 equiv), diethyl 1,4-dihydro-2,6-dimethyl-3,5-pyridinedicarboxylate (2.0 mg, 8.0 μmol, 0.05 equiv), and anhydrous MeCN (1 mL), and solution was stirred under Ar at rt. Then, 1-phenyl-3-(trimethylsilyl)prop-2-yn-1-ol (61 mg, 0.30 mmol; 2.0 equiv) was added for 30 min, followed by addition of Scandium(III) trifluoromethanesulfonate (7 mg, 0.015 mmol; 0.1 equiv). The mixture was heated to 60 °C and stirred for 24 h. The crude mixture was concentrated by rotary evaporation, and residue was purified by preparative TLC using hexane/acetone as a solvent system.

##### 1-Phenyl-3-(trimethylsilyl)prop-2-yn-1-ol (**2a**)

It was prepared according to the general procedure A. The product was obtained as light yellow oil (1.00 g, 98%). Eluent: *n*-hexane/EtOAc (9/1) ^1^H NMR (400 MHz, CDCl_3_) δ 7.56–7.54 (m, 2H), 7.41–7.36 (m, 2H), 7.35–7.31 (m, 1H), 5.45 (d, *J* = 6.4 Hz, 1H), 2.11 (d, *J* = 6.4 Hz, 1H), 0.21 (s, 3H) and correspond to literature data.^28^

##### 1-Phenylprop-2-yn-1-ol (**2b**)

1-Phenyl-3-(trimethylsilyl)prop-2-yn-1-ol **2a** (511 mg, 2.5 mmol; 1.0 equiv) was stirred with potassium carbonate (104 mg, 0.75 mmol; 0.3 eqiv.) in a mixture of MeOH/THF (1/1, v/v, 8 mL) at rt. Then, water (10 mL) was added for 2 h and the mixture was extracted with EtOAc (3 × 20 mL). Combined organic phases were washed with brine (20 mL) and dried over anhydrous Na_2_SO_4_, filtered, and concentrated by rotary evaporation. The crude product was used without further purification. Yellow oil (257 mg, 78%). ^1^H NMR (400 MHz, CDCl_3_) δ 7.57–7.55 (m, 2H), 7.42–7.32 (m, 3H), 5.47 (d, *J* = 2.3 Hz, 1H), 2.67 (d, *J* = 2.3 Hz, 1H) and correspond to literature data.^29^

##### 1-Phenylprop-2-yn-1-yl Acetate (**2c**)

1-Phenylprop-2-yn-1-ol **2b** (1.06 g, 8.0 mmol; 1.0 equiv) was stirred in anhydrous DCM (25 mL) under argon atmosphere, and tirethylamine (1.23 mL, 8.8 mmol; 1.1 equiv) was added. Solution was cooled in an ice-cold cooling bath, and acetic anhydride (1.1 mL, 12.0 mmol, 1.5 equiv) was added dropwise. The mixture was warmed to rt. and stirred overnight. Water (20 mL) was added, and the mixture was extracted with DCM (2 × 20 mL). Combined organic phases were washed with brine (20 mL) and dried over anhydrous Na_2_SO_4_, filtered, and concentrated by rotary evaporation. The compound was purified by column chromatography using *n*-hexane/EtOAc (95/5) as a solvent system. Light yellow oil (1.99 g, 89%). ^1^H NMR (400 MHz, CDCl_3_) δ 7.54–7.51 (m, 2H), 7.42–7.36 (m, 3H), 6.45 (d, *J* = 2.4 Hz, 1H), 2.64 (d, *J* = 2.4 Hz, 1H), 2.11 (s, 3H) and correspond to literature data.^30^

##### 4,4-Dimethyl-1-phenylpent-2-yn-1-ol (**2d**)

It was prepared according to the general procedure A. The product was obtained as light yellow oil (875 mg, 93%). Eluent: *n*-hexane/EtOAc (9/1) ^1^H NMR (400 MHz, CDCl_3_) δ 7.56–7.53 (m, 2H), 7.39–7.35 (m, 2H), 7.34–7.30 (m, 1H), 7.44 (d, *J* = 6.0 Hz, 1H), 2.03 (d, *J* = 6.0 Hz, 1H), 1.27 (s, 3H) and correspond to literature data.^31^

##### 5-Hydroxy-5-phenylpent-3-yn-1-yl Methanesulfonate (**2e**)

Methanesulfonic acid but-3-ynyl ester (741 mg, 5 mmol, 1.0 equiv), *n*-butyllithium (2.8 mL, 5.5 mmol; 1.1 equiv) and benzaldehyde (0.66 mL, 6.5 mmol, 1.3 equiv). Yellow oil (229 mg, 18%). Eluent: *n*-hexane/EtOAc (7/3) ^1^H NMR (400 MHz, CDCl_3_) δ 7.51 (d, *J* = 7.4 Hz, 2H), 7.39–7.30 (m, 3H), 5.43 (t, *J* = 2.5 Hz, 1H), 4.30 (t, *J* = 6.7 Hz, 2H), 2.97 (s, 3H), 2.73 (dt, *J* = 6.7, 2.5 Hz, 2H), 2.44 (d, *J* = 5.6 Hz, 1H). ^13^C{^1^H} NMR (100 MHz, CDCl_3_) δ 140.7, 128.6, 128.4, 126.5, 82.8, 81.5, 67.5, 64.6, 37.7, 20.2. IR (CHCl_3_, cm^–1^) 3509, 3062, 3030, 2937, 2232, 1455, 01353, 1173, 968, 903, 802, 701, 528. HRMS (ESI-TOF) *m*/*z*: [M + Na]^+^: calcd. for C_12_H_14_O_4_SNa 277.0510, found 277.0511.

#### 3-Cyclopropyl-1-phenylprop-2-yn-1-ol (**2f**)

It was prepared according to the general procedure A. The product was obtained as light yellow oil (814 mg, 95%). Eluent: *n*-hexane/EtOAc (9/1) ^1^H NMR (400 MHz, CDCl_3_) δ 7.53–7.51 (m, 2H), 7.39–7.29 (m, 3H), 5.42 (s, 1H), 2.07 (s, br, 1H), 7.35–7.28 (m, 1H), 0.82–0.76 (m, 2H), 0.75–0.71 (m, 2H) and correspond to literature data.^32^

##### 3-Cyclohexyl-1-phenylprop-2-yn-1-ol (**2g**)

It was prepared according to the general procedure A. The product was obtained as light yellow oil (973 mg, 91%). Eluent: *n*-hexane/EtOAc (9/1) ^1^H NMR (400 MHz, CDCl_3_) δ 7.57–7.54 (m, 2H), 7.39–7.29 (m, 3H), 5.46 (dd, *J* = 6.1, 2.1 Hz, 1H), 2.50–2.43 (m, 1H), 2.06 (d, *J* = 6.1 Hz, 1H), 1.85–1.80 (m, 2H), 1.73–1.69 (m, 2H), 1.52–1.43 (m, 3H), 1.35–1.26 (m, 3H) and correspond to literature data.^32^

##### 3-(Cyclohex-1-en-1-yl)-1-phenylprop-2-yn-1-ol **(2h)**

It was prepared according to the general procedure A. The product was obtained as a yellow solid (1.04 g, 98%). Eluent: *n*-hexane/EtOAc (9/1) ^1^H NMR (400 MHz, CDCl_3_) δ 7.57–7.54 (m, 2H), 7.40–7.35 (m, 2H), 7.34–7.30 (m, 1H), 6.18–6.15 (m, 1H), 5.57 (s, 1H), 2.32 (s, br, 1H), 2.18–2.08 (m, 4H), 1.68–1.56 (m, 4H) and correspond to literature data.^33^

##### 1,3-Diphenylprop-2-yn-1-ol (**2i**)

It was prepared according to the general procedure A. The product was obtained as yellow oil (980 mg, 94%). Eluent: *n*-hexane/EtOAc (9/1) ^1^H NMR (400 MHz, CDCl_3_) δ 7.64–7.61 (m, 2H), 7.49–7.46 (m, 2H), 7.43–7.39 (m, 2H), 7.37–7.35 (m, 1H), 7.34–7.30 (m, 3H), 5.70 (d, *J* = 6.2 Hz, 1H), 2.29 (d, *J* = 6.2 Hz, 1H) and correspond to literature data.^34^

##### 1-Phenyl-3-(*p*-tolyl)prop-2-yn-1-ol (**2j**)

It was prepared according to the general procedure A. The product was obtained as a yellow solid (958 mg, 96%). Eluent: *n*-hexane/EtOAc (9/1) ^1^H NMR (400 MHz, CDCl_3_) δ 7.62 (d, *J* = 7.5 Hz, 2H), 7.43–7.32 (m, 5H), 7.12 (d, *J* = 7.5 Hz, 2H), 5.69 (d, *J* = 6.1 Hz, 1H), 2.35 (s. 3H), 2.22 (d, *J* = 6.1 Hz, 1H) and correspond to literature data.^32^

##### 3-(4-Chlorophenyl)-1-phenylprop-2-yn-1-ol (**2ka**)

It was prepared according to the general procedure A. The product was obtained as a light orange solid (823 mg, 68%). Eluent: *n*-hexane/EtOAc (9/1) ^1^H NMR (400 MHz, CDCl_3_) δ 7.60 (d, *J* = 7.6 Hz, 2H), 7.43–7.34 (m, 5H), 7.30 (d, *J* = 7.6 Hz, 2H), 5.69 (d, *J* = 5.9 Hz, 1H), 2.24 (d, *J* = 5.9 Hz, 1H) and correspond to literature data.^34^

##### 3-(4-Bromophenyl)-1-phenylprop-2-yn-1-ol (**2kb**)

It was prepared according to the general procedure A. The product was obtained as yellow oil (1.08 g, 75%). Eluent: *n*-hexane/EtOAc (9/1) ^1^H NMR (400 MHz, CDCl_3_) δ 7.64–7.59 (m, 2H), 7.49–7.31 (m, 7H), 5.69 (dd, *J* = 9.1, 6.0 Hz, 1H), 2.24 (d, *J* = 6.0 Hz, 1H) and correspond to literature data.^35^

##### 3-(Naphthalen-2-yl)-1-phenylprop-2-yn-1-ol (**2l**)

It was prepared according to the general procedure A. The product was obtained as an off-white solid (1.23 g, 95%). Eluent: *n*-hexane/EtOAc (9/1) ^1^H NMR (400 MHz, CDCl_3_) δ 8.00 (d, *J* = 2.3 Hz, 1H), 7.39–7.35 (m, 1H), 7.83–7.77 (m, 3H), 7.68–7.35 (m, 2H), 7.53–7.47 (m, 3H), 7.46–7.41 (m, 2H), 5.75 (d, *J* = 6.1 Hz, 1H), 2.30 (d, *J* = 6.1 Hz, 1H) and correspond to literature data.^34^

##### 3-(Trimethylsilyl)prop-2-yn-1-ol (**2m**)

It was prepared according to the general procedure A. The product was obtained as colorless oil (596 mg, 93%). Eluent: *n*-hexane/EtOAc (9/1) ^1^H NMR (400 MHz, CDCl_3_) δ 4.26 (d, *J* = 5.9 Hz, 2H), 1.66 (t, *J* = 5.9 Hz, 1H), 0.17 (s, 9H) and correspond to literature data.^36^

##### 4-(Trimethylsilyl)but-3-yn-2-ol (**2n**)

It was prepared according to the general procedure A. The product was obtained as orange oil (631 mg, 89%). Eluent: *n*-hexane/EtOAc (9/1) ^1^H NMR (400 MHz, CDCl_3_) δ 4.51 (dt, *J* = 13.2, 2.8 Hz, 1H), 1.81 (d, *J* = 13.2 Hz, 1H), 1.44 (d, *J* = 2.8 Hz, 3H), 0.16 (s, 9H) and correspond to literature data.^37^

##### 4-Methyl-1-(trimethylsilyl)pent-1-yn-3-ol (**2o**)

It was prepared according to the general procedure A. The product was obtained as light yellow oil (801 mg, 94%). Eluent: *n*-hexane/EtOAc (9/1) ^1^H NMR (400 MHz, CDCl_3_) δ 4.15 (d, *J* = 6.2 Hz, 1H), 1.86 (dsep., *J* = 6.2, 1.1 Hz, 1H), 1.73 (s, br, 1H), 1.99 (t, *J* = 6.2 Hz, 6H), 0.17 (s, 9H) and correspond to literature data.^38^

##### 1,5-Bis(trimethylsilyl)penta-1,4-diyn-3-ol (**2p**)

It was prepared according to the general procedure A. The product was obtained as an orange solid (1.05 g, 94%). Eluent: *n*-hexane/EtOAc (9/1) ^1^H NMR (400 MHz, CDCl_3_) δ 5.09 (d, *J* = 6.8 Hz, 1H), 2.17 (d, *J* = 6.8 Hz, 1H), 0.19 (s, 18H) and correspond to literature data.^39^

##### 1-(4-(Trifluoromethyl)phenyl)-3-(trimethylsilyl)prop-2-yn-1-ol (**2r**)

It was prepared according to the general procedure A. The product was obtained as orange oil (470 mg, 35%). Eluent: *n*-hexane/EtOAc (9/1) ^1^H NMR (400 MHz, CDCl_3_) δ 7.68–7.63 (m, 4H), 5.51 (d, *J* = 6.1 Hz, 1H), 2.23 (d, *J* = 6.1 Hz, 1H), 0.21 (s, 9H) and correspond to literature data.^40^

##### 1-(4-Chlorophenyl)-3-(trimethylsilyl)prop-2-yn-1-ol (**2s**)

It was prepared according to the general procedure A. The product was obtained as an off-white solid (1.08 g, 91%). Eluent: *n*-hexane/EtOAc (9/1) ^1^H NMR (400 MHz, CDCl_3_) δ 7.47 (d, *J* = 8.4 Hz, 2H), 7.35 (d, *J* = 8.4 Hz, 2H), 5.42 (d, *J* = 4.8 Hz, 1H), 2.17 (d, *J* = 4.8 Hz, 1H), 0.20 (s, 9H) and correspond to literature data.^41^

##### 1-(4-Bromophenyl)-3-(trimethylsilyl)prop-2-yn-1-ol (**2t**)

It was prepared according to the general procedure A. The product was obtained as an off-white solid (1.03 g, 72%). Eluent: *n*-hexane/EtOAc (9/1) ^1^H NMR (400 MHz, CDCl_3_) δ 7.51 (d, *J* = 8.3 Hz, 2H), 7.41 (d, *J* = 8.3 Hz, 2H), 5.41 (s, 1H), 2.17 (s, br, 1H), 0.20 (s, 9H) and correspond to literature data.^42^

##### 1-(*p*-Tolyl)-3-(trimethylsilyl)prop-2-yn-1-ol (**2u**)

It was prepared according to the general procedure A. The product was obtained as a light yellow solid (1.00 g, 92%). Eluent: *n*-hexane/EtOAc (9/1) ^1^H NMR (400 MHz, CDCl_3_) δ 7.43 (d, *J* = 8.1 Hz, 2H), 7.19 (d, *J* = 8.1 Hz, 2H), 5.41 (d, *J* = 5.2 Hz, 1H), 2.36 (s, 3H), 2.08 (d, *J* = 5.2 Hz, 1H), 0.20 (s, 9H) and correspond to literature data.^43^

##### 1-(4-Methoxyphenyl)-3-(trimethylsilyl)prop-2-yn-1-ol (**2v**)

It was prepared according to the general procedure A. The product was obtained as yellow oil (1.06 g, 91%). Eluent: *n*-hexane/EtOAc (9/1) ^1^H NMR (400 MHz, CDCl_3_) δ 7.47 (d, *J* = 2.2 Hz, 2H), 6.90 (d, *J* = 2.2 Hz, 2H), 5.40 (d, *J* = 1.4 Hz, 1H), 3.81 (s, 3H), 2.07 (s, br, 1H), 0.20 (s, 9H) and correspond to literature data.^28^

##### 1-(3-Nitrophenyl)-3-(trimethylsilyl)prop-2-yn-1-ol (**2w**)

It was prepared according to the general procedure A. The product was obtained as orange oil (1.08 g, 87%). Eluent: *n*-hexane/EtOAc (4/1) ^1^H NMR (400 MHz, CDCl_3_) δ 8.44 (t, *J* = 2.2 Hz, 1H), 8.19 (ddd, *J* = 8.0, 2.2, 1.0 Hz, 1H), 7.88 (dt, *J* = 8.0, 1.0 Hz, 1H), 7.56 (t, *J* = 8.0 Hz, 1H), 5.55 (d, *J* = 5.6 Hz, 1H), 2.35 (d, *J* = 5.6 Hz, 1H), 0.22 (s, 9H) and correspond to literature data.^49^

##### 1-(*m*-Tolyl)-3-(trimethylsilyl)prop-2-yn-1-ol (**2x**)

It was prepared according to the general procedure A. The product was obtained as yellow oil (791 mg, 72%). Eluent: *n*-hexane/EtOAc (9/1) ^1^H NMR (400 MHz, CDCl_3_) δ 7.36–7.33 (m, 2H), 7.29–7.27 (m, 1H), 7.14 (d, *J* = 7.5 Hz, 1H), 5.42 (d, *J* = 6.1 Hz, 1H), 2.37 (s, 3H), 2.12 (d, *J* = 6.1 Hz, 1H), 0.21 (s, 9H) and correspond to literature data.^44^

##### 1-(3-Chloro-4-methoxyphenyl)-3-(trimethylsilyl)prop-2-yn-1-ol (**2y**)

It was prepared according to the general procedure A. The product was obtained as a light yellow solid (1.32 g, 98%). Eluent: *n*-hexane/EtOAc (9/1) ^1^H NMR (400 MHz, CDCl_3_) δ 7.67 (d, *J* = 8.6 Hz, 1H), 6.92 (d, *J* = 2.6 Hz, 1H), 6.85 (dd, *J* = 5.6, 2.6 Hz, 1H), 5.76 (s, 1H), 3.80 (s, 3H), 2.45 (s, br, 1H), 0.20 (s, 9H). ^13^C{^1^H} NMR (100 MHz, CDCl_3_) δ 160.4, 134.0, 130.4, 129.7, 115.3, 113.3, 104.8, 91.6, 62.1, 55.8, 0.2. IR (CHCl_3_, cm^–1^) 3401, 2960, 2899, 2838, 2173, 1605, 1496, 1284, 12,580, 1234, 1044, 844, 761. HRMS (ESI-TOF) *m*/*z*: [M + Na]^+^: calcd. for C_13_H_17_ClO_2_SiNa 291.0588, found 291.0584.

##### 1-(*o*-Tolyl)-3-(trimethylsilyl)prop-2-yn-1-ol (**2z**)

It was prepared according to the general procedure A. The product was obtained as light yellow oil (988 mg, 90%). Eluent: *n*-hexane/EtOAc (9/1) ^1^H NMR (400 MHz, CDCl_3_) δ 7.66–7.63 (m, 1H), 7.25–7.22 (m, 2H), 7.20–7.16 (m, 1H), 5.60 (d, *J* = 1.7 Hz, 1H), 2.44 (s, 3H), 2.06 (d, *J* = 1.7 Hz, 1H), 0.20 (s, 9H) and correspond to literature data.^42^

##### 1-(2-Chlorophenyl)-3-(trimethylsilyl)prop-2-yn-1-ol (**2aa**)

It was prepared according to the general procedure A. The product was obtained as yellow oil (1.07 g, 90%). Eluent: *n*-hexane/EtOAc (9/1) ^1^H NMR (400 MHz, CDCl_3_) δ 7.76 (dd, *J* = 7.6, 1.8 Hz, 1H), 7.38 (dd, *J* = 7.6, 1.8 Hz, 1H), 7.34–7.27 (m, 2H), 5.82 (s, 1H), 2.39 (s, br, 1H), 0.20 (s, 9H) and correspond to literature data.^45,46^

##### 2-(1-Hydroxy-3-(trimethylsilyl)prop-2-yn-1-yl)phenol (**2ab**)

It was prepared according to the general procedure A. The product was obtained as a red solid (370 mg, 34%). Eluent: *n*-hexane/EtOAc (4/1) ^1^H NMR (400 MHz, CDCl_3_) δ 7.38 (dd, *J* = 7.8, 1.8 Hz, 1H), 7.26–7.22 (m, 1H), 6.93–6.89 (m, 2H), 5.67 (d, *J* = 5.4 Hz, 1H), 2.72 (d, *J* = 5.4 Hz, 1H), 0.22 (s, 9H) and correspond to literature data.^44^

##### 1-(Thiophen-2-yl)-3-(trimethylsilyl)prop-2-yn-1-ol (**2ac**)

It was prepared according to the general procedure A. The product was obtained as orange oil (1.02 g, 97%). Eluent: *n*-hexane/EtOAc (9/1) ^1^H NMR (400 MHz, CDCl_3_) δ 7.30 (dd, *J* = 5.1, 1.1 Hz, 1H), 7.18 (dt, *J* = 3.5, 1.1 Hz, 1H), 6.98 (dd, *J* = 5.1, 3.5 Hz, 1H), 5.63 (s, 1H), 0.22 (s, 9H) and correspond to literature data.^28^

##### 1-(Naphthalen-1-yl)-3-(trimethylsilyl)prop-2-yn-1-ol (**2ad**)

It was prepared according to the general procedure A. The product was obtained as orange oil (1.20 g, 94%). Eluent: *n*-hexane/EtOAc (9/1) ^1^H NMR (400 MHz, CDCl_3_) δ 9.30 (dd, *J* = 8.4, 1.2 Hz, 1H), 7.89–7.84 (m, 3H), 7.58–7.48 (m, 3H), 6.12 (s, 1H), 2.28 (s, br, 1H), 0.22 (s, 9H) and correspond to literature data.^47^

##### 1-(Naphthalen-2-yl)-3-(trimethylsilyl)prop-2-yn-1-ol (**2ae**)

It was prepared according to the general procedure A. The product was obtained as an orange solid (1.14 g, 90%). Eluent: *n*-hexane/EtOAc (9/1) ^1^H NMR (400 MHz, CDCl_3_) δ 7.99 (m, 1H), 7.88–7.83 (m, 3H), 7.65 (dd, *J* = 8.6, 1.7 Hz, 1H), 7.51–7.48 (m, 2H), 5.62 (d, *J* = 6.2 Hz, 1H), 2.24 (dd, *J* = 6.2, 1.7 Hz, 1H), 0.22 (s, 9H) and correspond to literature data.^28^

#### 2,3-Dimethoxy-5-methyl-6-(1-phenyl-3-(trimethylsilyl)prop-2-yn-1-yl)cyclohex-2-ene-1,4-dione (**3a**)

It was prepared according to the general procedure B. The product was obtained as orange oil (48 mg, 86%). Eluent: *n*-hexane/acetone (4/1) ^1^H NMR (400 MHz, CDCl_3_) δ 7.36–7.28 (m, 4H), 7.24–7.20 (m, 1H), 5.78 (s, 1H), 4.04 (s, 3H), 4.00 (s, 3H), 1.99 (s, 3H), 0.21 (s, 9H). ^13^C{^1^H} NMR (100 MHz, CDCl_3_) δ 184.7, 183.0, 144.6, 144.1, 141.8, 140.8, 137.7, 128.5, 127.1, 126.9, 102.7, 89.8, 61.3, 61.2, 32.9, 12.3, 0.0. IR (CHCl_3_, cm^–1^) 3032, 2959, 1898, 2173, 1651, 1612, 1494, 1454, 1283, 1251, 1058, 1041, 1028, 1009, 845, 761, 698. HRMS (ESI-TOF) *m*/*z*: [M + Na]^+^: calcd. for C_21_H_24_O_4_SiNa 391.1342, found 391.1349.

### Large-Scale Experiment

An argon-flushed flask equipped with a reflux condenser was charged with 2,3-dimethoxy-5-methyl-*p*-benzoquinone (273 mg, 1.5 mmol, 1.0 equiv), diethyl 1,4-dihydro-2,6-dimethyl-3,5-pyridinedicarboxylate (19.0 mg, 75.0 μmol, 0.05 equiv), and anhydrous MeCN (10 mL), and the solution was stirred under Ar at rt. Then, 1-phenyl-3-(trimethylsilyl)prop-2-yn-1-ol (613 mg, 3.0 mmol; 2.0 equiv) was added for 30 min, followed by the addition of Scandium(III) trifluoromethanesulfonate (74 mg, 0.15 mmol; 0.1 equiv). The mixture was heated to 60 °C and stirred for 24 h. The crude mixture was concentrated by rotary evaporation, and residue was purified by flash column chromatography (FCC) using hexane/acetone (4/1) as a solvent system. The product was obtained as orange oil (471 mg, 85%).

#### 5-(4,4-Dimethyl-1-phenylpent-2-yn-1-yl)-2,3-dimethoxy-6-methylcyclohex-2-ene-1,4-dione (**3d**)

It was prepared according to the general procedure B. The product was obtained as orange oil (47 mg, 89%). Eluent: *n*-hexane/acetone (4/1) ^1^H NMR (400 MHz, CDCl_3_) δ 7.35–7.33 (m, 2H), 7.30–7.26 (m, 2H), 7.23–7.18 (m, 1H), 5.69 (s, 1H), 4.04 (s, 3H), 4.00 (s, 3H), 1.98 (s, 3H), 1.27 (s, 9H). ^13^C{^1^H} NMR (100 MHz, CDCl_3_) δ 184.9, 183.3, 144.5, 144.1, 141.8, 141.5, 138.7, 128.4, 127.2, 126.7, 93.6, 75.4, 61.3, 61.1, 31.8, 31.1, 27.7, 12.2. IR (CHCl_3_, cm^–1^) 3475, 2968, 2212, 1769, 1650, 1611, 1493, 1452, 1282, 1242, 1200, 1147, 1099, 1006, 990, 741, 700. HRMS (ESI-TOF) *m*/*z*: [M + Na]^+^: calcd. for C_22_H_24_O_4_Na 375.1572, found 375.1573.

#### 5-(3,4-Dimethoxy-6-methyl-2,5-dioxocyclohex-3-en-1-yl)-5-phenylpent-3-yn-1-yl methanesulfonate (**3e**)

It was prepared according to the general procedure B. The product was obtained as orange oil (32 mg, 50%). Eluent: *n*-hexane/acetone (7/3) ^1^H NMR (400 MHz, CDCl_3_) δ 7.34–7.27 (m, 4H), 7.24–7.20 (m, 1H), 5.69 (s, 1H), 4.32 (t, *J* = 6.8 Hz, 2H), 4.02 (s, 3H), 3.99 (s, 3H), 3.02 (s, 3H), 2.75 (dt, *J* = 6.8, 2.4 Hz, 2H), 1.96 (s, 3H). ^13^C{^1^H} NMR (100 MHz, CDCl_3_) δ 184.5, 182.9, 144.5, 144.1, 141.6, 140.9, 137.8, 128.5, 127.1, 127.0, 79.8, 79.1, 67.2, 61.3, 37.7, 32.2, 20.2, 12.4. IR (CHCl_3_, cm^–1^) 2945, 1652, 1612, 1356, 1281, 1174, 991, 960, 740. HRMS (ESI-TOF) *m*/*z*: [M + Na]^+^: calcd. for C_21_H_22_O_7_SNa 441.0984, found 441.0988.

#### 5-(3-Cyclopropyl-1-phenylprop-2-yn-1-yl)-2,3-dimethoxy-6-methylcyclohex-2-ene-1,4-dione (**3f**)

It was prepared according to the general procedure B. The product was obtained as orange oil (32 mg, 63%). Eluent: *n*-hexane/acetone (4/1) ^1^H NMR (400 MHz, CDCl_3_) δ 7.33–7.26 (m, 4H), 7.22–7.18 (m, 1H), 5.66 (s, 1H), 4.02 (s, 3H), 3.99 (s, 3H), 1.95 (s, 3H), 1.35–1.27 (m, 1H), 0.80–0.76 (m, 2H), 0.72–0.68 (m, 2H). ^13^C{^1^H} NMR (100 MHz, CDCl_3_) δ 185.5, 183.9, 145.2, 144.8, 142.3, 142.2, 139.3, 129.1, 127.9, 127.5, 88.9, 72.8, 62.0, 32.8, 13.0, 8.9, 0.4. IR (CHCl_3_, cm^–1^) 3290, 3007, 2947, 2237, 1651, 1612, 1493, 1451, 1282, 1264, 1200, 1144, 1100, 991, 741, 699. HRMS (ESI-TOF) *m*/*z*: [M + Na]^+^: calcd. for C_21_H_20_O_4_Na 359.1259, found 359.1265.

#### 5-(3-Cyclohexyl-1-phenylprop-2-yn-1-yl)-2,3-dimethoxy-6-methylcyclohex-2-ene-1,4-dione (**3g**)

It was prepared according to the general procedure B. The product was obtained as orange oil (32 mg, 63%). Eluent: *n*-hexane/acetone (4/1) ^1^H NMR (400 MHz, CDCl_3_) δ 7.37–7.35 (m, 2H), 7.30–7.26 (m, 2H), 7.22–7.18 (m, 1H), 5.72 (s, 1H), 4.0, (s, 3H), 3.99 (s, 3H), 2.49–2.43 (m, 1H), 1.98 (s, 3H), 1.86–1.81 (m, 2H), 1.74–1.69 (m, 2H), 1.55–1.44 (m, 3H), 1.35–1.30 (m, 3H). ^13^C{^1^H} NMR (100 MHz, CDCl_3_) δ 184.8, 183.2, 144.5, 144.1, 141.8, 141.5, 138.7, 128.4, 127.2, 126.7, 89.4, 61.3, 32.9, 32.8, 32.0, 29.3, 25.9, 24.9. IR (CHCl_3_, cm^–1^) 3447, 2930, 2853, 2200, 1770, 1722, 1651, 1611, 1493, 1449, 1283, 1242, 1200, 1141, 1101, 740, 699. HRMS (ESI-TOF) *m*/*z*: [M + Na]^+^: calcd. for C_24_H_26_O_4_Na 401.1729, found 401.1735.

#### 5-(3-(Cyclohex-1-en-1-yl)-1-phenylprop-2-yn-1-yl)-2,3-dimethoxy-6-methylcyclohex-2-ene-1,4-dione (**3h**)

It was prepared according to the general procedure B. The product was obtained as orange oil (25 mg, 44%). Eluent: *n*-hexane/acetone (4/1) ^1^H NMR (400 MHz, CDCl_3_) δ 7.37–7.35 (m, 2H), 7.31–7.27 (m, 2H), 7.23–7.19 (m, 1H), 6.14–6.11 (m, 1H), 5.84 (s, 1H), 4.03 (s, 3H), 3.99 (s, 3H), 2.17–2.15 (m, 2H), 2.11–2.09 (m, 2H), 1.99 (s, 3H), 1.68–1.58 (m, 4H). ^13^C{^1^H} NMR (100 MHz, CDCl_3_) δ 184.7, 183.1, 144.5, 144.1, 141.6, 141.4, 138.3, 134.9, 128.4, 127.2, 126.8, 120.5, 86.7, 83.6, 61.3, 32.5, 29.3, 25.6, 22.3, 21.5, 12.3. IR (CHCl_3_, cm^–1^) 3445, 2935, 2855, 2214, 1718, 1652, 1611, 1450, 1270, 1242, 1198, 1131, 1075, 737, 701. HRMS (ESI-TOF) *m*/*z*: [M + Na]^+^: calcd. for C_24_H_24_O_4_Na 399.1572, found 399.1577.

#### 5-(1,3-Diphenylprop-2-yn-1-yl)-2,3-dimethoxy-6-methylcyclohex-2-ene-1,4-dione (**3i**)

It was prepared according to the general procedure B. The product was obtained as orange oil (47 mg, 84%). Eluent: *n*-hexane/acetone (4/1) ^1^H NMR (400 MHz, CDCl_3_) δ 7.48–7.42 (m, 4H), 7.34–7.30 (m, 5H), 7.26–7.22 (m, 1H), 5.97 (s, 1H), 4.05 (s, 3H), 4.01 (s, 3H), 2.07 (s, 3H). ^13^C{^1^H} NMR (100 MHz, CDCl_3_) δ 184.6, 183.1, 144.6, 144.1, 141.8, 141.1, 138.0, 131.7, 128.6, 128.3, 127.2, 127.0123.0 86.6, 84.8, 61.3, 32.6, 12.5. IR (CHCl_3_, cm^–1^) 3453, 2947, 1956, 1722, 1651, 1611, 1492, 1450, 1269, 1421, 1200, 1145, 1101, 1015, 757, 695. HRMS (ESI-TOF) *m*/*z*: [M + Na]^+^: calcd. for C_24_H_20_O_4_Na 395.1259, found 395.1263.

#### 2,3-Dimethoxy-5-methyl-6-(1-phenyl-3-(*p*-tolyl)prop-2-yn-1-yl)cyclohex-2-ene-1,4-dione (**3j**)

It was prepared according to the general procedure B. The product was obtained as orange oil (53 mg, 92%). Eluent: *n*-hexane/acetone (4/1) ^1^H NMR (400 MHz, CDCl_3_) δ 7.23–7.21 (m, 2H), 7.17–7.15 (m, 2H), 7.13–1.09 (m, 2H), 7.05–7.01 (m, 1H), 6.92 (d, *J* = 8.4 Hz, 2H), 5.75 (s, 1H), 3.84 (s, 3H), 3.870 (s, 3H), 2.15 (s, 3H), 1.85 (s, 3H). ^13^C{^1^H} NMR (100 MHz, CDCl_3_) δ 184.9, 183.3, 144.8, 144.3, 142.0, 141.4, 138.6, 138.6, 131.8, 129.3, 128.8, 127.4, 127.1, 120.1, 86.0, 85.1, 61.5, 61.5, 32.8, 21.6, 12.6. IR (CHCl_3_, cm^–1^) 3451, 2945, 1796, 1721, 1651, 1608, 1450, 1414, 1272, 1239, 1183, 1106, 1076, 819, 739, 700. HRMS (ESI-TOF) *m*/*z*: [M + Na]^+^: calcd. for C_25_H_22_O_4_Na 409.1416, found 409.1417.

#### 5-(3-(4-Chlorophenyl)-1-phenylprop-2-yn-1-yl)-2,3-dimethoxy-6-methylcyclohex-2-ene-1,4-dione (**3ka**)

It was prepared according to the general procedure B. The product was obtained as orange oil (57 mg, 93%). Eluent: *n*-hexane/acetone (4/1) ^1^H NMR (400 MHz, CDCl_3_) δ 7.42–7.38 (m, 4H), 7.34–7.28 (m, 4H), 7.26–7.22 (m, 1H), 5.95 (s, 1H), 4.04 (s, 3H), 4.01 (s, 3H), 2.05 (s, 3H). ^13^C{^1^H} NMR (100 MHz, CDCl_3_) δ 184.6, 183.0, 144.6, 144.1, 141.8, 140.9, 137.8, 134.4, 132.9, 128.7, 128.6, 127.2, 127.1, 121.5, 87.7, 83.7, 61.3, 32.7, 12.5. IR (CHCl_3_, cm^–1^) 3456, 3060, 2946, 2199, 1650, 1611, 1490, 1451, 1269, 1092, 1012, 830, 738, 700. HRMS (ESI-TOF) *m*/*z*: [M + Na]^+^: calcd. for C_24_H_19_ClO_4_Na 429.0870, found 429.0876.

#### 5-(3-(4-Bromophenyl)-1-phenylprop-2-yn-1-yl)-2,3-dimethoxy-6-methylcyclohex-2-ene-1,4-dione (**3kb**)

It was prepared according to the general procedure B. The product was obtained as orange oil (55 mg, 81%). Eluent: *n*-hexane/acetone (4/1) ^1^H NMR (400 MHz, CDCl_3_) δ 7.49–7.40 (m, 4 H), 7.34–7.31 (m, 4H), 7.26–7.23 (m, 1H), 5.96 (d, *J* = 10.8 Hz, 1H), 4.05 (s, 3H), 4.01 (s, 3H), 2.06 (d, *J* = 6.8 Hz, 3H). ^13^C{^1^H} NMR (100 MHz, CDCl_3_) δ 184.6, 183.0, 144.6, 144.2, 141.8, 141.1, 140.8, 138.0, 137.7, 133.1, 131.7, 131.6, 128.6, 128.4, 128.3, 127.2, 127.1, 127.0, 123.0, 122.6, 121.9, 87.9, 86.6, 84.8, 83.3, 61.3, 32.7, 12.5. IR (CHCl_3_, cm^–1^) 3453, 3060, 2947, 1707, 1651, 1611, 1489, 1451, 1269, 1242, 1200, 1145, 1100, 1072, 1011, 826, 738, 698. HRMS (ESI-TOF) *m*/*z*: [M – H]^−^: calcd. for C_24_H_18_BrO_4_ 449.0388, found 449.0384.

#### 2,3-Dimethoxy-5-methyl-6-(3-(naphthalen-2-yl)-1-phenylprop-2-yn-1-yl)cyclohex-2-ene-1,4-dione (**3l**)

It was prepared according to the general procedure B. The product was obtained as orange oil (59 mg, 94%). Eluent: *n*-hexane/acetone (4/1) ^1^H NMR (400 MHz, CDCl_3_) δ 7.99 (s, 1H), 7.82–7.78 (m, 3H), 7.52–7.46 (m, 5H), 7.36–7.32 (m, 2H), 7.26–7.24 (m, 1H), 6.03 (s, 1H), 4.06 (s, 3H), 4.02 (s, 3H), 2.11 (s, 3H). ^13^C{^1^H} NMR (100 MHz, CDCl_3_) δ 184.7, 183.1, 144.6, 144.2, 141.9, 141.1, 138.0, 133.0, 132.8, 131.5, 128.6, 128.4, 128.0, 127.8, 127.7, 127.3, 127.0, 126.7, 126.6, 120.3, 86.9, 85.2, 61.3, 32.8, 12.5. IR (CHCl_3_, cm^–1^) 3460, 3058, 2944, 1650, 1611, 1493, 1452, 1273, 1197, 1144, 1102, 1079, 740, 700. HRMS (ESI-TOF) *m*/*z*: [M + Na]^+^: calcd. for C_28_H_22_O_4_Na 445.1416, found 445.1420.

#### 5-(1-(4-Chlorophenyl)-3-(trimethylsilyl)prop-2-yn-1-yl)-2,3-dimethoxy-6-methylcyclohex-2-ene-1,4-dione (**3s**)

It was prepared according to the general procedure B. The product was obtained as orange oil (42 mg, 70%). Eluent: *n*-hexane/acetone (4/1) ^1^H NMR (400 MHz, CDCl_3_) δ 7.27 (s, 4H), 5.71 (s, 1H), 4.03 (s, 3H), 4.00 (s, 3H), 1.98 (s, 3H), 0.20 (s, 9H). ^13^C{^1^H} NMR (100 MHz, CDCl_3_) δ 184.6, 183.1, 144.8, 144.2, 142.1, 140.5, 136.4, 133.0, 128.8, 128.7, 102.3, 90.4, 61.5, 32.7, 12.5, 0.1. IR (CHCl_3_, cm^–1^) 3290, 2955, 2176, 1651, 1612, 1490, 1278, 1250, 1200, 1146, 1095, 845, 663. HRMS (ESI-TOF) *m*/*z*: [M + Na]^+^: calcd. for C_21_H_23_ClO_4_SiNa 425.0952, found 425.0959.

#### 5-(1-(4-Bromophenyl)-3-(trimethylsilyl)prop-2-yn-1-yl)-2,3-dimethoxy-6-methylcyclohex-2-ene-1,4-dione (**3t**)

It was prepared according to the general procedure B. The product was obtained as orange oil (63 mg, 94%). Eluent: *n*-hexane/acetone (4/1) ^1^H NMR (400 MHz, CDCl_3_) δ 7.42 (d, *J* = 8.6 Hz, 2H), 7.22 (dd, *J* = 8.6, 1.0 Hz, 2H), 5.69 (t, *J* = 1.0 Hz, 1H), 4.03 (s, 3H), 4.00 (s, 3H), 1.98 (s, 3H), 0.20 (s, 9H). ^13^C{^1^H} NMR (100 MHz, CDCl_3_) δ 184.6, 183.1, 144.8, 144.2, 142.1, 140.4, 137.0, 131.8, 129.1, 121.1, 102.3, 90.4, 61.5, 32.8, 12.6, 0.1. IR (CHCl_3_, cm^–1^) 3497, 2958, 2174, 1652, 1612, 1486, 1250, 1011, 845, 761. HRMS (ESI-TOF) *m*/*z*: [M + Na]^+^: calcd. for C_21_H_23_BrO_4_SiNa 469.0447, found 469.0441.

#### 2,3-Dimethoxy-5-methyl-6-(1-(*p*-tolyl)-3-(trimethylsilyl)prop-2-yn-1-yl)cyclohex-2-ene-1,4-dione (**3u**)

It was prepared according to the general procedure B. The product was obtained as orange oil (53 mg, 93%). Eluent: *n*-hexane/acetone (4/1) ^1^H NMR (400 MHz, CDCl_3_) δ 7.22 (d, *J* = 7.9 Hz, 2H), 7.10 (d, *J* = 7.9 Hz, 2H), 5.73 (s, 1H), 4.03 (s, 3H), 4.00 (s, 3H), 2.31 (s, 3H), 2.00 (s, 3H), 0.20 (s, 9H). ^13^C{^1^H} NMR (100 MHz, CDCl_3_) δ 184.8, 183.1, 144.6, 144.1, 141.7, 140.9, 136.6, 134.7, 129.2, 127.1, 103.1, 89.6, 61.3, 32.7, 21.0, 12.4, 0.0. IR (CHCl_3_, cm^–1^) 3501, 2956, 2174, 1662, 1612, 1511, 1281, 1250, 1200, 1145, 1102, 844, 781, 460. HRMS (ESI-TOF) *m*/*z*: [M + Na]^+^: calcd. for C_22_H_26_O_4_SiNa 405.1498, found 405.1499.

#### 2,3-Dimethoxy-5-methyl-6-(1-(*m*-tolyl)-3-(trimethylsilyl)prop-2-yn-1-yl)cyclohex-2-ene-1,4-dione (**3x**)

It was prepared according to the general procedure B. The product was obtained as orange oil (54 mg, 94%). Eluent: *n*-hexane/acetone (4/1) ^1^H NMR (400 MHz, CDCl_3_) δ 7.21–7.16 (m, 2H), 7.10 (s, 1H), 7.04–7.02 (m, 1H), 5.74 (s, 1H), 4.04 (s, 3H), 4.01 (s. 3H), 2.31 (s, 3H), 2.00 (s, 3H), 0.21 (s, 9H). ^13^C{^1^H} NMR (100 MHz, CDCl_3_) δ 184.8, 183.1, 144.6, 144.1, 141.8, 140.9, 138.2, 137.6, 128.4, 127.9, 127.7, 124.2, 103.0, 89.7, 61.3, 32.9, 21.5, 12.4, 0.0. IR (CHCl_3_, cm^–1^) 3496, 2956, 2174, 1651, 1611, 1454, 1281, 1250, 1200, 1145, 1101, 845, 762, 701. HRMS (ESI-TOF) *m*/*z*: [M + Na]^+^: calcd. for C_22_H_26_O_4_SiNa 405.1498, found 405.1494.

#### 5-(1-(3-Chloro-4-methoxyphenyl)-3-(trimethylsilyl)prop-2-yn-1-yl)-2,3-dimethoxy-6-methylcyclohex-2-ene-1,4-dione (**3y**)

It was prepared according to the general procedure B. The product was obtained as orange oil (60 mg, 93%). Eluent: *n*-hexane/acetone (4/1) ^1^H NMR (400 MHz, CDCl_3_) δ 7.84 (d, *J* = 8.6 Hz, 1H), 6.87–6.82 (m, 2H), 5.65 (s, 1H), 4.00 (s, 3H), 3.99 (s, 3H), 3.78 (s, 3H), 1.87 (s, 3H), 0.19 (s, 9H). ^13^C{^1^H} NMR (100 MHz, CDCl_3_) δ 184.6, 182.1, 144.5, 144.3, 140.5, 140.3, 133.3, 131.6, 127.2, 115.2, 112.6, 101.9, 90.4, 61.3, 55.6, 32.6, 11.9, 0.0. IR (CHCl_3_, cm^–1^) 2955, 2174, 1653, 1611, 1494, 1282, 1249, 1200, 1146, 1100, 1043, 846, 760. HRMS (ESI-TOF) *m*/*z*: [M + Na]^+^: calcd. for C_22_H_25_ClO_5_SiNa 455.1057, found 455.1050.

#### 2,3-Dimethoxy-5-methyl-6-(1-(*o*-tolyl)-3-(trimethylsilyl)prop-2-yn-1-yl)cyclohex-2-ene-1,4-dione (**3z**)

It was prepared according to the general procedure B. The product was obtained as orange oil (46 mg, 80%). Eluent: *n*-hexane/acetone (4/1) ^1^H NMR (400 MHz, CDCl_3_) δ 7.61–7.59 (m, 1H), 7.25–7.10 (m, 3H), 5.71 (s, 1H), 4.01 (s, 3H), 4.00 (s, 3H), 2.17 (s, 3H), 1.98 (s, 3H), 0.17 (s, 9H). ^13^C{^1^H} NMR (100 MHz, CDCl_3_) δ 184.5, 182.4, 144.7, 144.2, 142.2, 140.2, 136.1, 135.4, 131.0, 128.2, 127.4, 126.0, 103.0, 89.6, 61.4, 32.4, 20.3, 20.3, 12.6, 0.1. IR (CHCl_3_, cm^–1^) 3499, 2955, 2172, 1722, 1651, 1612, 1485, 1281, 1250, 1200, 1145, 1103, 844, 758. HRMS (ESI-TOF) *m*/*z*: [M + Na]^+^: calcd. for C_22_H_26_O_4_SiNa 405.1498, found 405.1503.

#### 5-(1-(2-Chlorophenyl)-3-(trimethylsilyl)prop-2-yn-1-yl)-2,3-dimethoxy-6-methylcyclohex-2-ene-1,4-dione (**3aa**)

It was prepared according to the general procedure B. The product was obtained as orange oil (18 mg, 30%). Eluent: *n*-hexane/acetone (4/1) ^1^H NMR (400 MHz, CDCl_3_) δ 7.97 (d, *J* = 7.8 Hz, 1H), 7.33–7.19 (m, 3H), 5.72 (s, 1H), 4.00 (s, 3H), 3.99 (s, 3H), 1.84 (s, 3H), 0.20 (s, 9H). ^13^C{^1^H} NMR (100 MHz, CDCl_3_) δ 184.5, 182.0, 144.6, 144.3, 140.5, 140.4, 135.3, 133.3, 131.0, 129.8, 128.8, 126.7, 101.5, 90.9, 61.4, 33.3, 11.9, 0.1. IR (CHCl_3_, cm^–1^) 3484, 2958, 2173, 1651, 1612, 1469, 1442, 1250, 1035, 844, 756. HRMS (ESI-TOF) *m*/*z*: [M + Na]^+^: calcd. for C_21_H_23_ClO_4_SiNa 425.0952, found 425.0951.

#### 2,3-Dimethoxy-5-methyl-6-(1-(thiophen-2-yl)-3-(trimethylsilyl)prop-2-yn-1-yl)cyclohex-2-ene-1,4-dione (**3ac**)

It was prepared according to the general procedure B. The product was obtained as brown oil (21 mg, 37%). Eluent: *n*-hexane/acetone (3/1) ^1^H NMR (400 MHz, CDCl_3_) δ 7.16 (dd, *J* = 5.0, 1.3 Hz, 1H), 6.96–6.95 (m, 1H), 6.91 (dd, *J* = 5.0, 3.5 Hz, 1H), 5.87 (d, *J* = 1.3 Hz, 1H), 4.02 (s, 3H), 4.00 (s, 3H), 2.12 (s, 3H), 0.20 (s, 9H). ^13^C{^1^H} NMR (100 MHz, CDCl_3_) δ 185.0, 182.8, 145.1, 144.4, 142.5, 141.8, 140.1, 127.0, 125.7, 125.0, 102.9, 89.6, 61.7, 29.7, 12.8 0.2. IR (CHCl_3_, cm^–1^) 2955, 2175, 1651, 1612, 1454, 1287, 1248, 1200, 1145, 1101, 845, 760, 700. HRMS (ESI-TOF) *m*/*z*: [M + Na]^+^: calcd. for C_19_H_22_O_4_SSiNa 397.0906, found 397.0903.

#### 2,3-Dimethoxy-5-methyl-6-(1-(naphthalen-1-yl)-3-(trimethylsilyl)prop-2-yn-1-yl)cyclohex-2-ene-1,4-dione (**3ad**)

It was prepared according to the general procedure B. The product was obtained as orange oil (58 mg, 93%). Eluent: *n*-hexane/acetone (4/1) ^1^H NMR (400 MHz, CDCl_3_) δ 7.88–7.84 (m, 2H), 7.79–7.73 (m, 2H), 7.48–7.42 (m, 3H), 6.31 (s, 1H), 3.04 (s, 3H), 3.99 (s, 3H), 1.92 (s, 3H), 0.20 (s, 9H). ^13^C{^1^H} NMR (100 MHz, CDCl_3_) δ 184.4, 182.8, 144.6, 144.2, 142.6, 140.6, 134.1, 132.8, 131.0, 129.0128.4, 126.5, 126.4, 125.8, 125.0, 123.5, 90.2, 61.4, 31.9, 12.4, 0.0. IR (CHCl_3_, cm^–1^) 2955, 2172, 1651, 1611, 1453, 1272, 1251, 1199, 1144, 1100, 846, 790. HRMS (ESI-TOF) *m*/*z*: [M + Na]^+^: calcd. for C_25_H_26_O_4_SiNa 441.1498, found 441.1501.

#### 2,3-Dimethoxy-5-methyl-6-(1-(naphthalen-2-yl)-3-(trimethylsilyl)prop-2-yn-1-yl)cyclohex-2-ene-1,4-dione (**3ae**)

It was prepared according to the general procedure B. The product was obtained as orange oil (59 mg, 94%). Eluent: *n*-hexane/acetone (4/1) ^1^H NMR (400 MHz, CDCl_3_) δ 7.89 (s, 1H), 7.81–7.75 (m, 3H), 7.50–7.43 (m, 2H), 7.35 (dd, *J* = 8.6, 1.9 Hz, 1H), 5.94 (s, 1H), 4.06 (s, 3H), 4.01 (s, 3H), 2.01 (s, 3H), 0.25 (s, 9H). ^13^C{^1^H} NMR (100 MHz, CDCl_3_) δ 184.6, 183.1, 144.6, 144.1, 142.1, 140.6, 135.0, 133.2, 128.3, 127.9, 126.3, 126.0, 125.9, 125.2, 102.8, 90.1, 61.3, 33.1, 12.4, 0.0. IR (CHCl_3_, cm^–1^) 3466, 2955, 2173, 1721, 1650, 1611, 1454, 1266, 1250, 1200, 1146, 1102, 846, 759. HRMS (ESI-TOF) *m*/*z*: [M + Na]^+^: calcd. for C_25_H_26_O_4_SiNa 441.1498, found 441.1497.

#### Synthesis of 1,1′-diphenyl-3,3′-bis(trimethylsilyl)-1,1′-dipropynyl Ether (**4**)

A 10 mL screw cap vial was charged with 1-phenyl-3-(trimethylsilyl)prop-2-yn-1-ol (122 mg, 0.60 mmol; 1.0 equiv) and anhydrous MeCN (4 mL) and Scandium(III) trifluoromethanesulfonate (30 mg, 0.06 mmol; 0.1 equiv). The mixture was heated to 60 °C and stirred for 24 h under argon. The crude mixture was concentrated by rotary evaporation, and residue was purified by FCC using hexane/acetone 98/2 as a solvent system. The product was obtained as light yellow oil (62 mg, 26%). ^1^H NMR (400 MHz, CDCl_3_) δ 7.62–7.45 (m, 4H), 7.44–7.33 (m, 6H), 5.68 and 5.30 (2 s, 2H), 0.29 and 0.24 (2 s, 18H). HRMS (ESI-TOF) *m*/*z*: [M + Na]^+^: calcd. for C_24_H_30_OSi_2_Na 413.1733, found 413.1740 and correspond to literature data.^48^

## Supporting Information Available

Copies of ^1^H and ^13^C NMR spectra and liquid chromatography mass spectrometry analysis of the raw reaction mixture for mechanistic studies (PDF)
