# Supplementary material for: Propargylation of CoQ0 through the Redox Chain Reaction
Source: J Org Chem. 2021 Dec 21;87(1):683–92. doi: 10.1021/acs.joc.1c02685 (PMC8749960; doi:10.1021/acs.joc.1c02685)
Supplement: Supplementary file 1 — jo1c02685_si_001.pdf [file jo1c02685_si_001.pdf]

Propargylation of CoQ0 through the Redox Chain Reaction

Robert Pawlowski, Maciej Stodulski*, and Jacek Mlynarski*

Institute of Organic Chemistry, Polish Academy of Sciences, Kasprzaka 44/52, 01-224 Warsaw, Poland

Supporting Information

Table of Contents

1. Copies of ^{1}H and ^{13}C NMR spectra S2

2. Mechanistic studies – LCMS analysis of raw reaction mixture S40

1. Copies of ^{1}H and ^{13}C NMR spectra

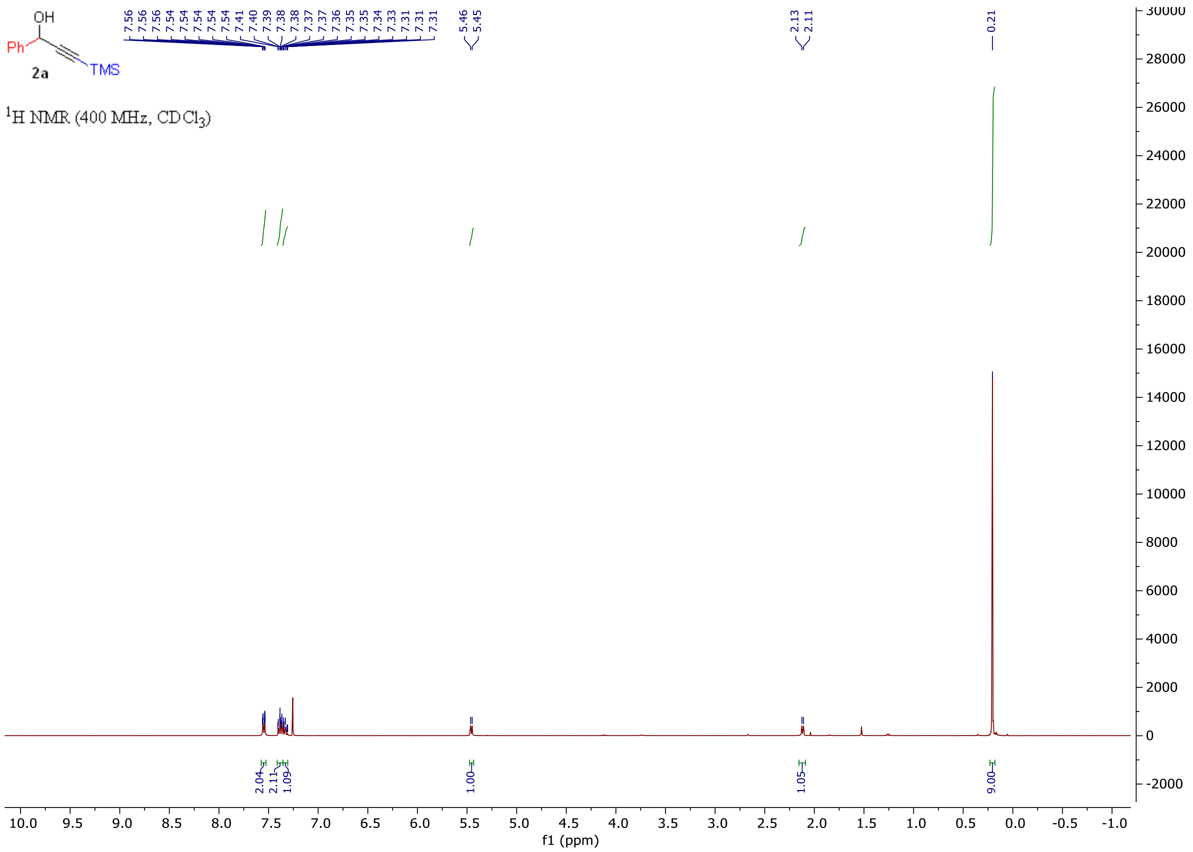

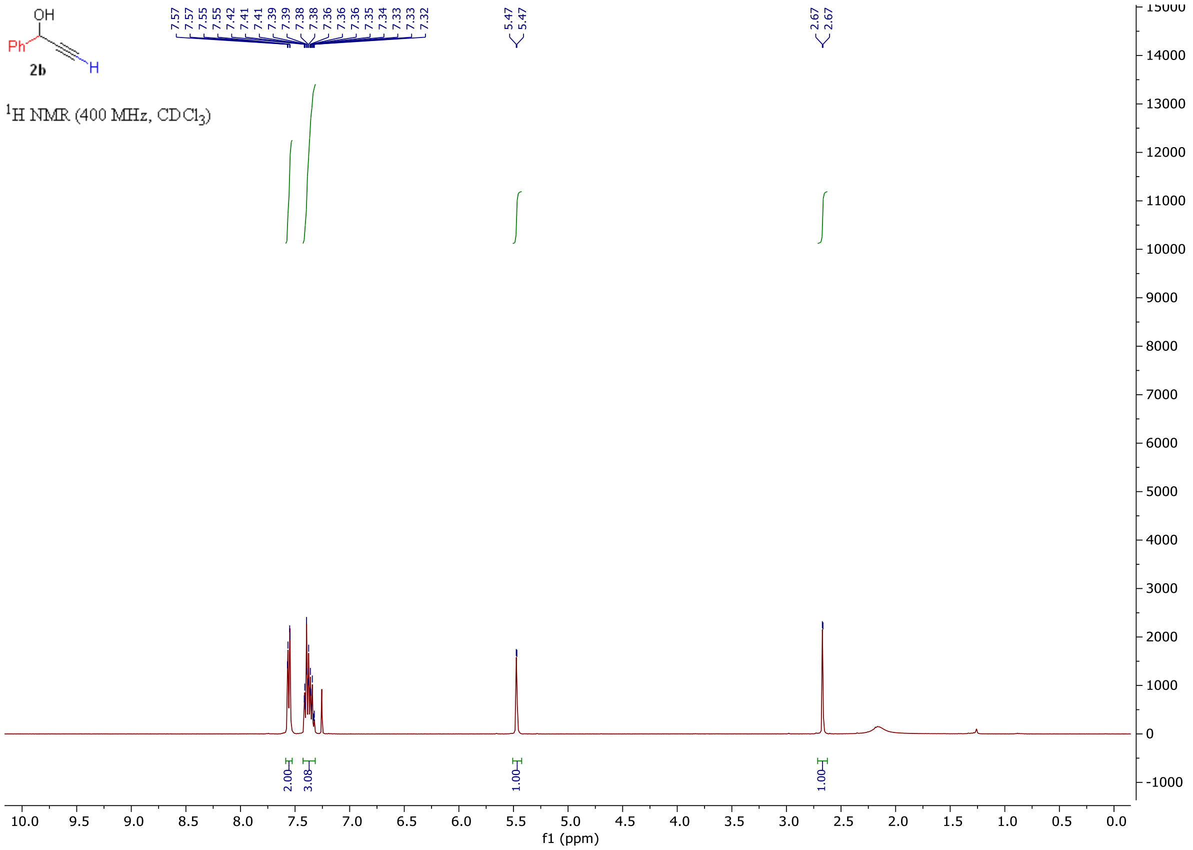

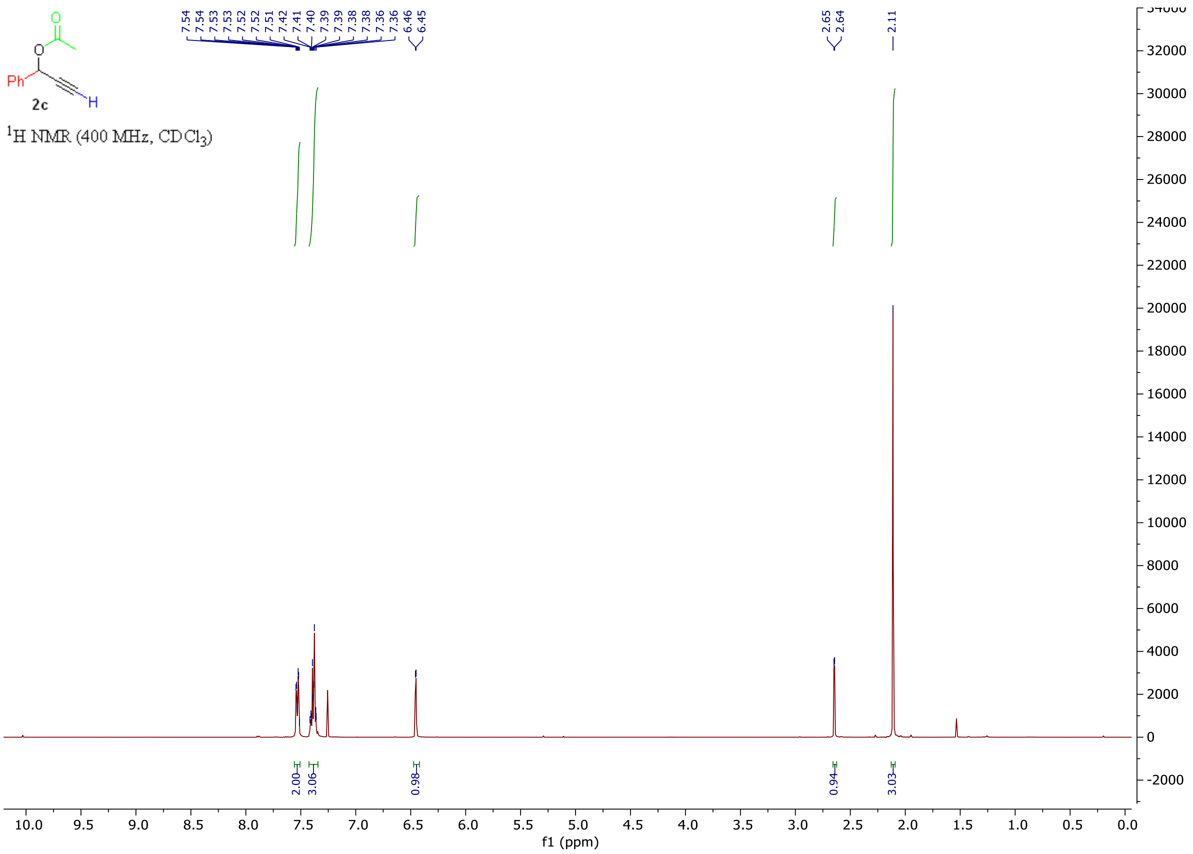
2c
1H NMR (400 MHz, CDCl3)
f1 (ppm)

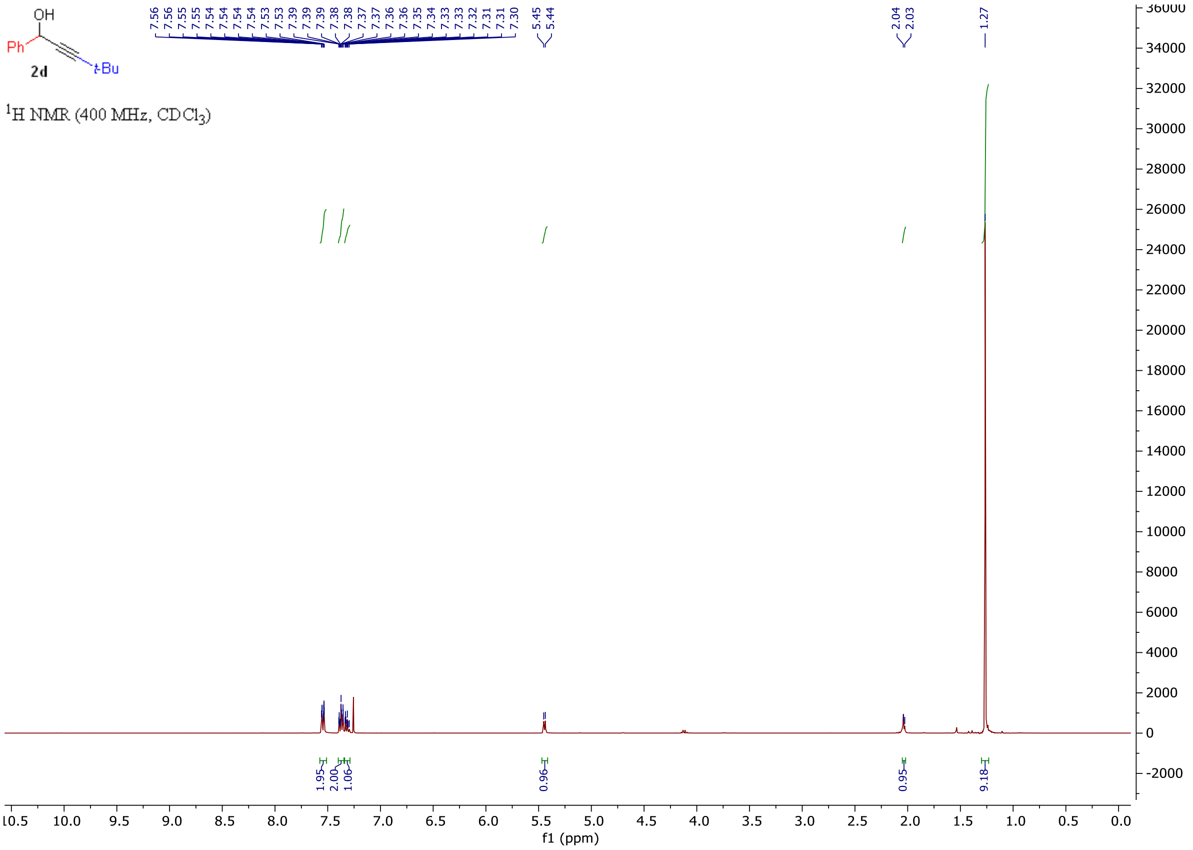
2d
1H NMR (400 MHz, CDCl3)
f1 (ppm)

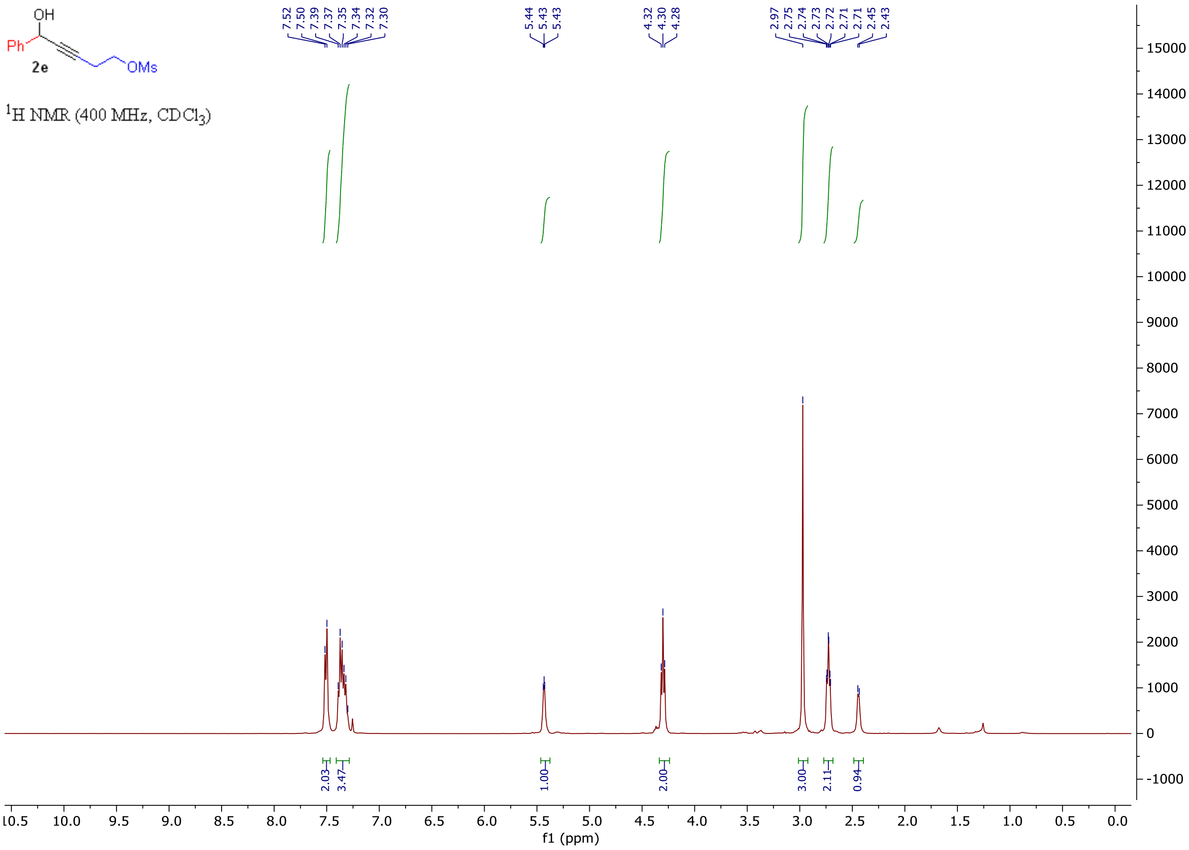
OH
Ph
2e
OMs
^{1}H NMR (400 MHz, CDCl$_3$)
7.52
7.50
7.39
7.37
7.35
7.34
7.32
7.30
5.44
5.43
5.43
4.32
4.30
4.28
2.97
2.75
2.74
2.73
2.72
2.71
2.71
2.45
2.43
2.03
3.47
1.00
2.00
3.00
2.11
0.94
f1 (ppm)

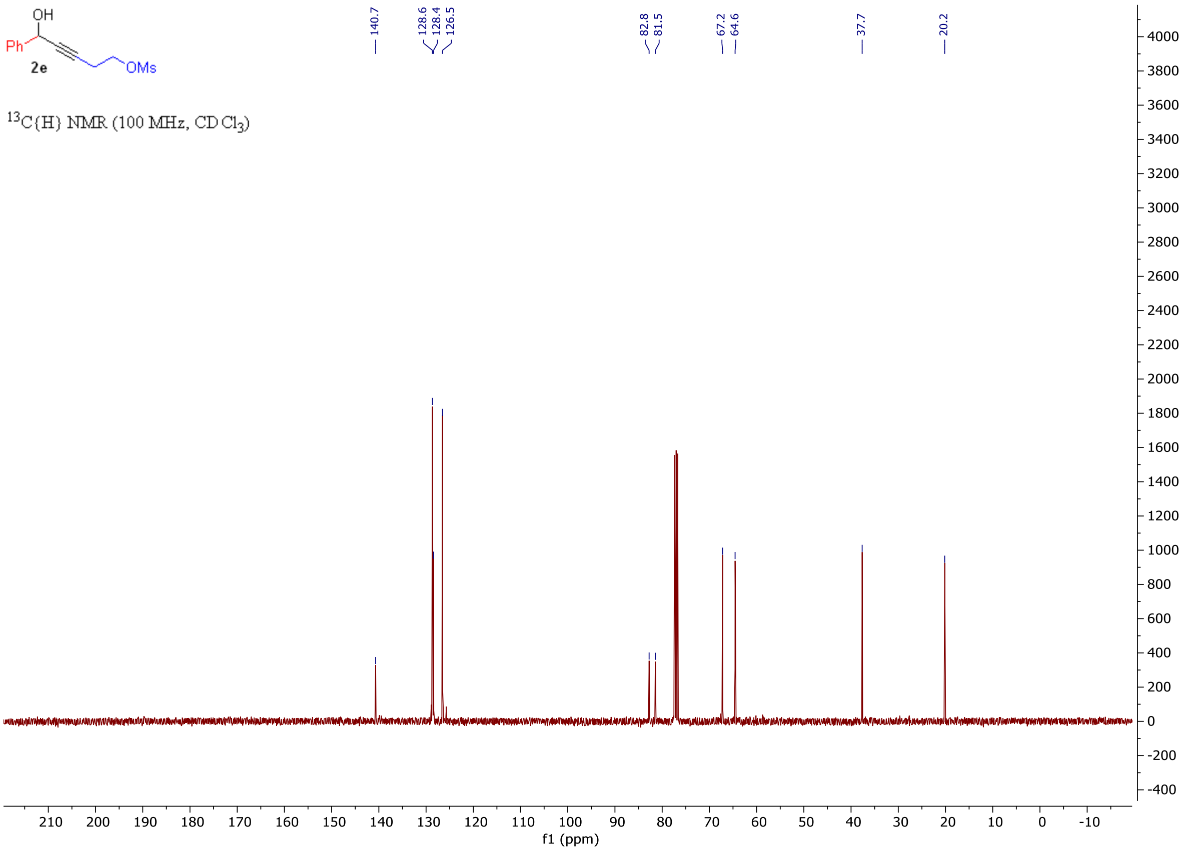
OH
Ph
2e
OMs
^{13}C{H} NMR (100 MHz, CDCl$_3$)
140.7
128.6
128.4
126.5
82.8
81.5
67.2
64.6
37.7
20.2
f1 (ppm)

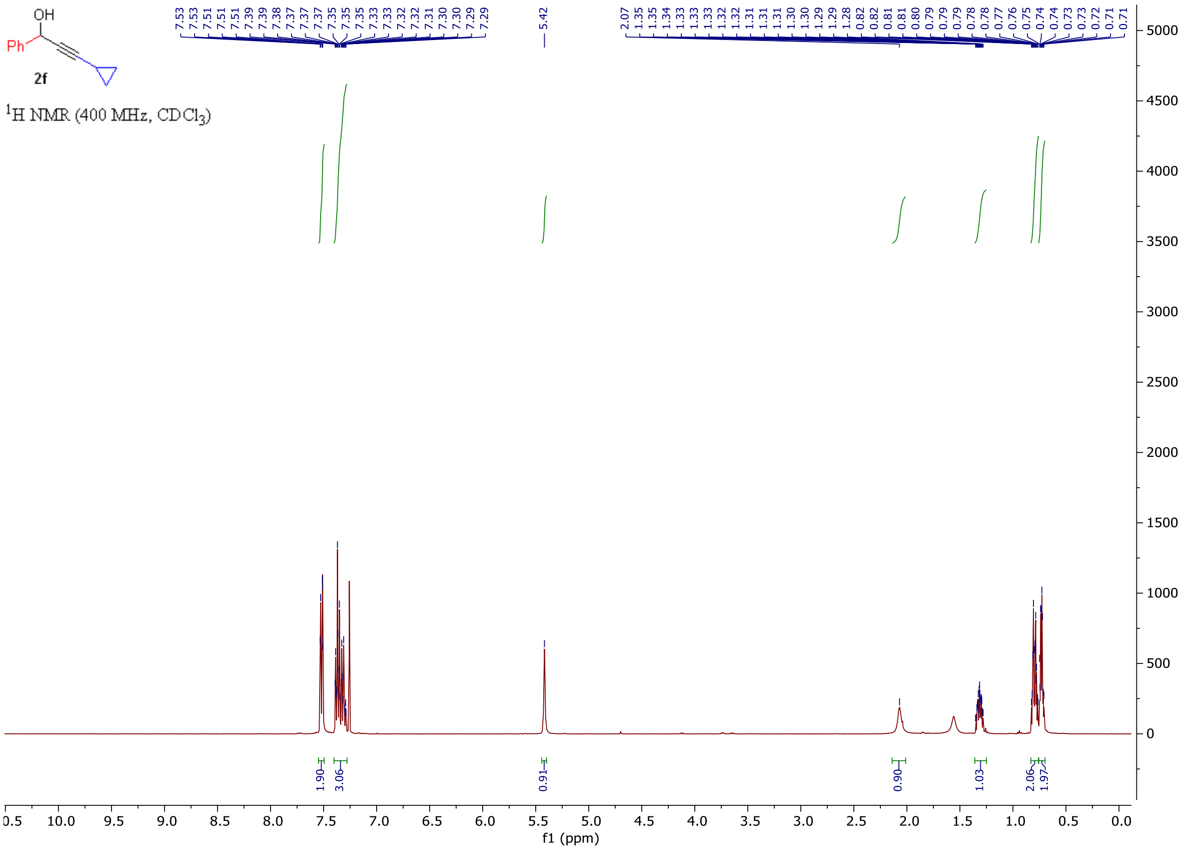
OH
Ph
2f
^{1}H NMR (400 MHz, $CDCl_3$)
f1 (ppm)

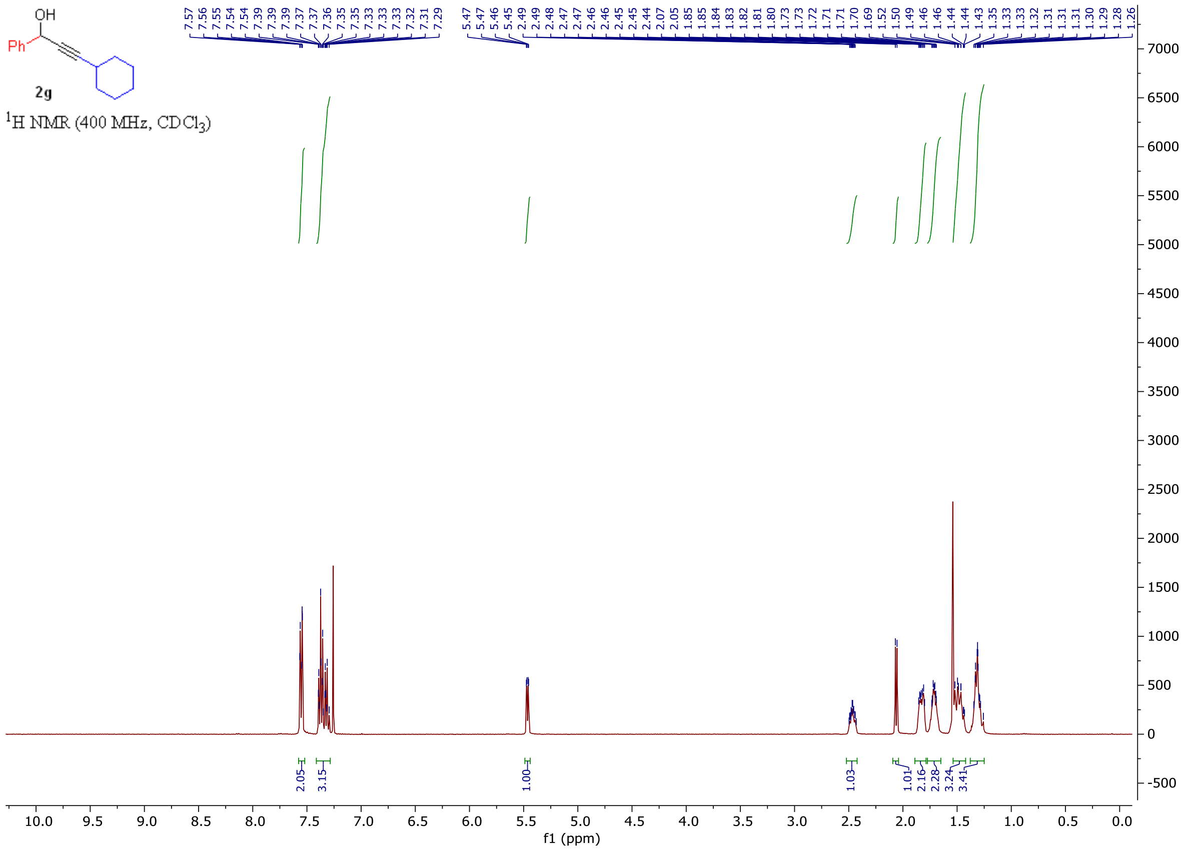
OH
Ph
2g
^{1}H NMR (400 MHz, $CDCl_3$)
f1 (ppm)

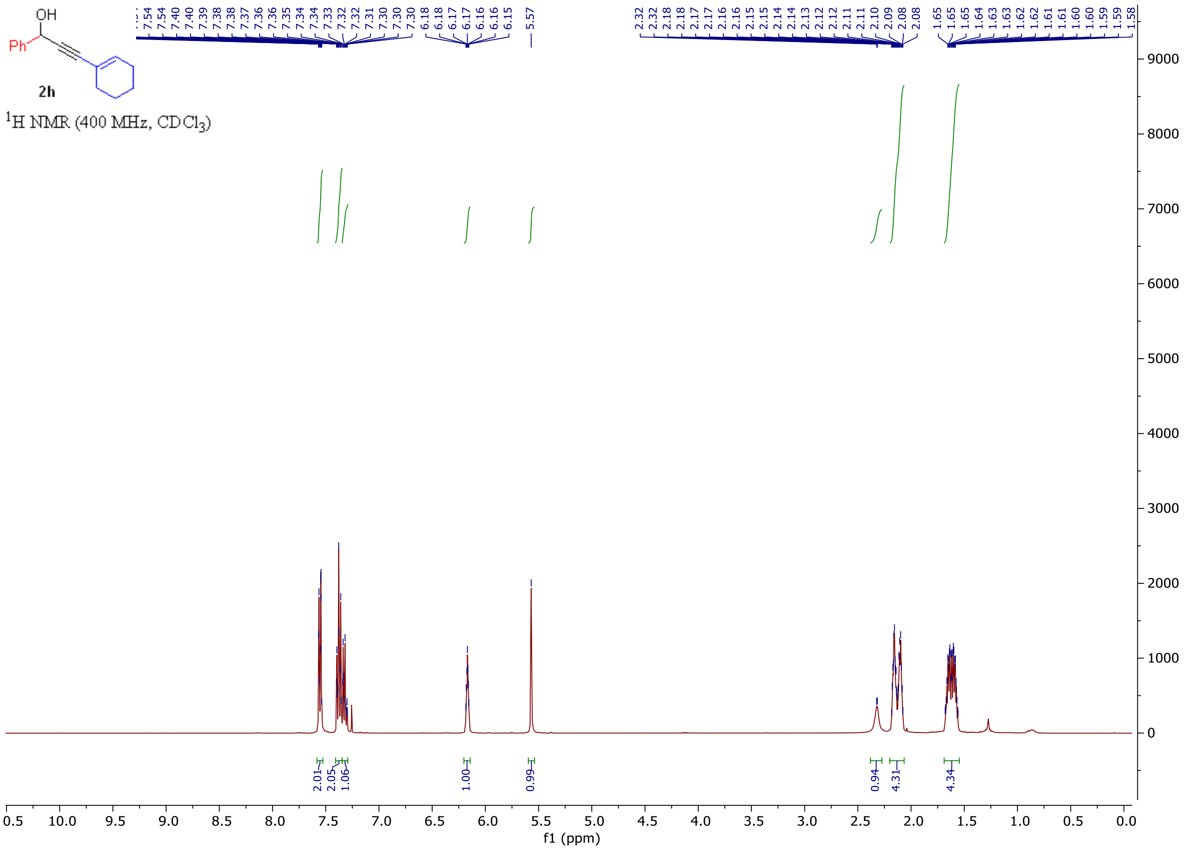
OH
Ph
2h
^{1}H NMR (400 MHz, $CDCl_3$)
f1 (ppm)

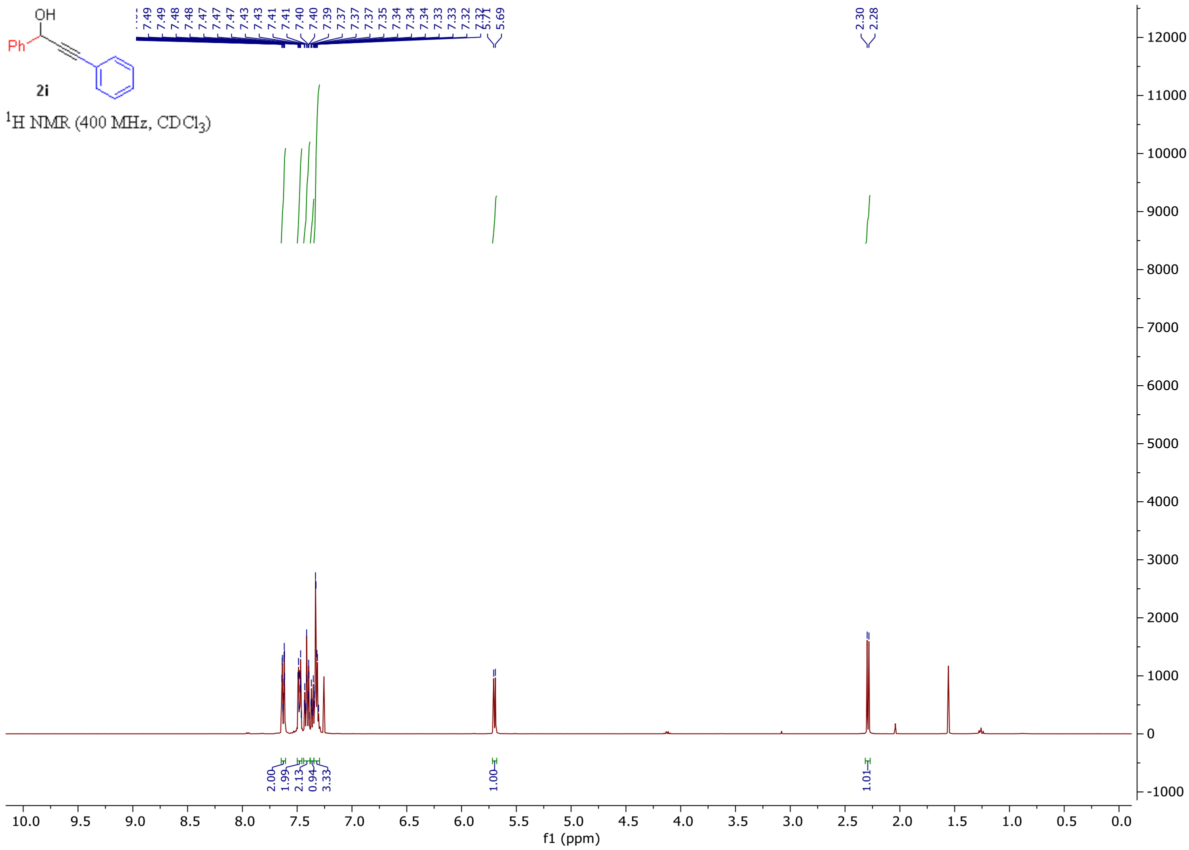
OH
Ph
2i
^{1}H NMR (400 MHz, $CDCl_3$)
f1 (ppm)

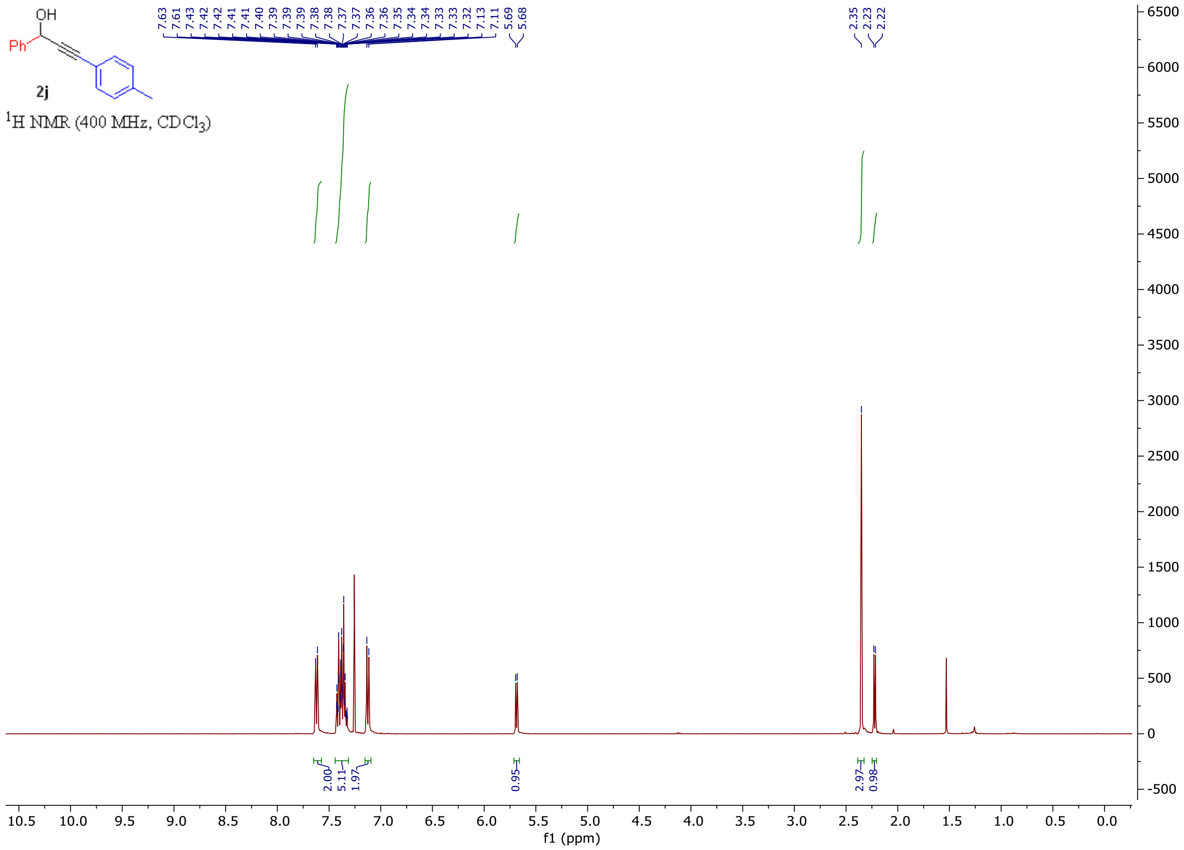

OH
Ph
2j
^{1}H NMR (400 MHz, CDCl$_3$)
7.63 7.61 7.43 7.42 7.42 7.41 7.41 7.40 7.39 7.39 7.39 7.38 7.38 7.37 7.37 7.36 7.36 7.35 7.34 7.34 7.33 7.33 7.32 7.13 7.11 5.69 5.68
2.35 2.23 2.22
2.00 5.11 1.97 0.95 2.97 0.98
f1 (ppm)

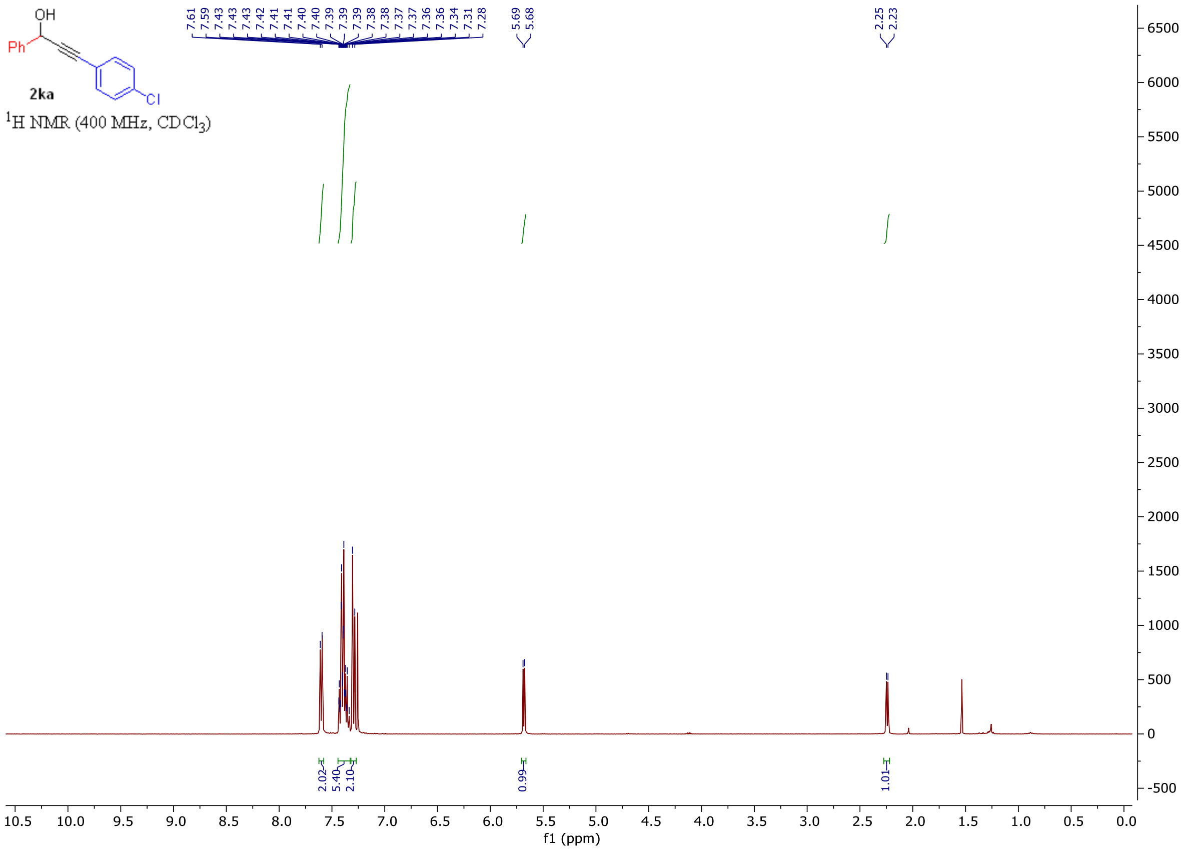

OH
Ph
Cl
2ka
^{1}H NMR (400 MHz, CDCl$_3$)
7.61 7.59 7.43 7.43 7.43 7.42 7.41 7.41 7.40 7.39 7.39 7.39 7.38 7.38 7.37 7.37 7.36 7.36 7.34 7.31 7.28 5.69 5.68
2.25 2.23
2.02 5.40 2.10 0.99 1.01
f1 (ppm)

2kb

^1H NMR (400 MHz, $CDCl_3$)

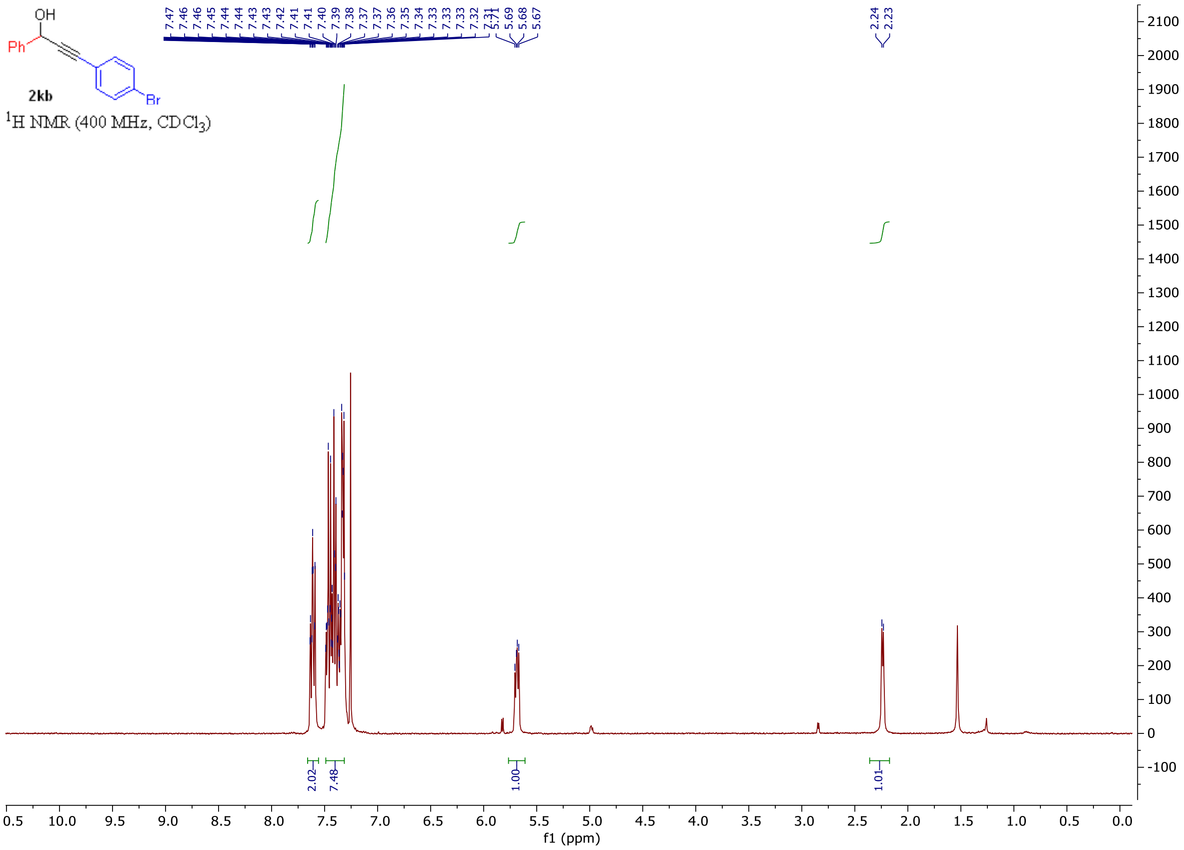

2l

^1H NMR (400 MHz, $CDCl_3$)

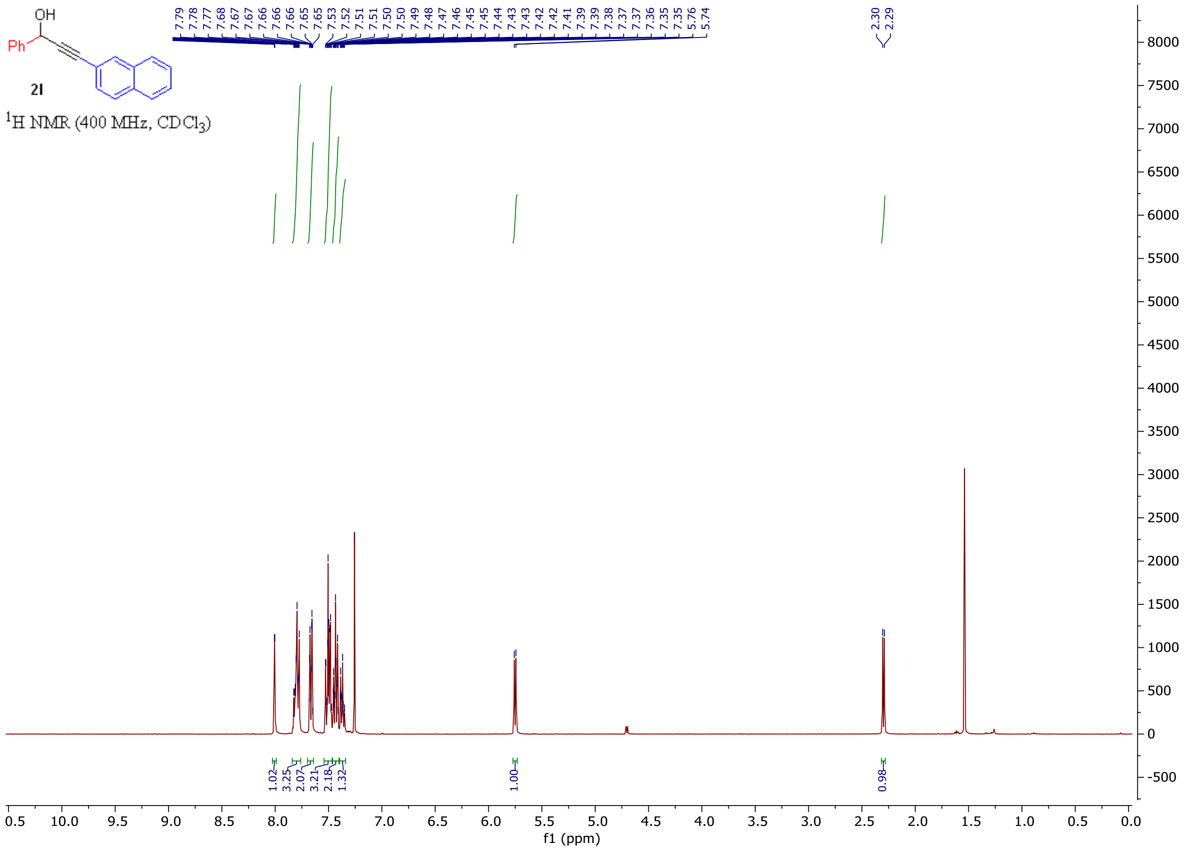

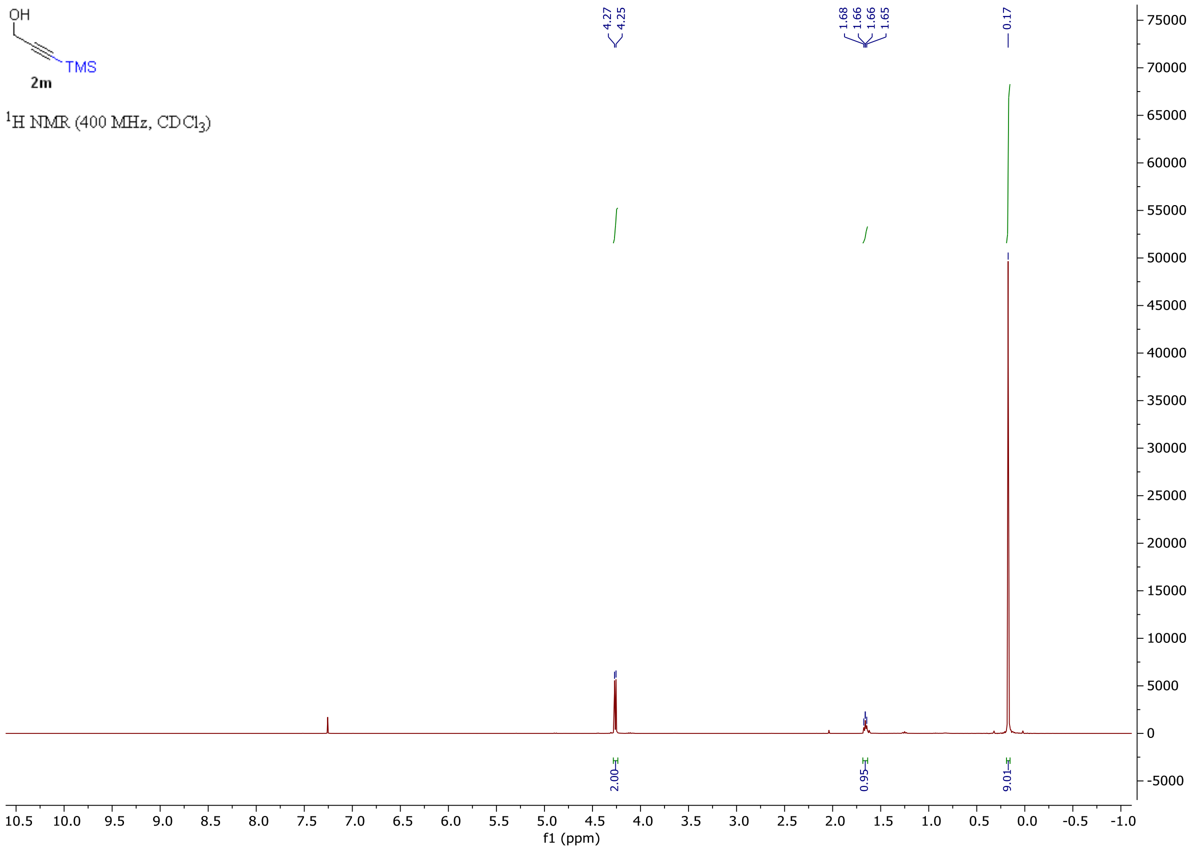
OH
TMS
2m
1H NMR (400 MHz, CDCl3)
4.27
4.25
1.68
1.66
1.66
1.65
0.17
2.00
0.95
9.01
f1 (ppm)

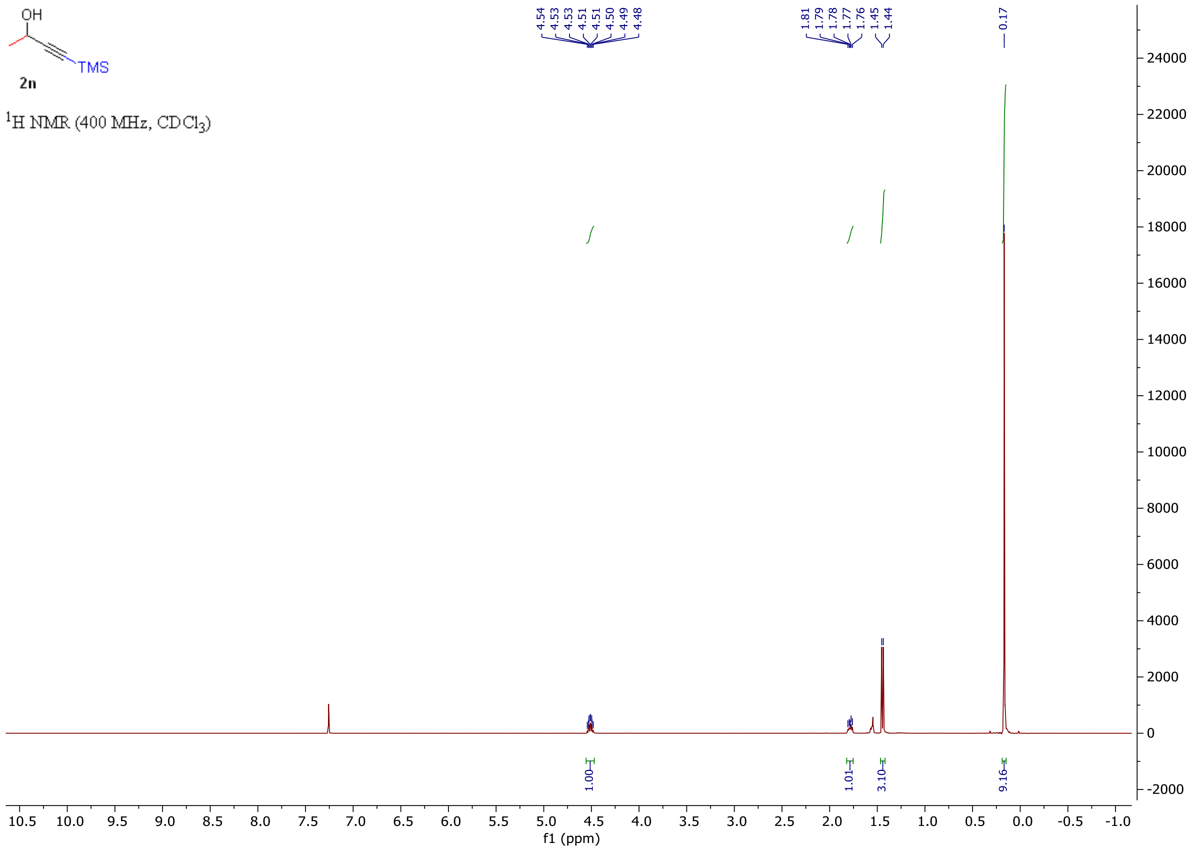
OH
TMS
2n
1H NMR (400 MHz, CDCl3)
4.54
4.53
4.53
4.51
4.51
4.50
4.49
4.48
1.81
1.79
1.78
1.77
1.76
1.45
1.44
0.17
1.00
1.01
3.10
9.16
f1 (ppm)

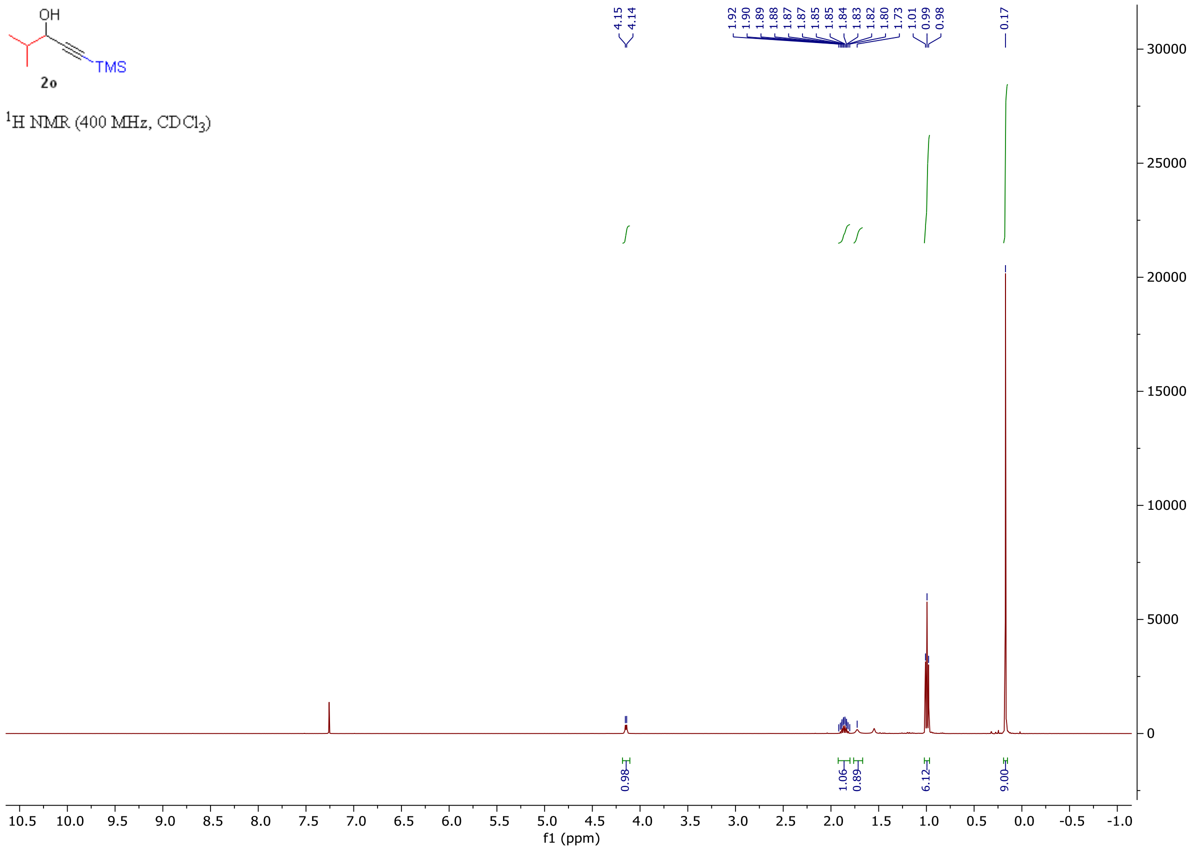
OH
TMS
2o
¹H NMR (400 MHz, CDCl3)
f1 (ppm)

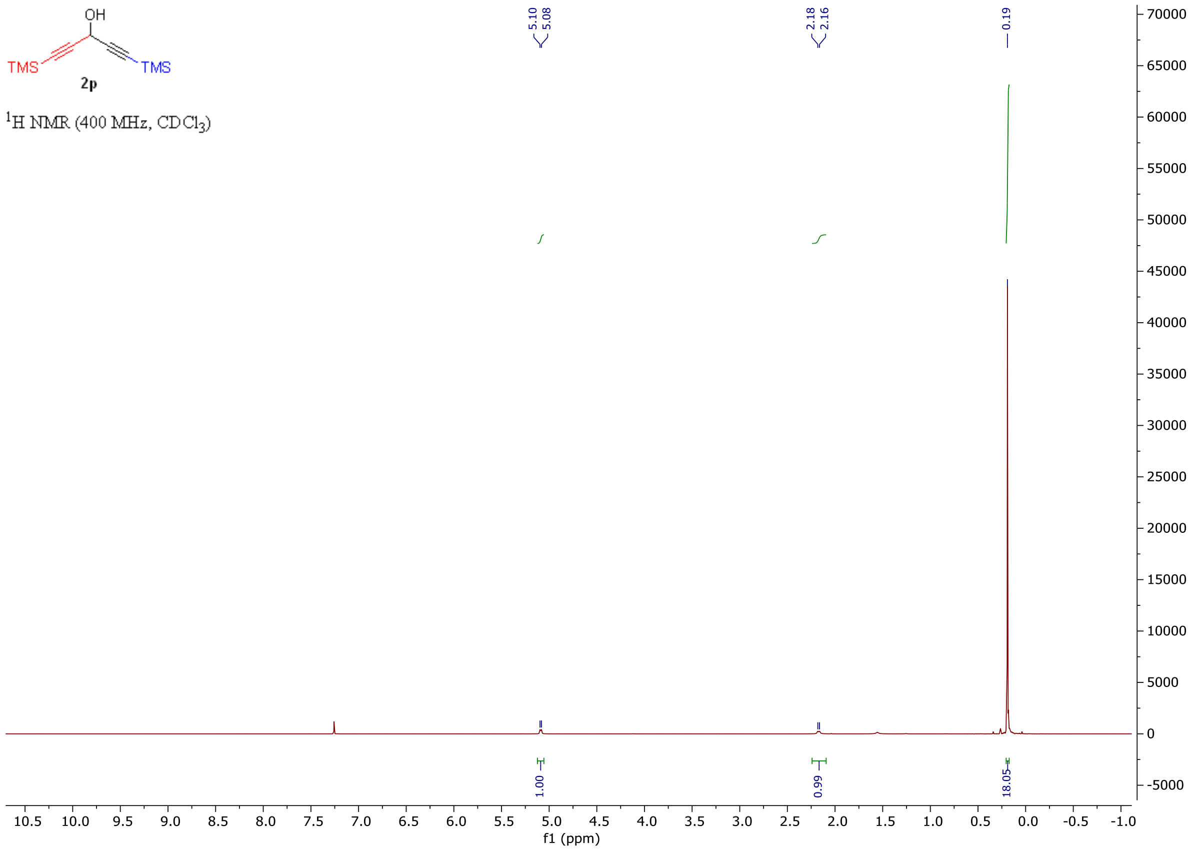
OH
TMS
TMS
2p
¹H NMR (400 MHz, CDCl3)
f1 (ppm)

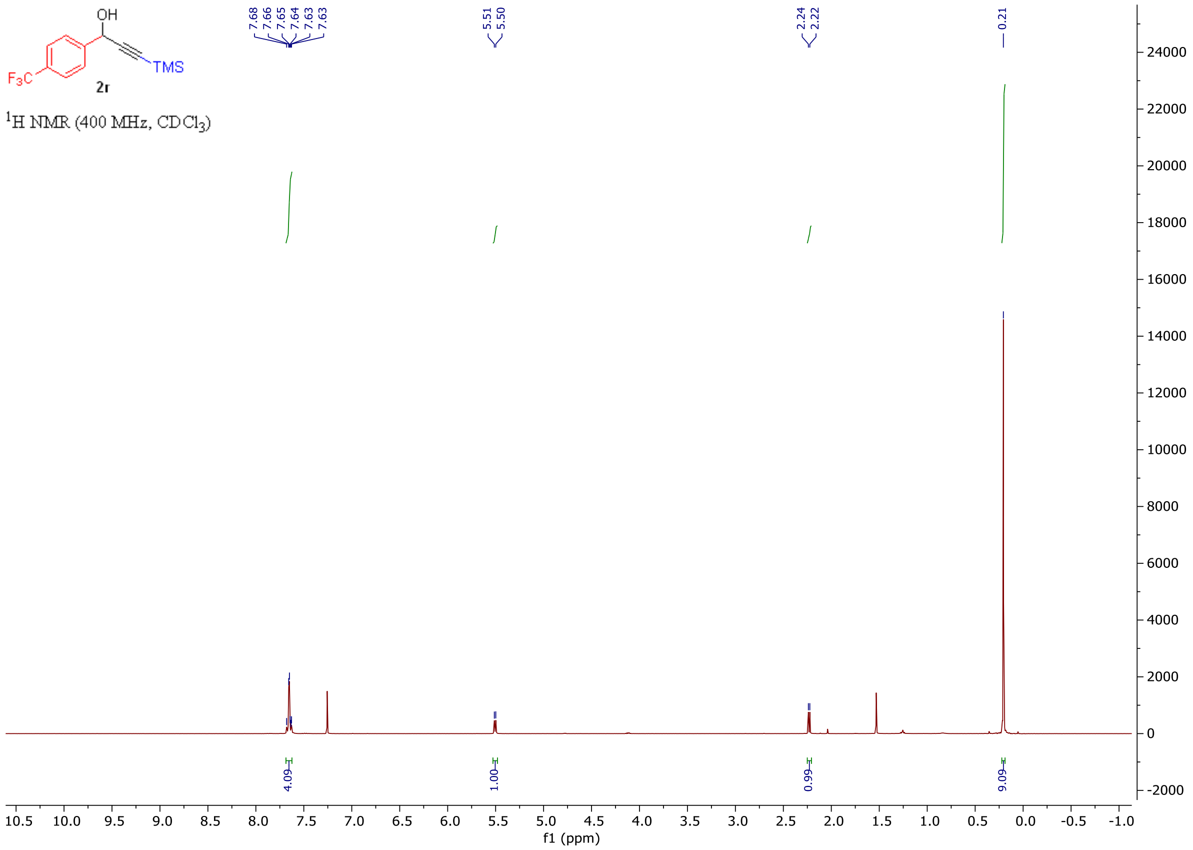
OH
F3C
TMS
2r
1H NMR (400 MHz, CDCl3)
7.68 7.66 7.65 7.64 7.63 7.63
5.51 5.50
2.24 2.22
0.21
4.09
1.00
0.99
9.09
f1 (ppm)

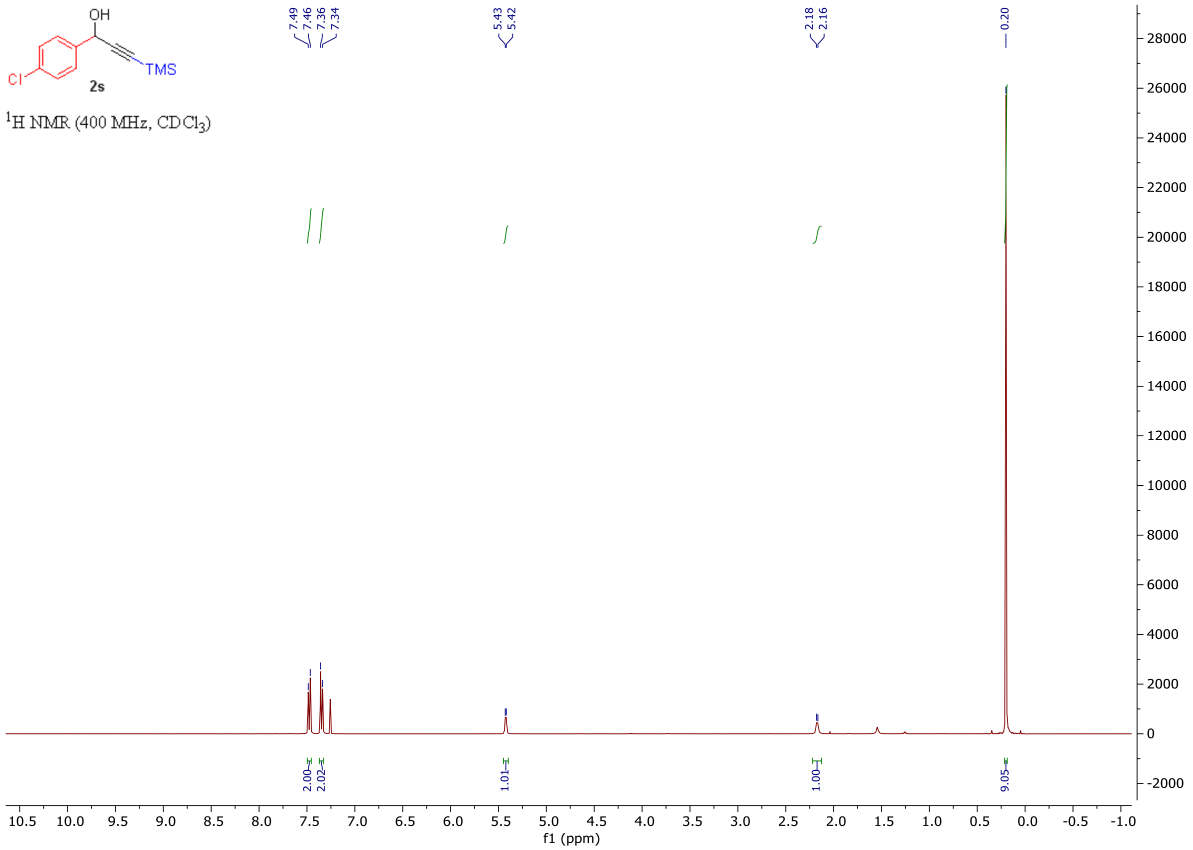
OH
Cl
TMS
2s
1H NMR (400 MHz, CDCl3)
7.49 7.46 7.36 7.34
5.43 5.42
2.18 2.16
0.20
2.00 2.02
1.01
1.00
9.05
f1 (ppm)

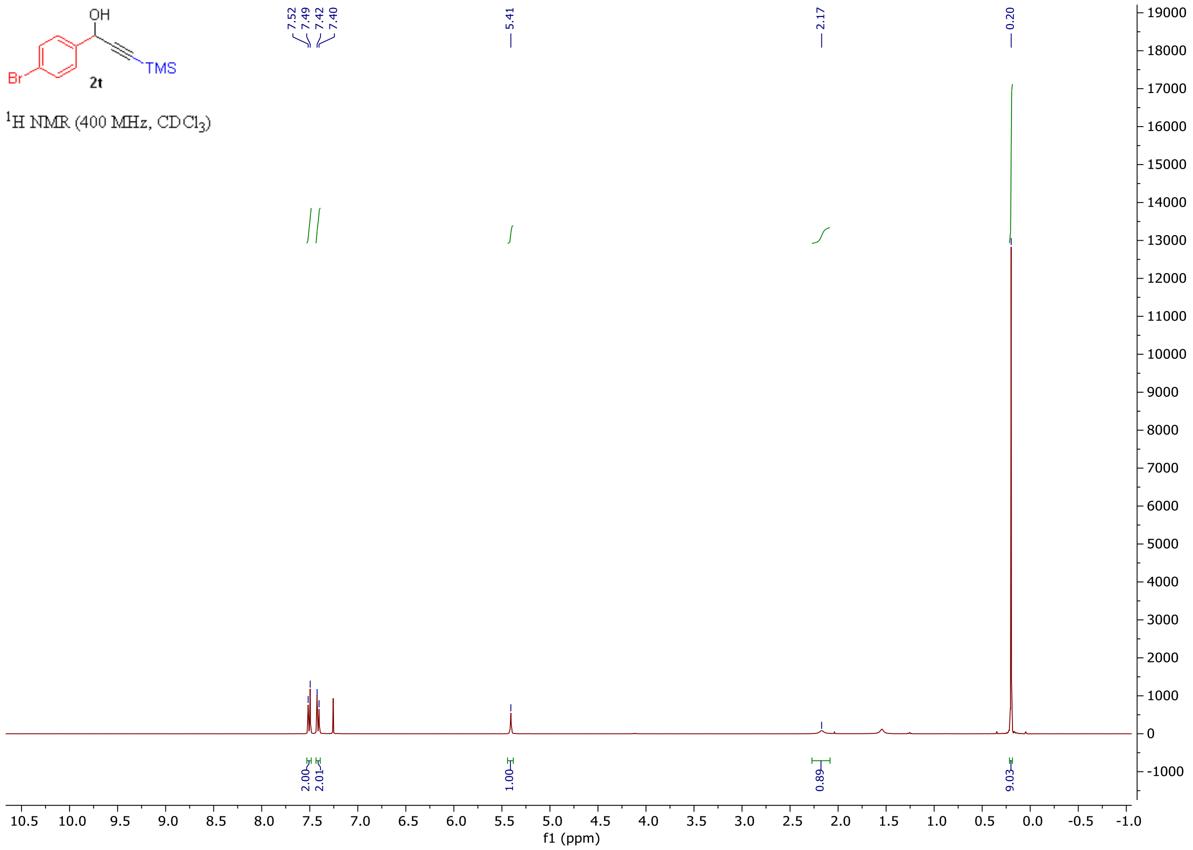

OH
TMS
Br
2t
1H NMR (400 MHz, CDCl3)
7.52
7.49
7.42
7.40
5.41
2.17
0.20
2.00
2.01
1.00
0.89
9.03
f1 (ppm)

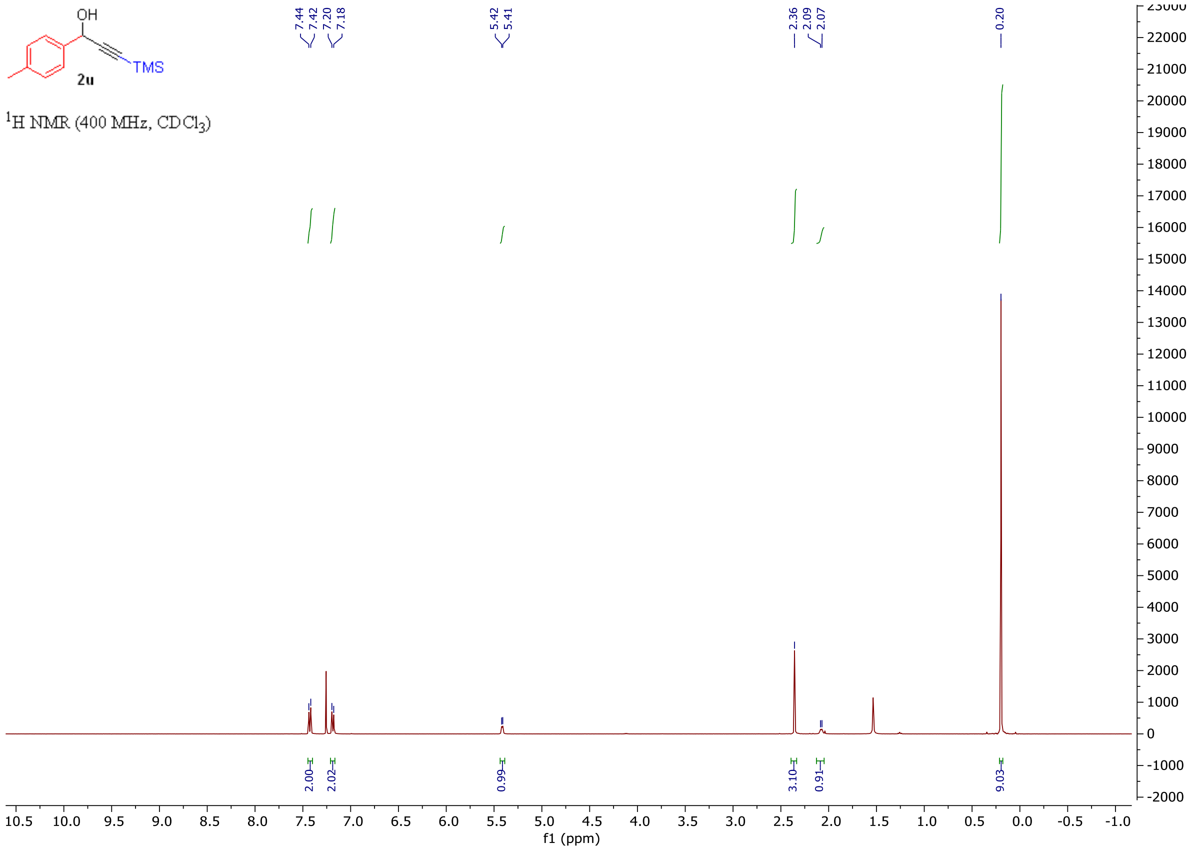

OH
TMS
2u
1H NMR (400 MHz, CDCl3)
7.44
7.42
7.20
7.18
5.42
5.41
2.36
2.09
2.07
0.20
2.00
2.02
0.99
3.10
0.91
9.03
f1 (ppm)

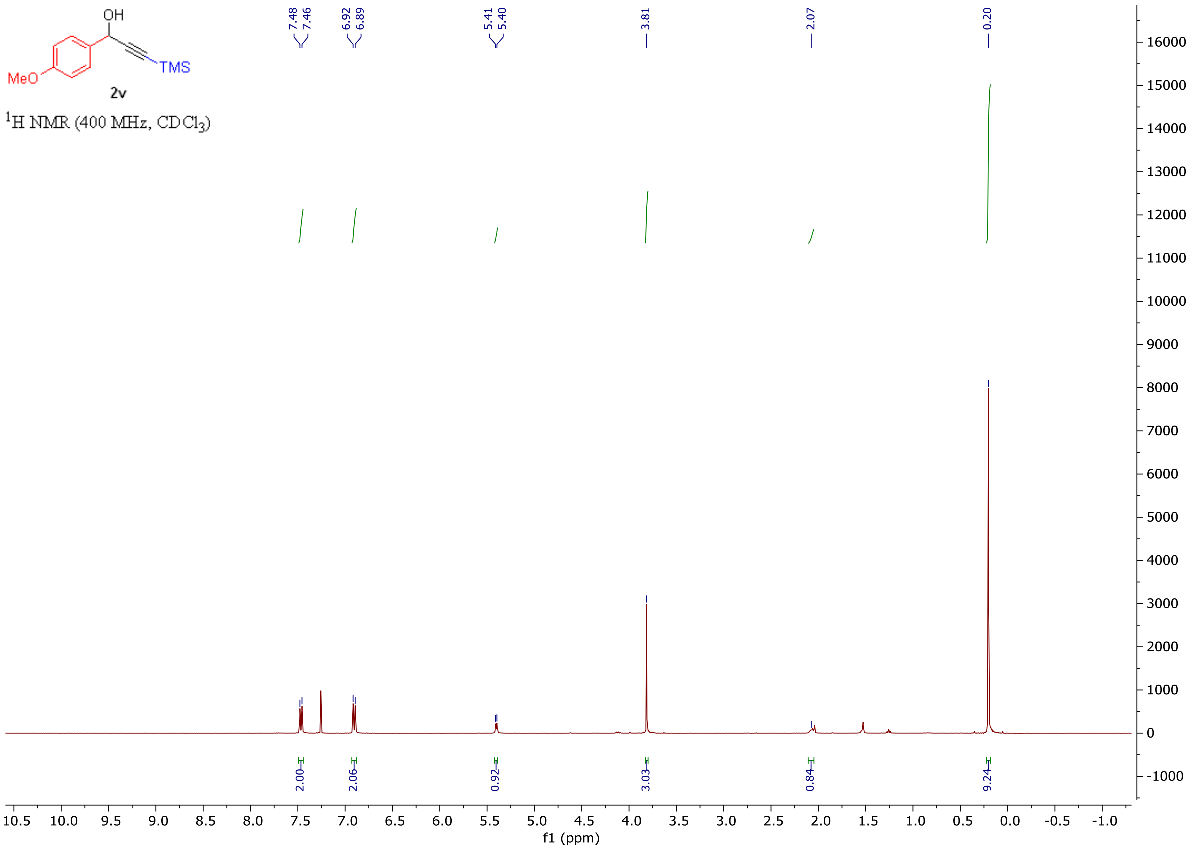
OH
MeO
TMS
2v
^{1}H NMR (400 MHz, CDCl$_3$)
f1 (ppm)

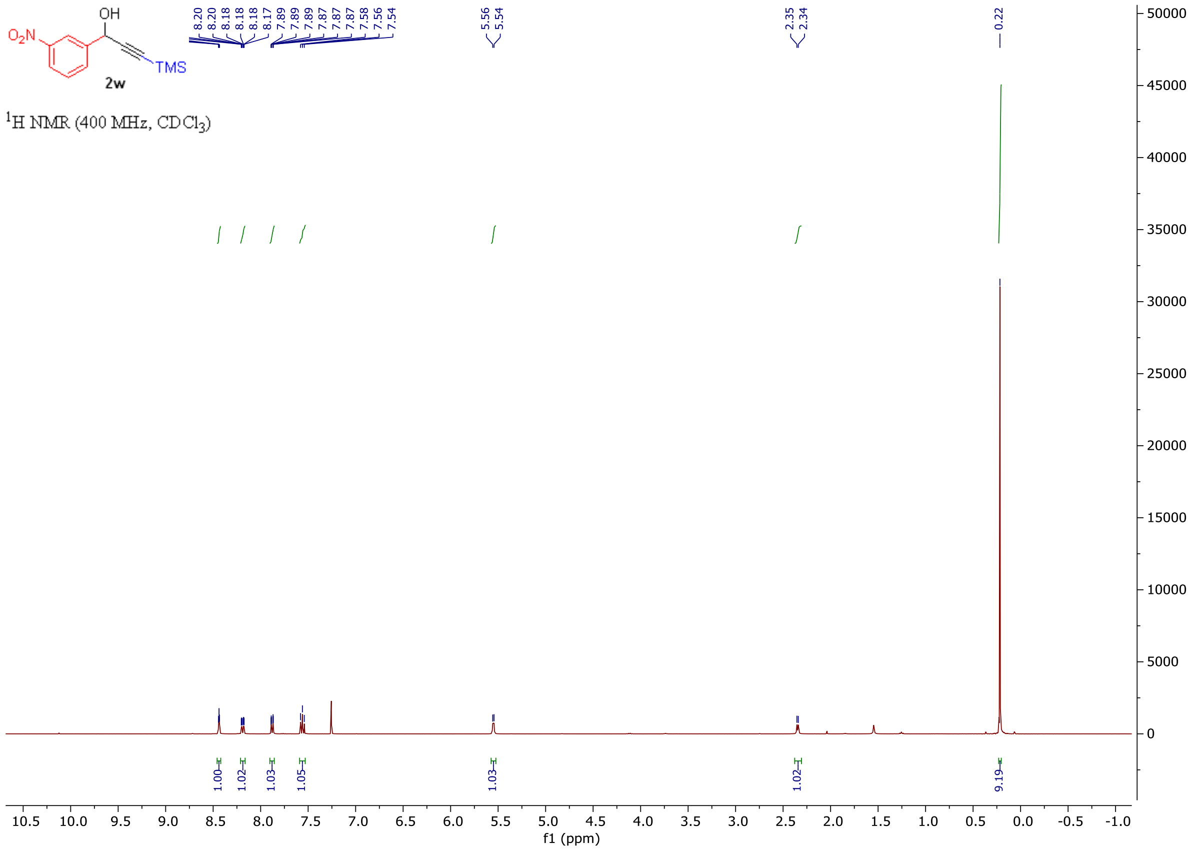
OH
O_2N
TMS
2w
^{1}H NMR (400 MHz, CDCl$_3$)
f1 (ppm)

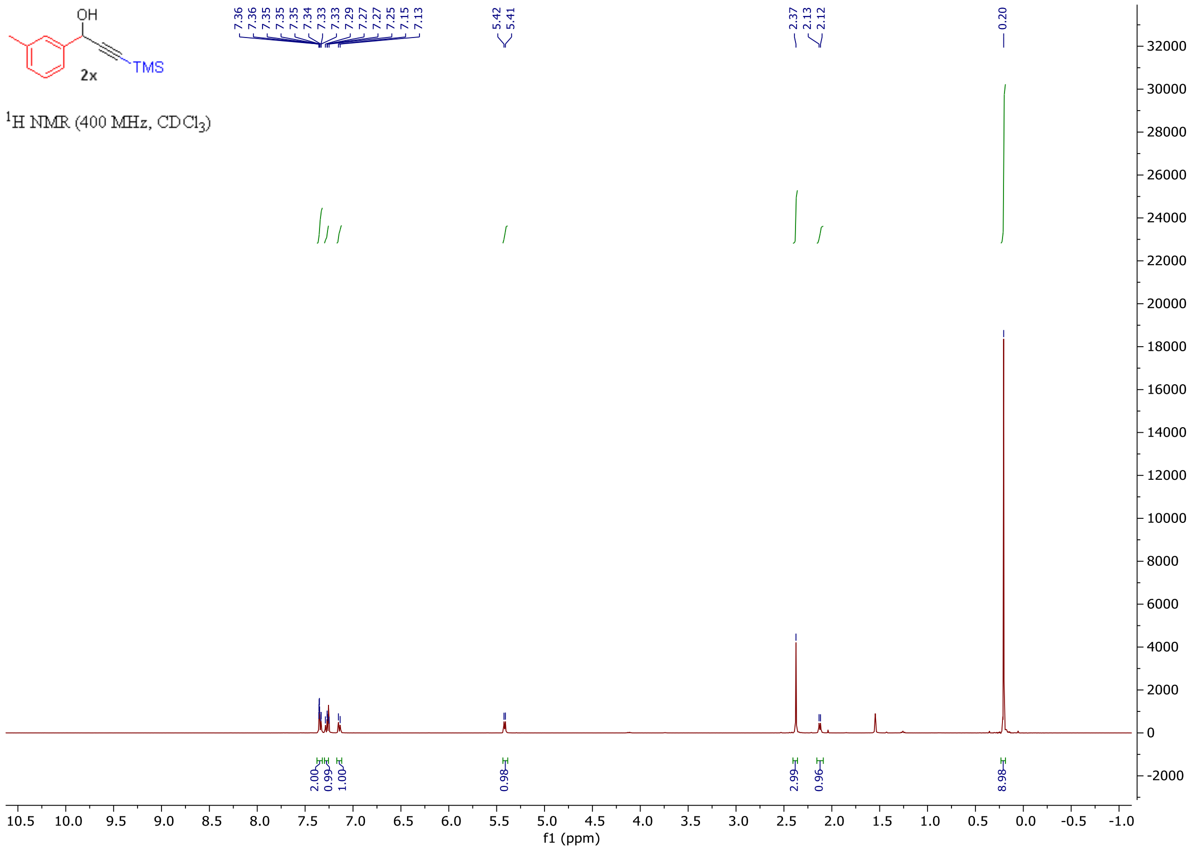

OH
TMS
2x
¹H NMR (400 MHz, CDCl3)
7.36 7.36 7.35 7.35 7.35 7.34 7.33 7.33 7.29 7.27 7.27 7.25 7.15 7.13
5.42 5.41
2.37 2.13 2.12
0.20
2.00 0.99 1.00 0.98 2.99 0.96 8.98
f1 (ppm)

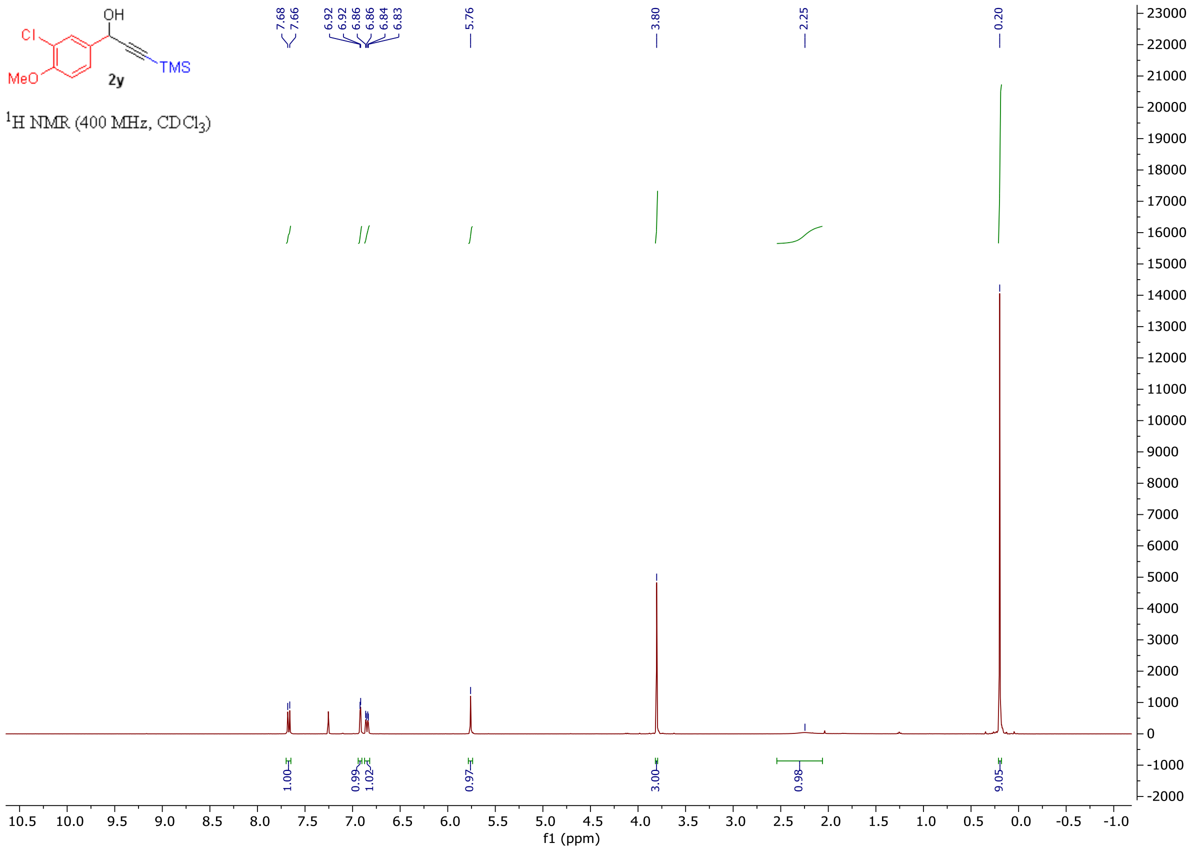

OH
Cl
MeO
TMS
2y
¹H NMR (400 MHz, CDCl3)
7.68 7.66
6.92 6.92 6.86 6.86 6.84 6.83
5.76
3.80
2.25
0.20
1.00 0.99 1.02 0.97 3.00 0.98 9.05
f1 (ppm)

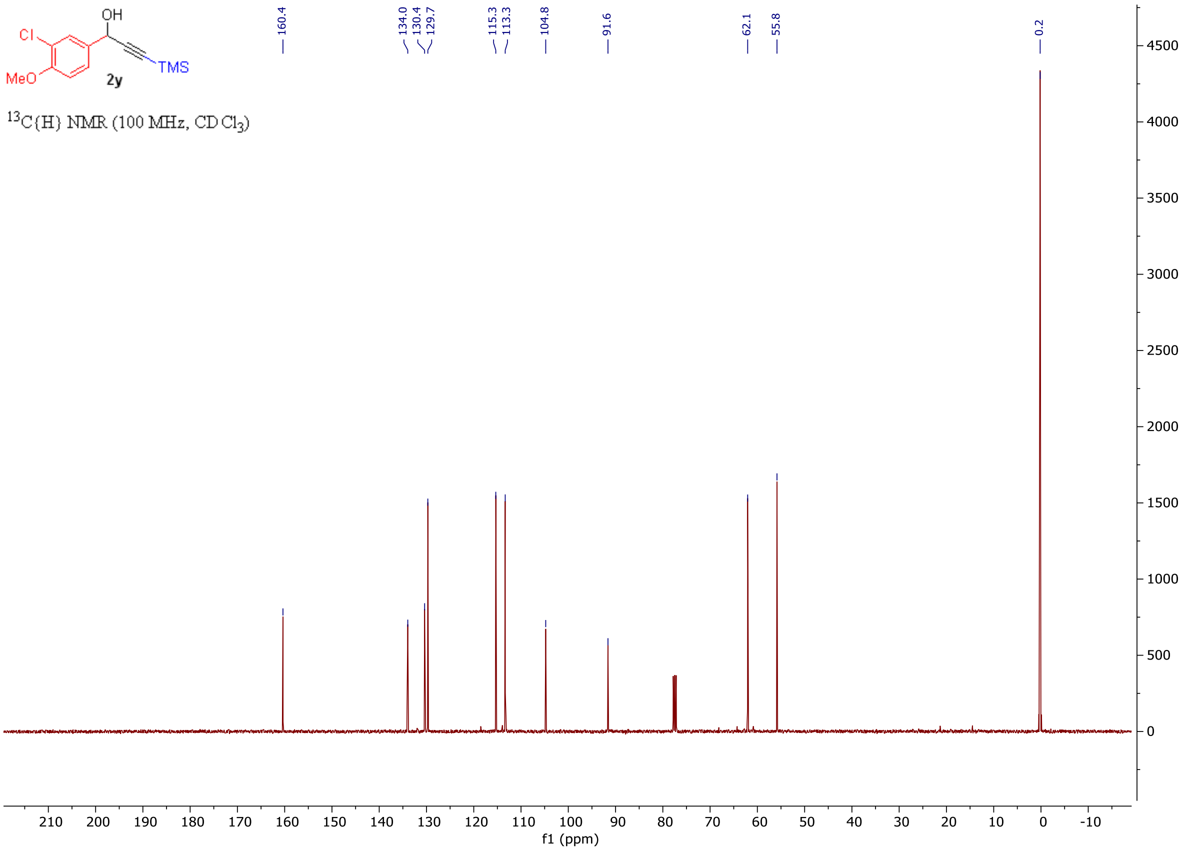

OH
Cl
MeO
TMS
2y
^{13}C{H} NMR (100 MHz, CDCl$_3$)
160.4
134.0
130.4
129.7
115.3
113.3
104.8
91.6
62.1
55.8
0.2
f1 (ppm)

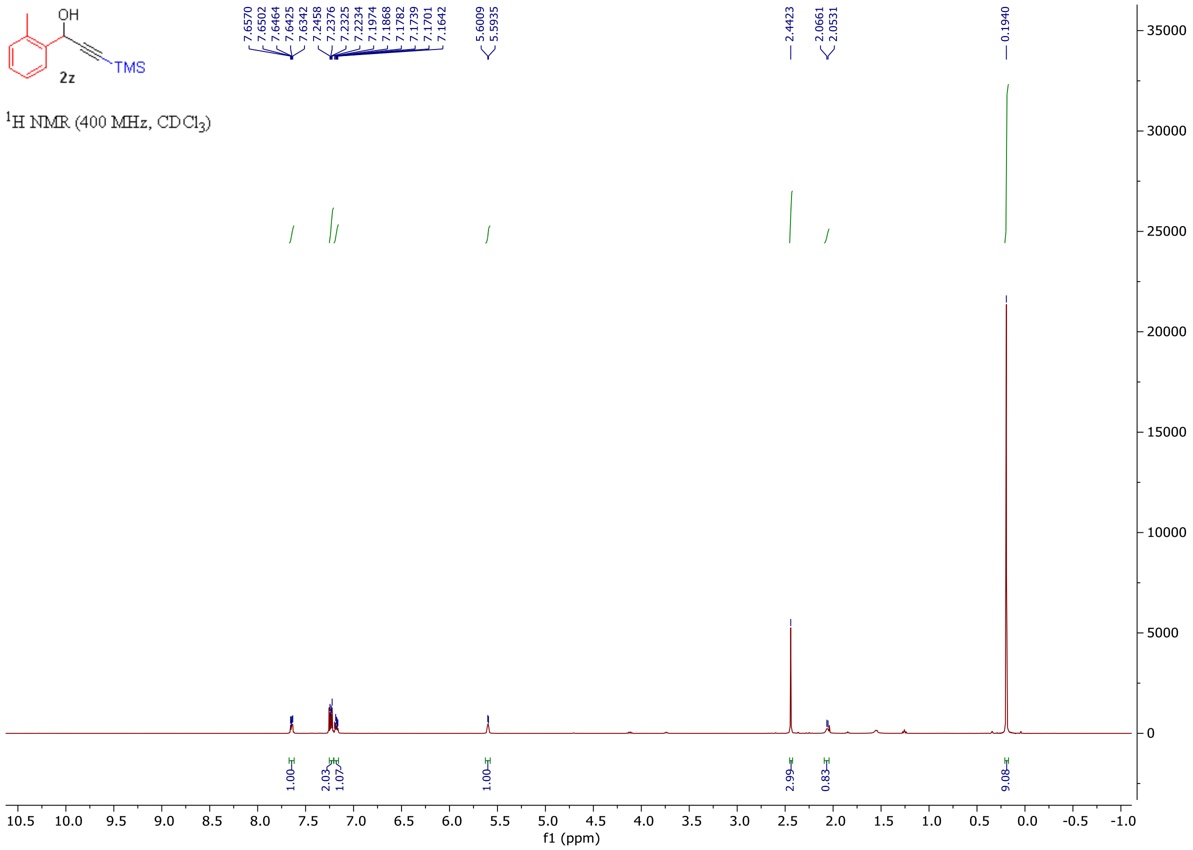

OH
TMS
2z
^{1}H NMR (400 MHz, CDCl$_3$)
7.6570
7.6502
7.6464
7.6425
7.6342
7.2458
7.2376
7.2325
7.2234
7.1974
7.1868
7.1782
7.1739
7.1701
7.1642
5.6009
5.5935
2.4423
2.0661
2.0531
0.1940
1.00
2.03
1.07
1.00
2.99
0.83
9.08
f1 (ppm)

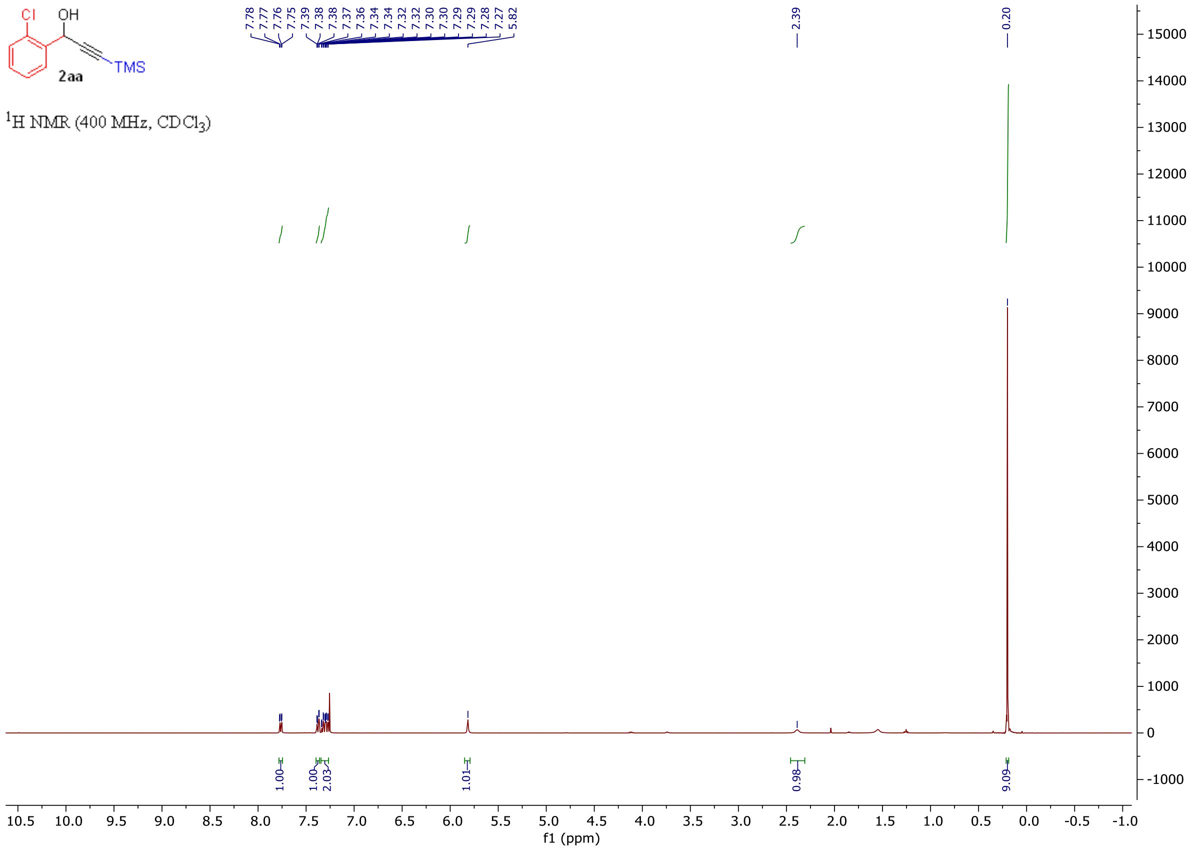
Cl
OH
TMS
2aa
1H NMR (400 MHz, CDCl3)
7.78
7.77
7.76
7.75
7.39
7.38
7.38
7.37
7.36
7.34
7.34
7.32
7.32
7.30
7.30
7.29
7.29
7.28
7.27
5.82
2.39
0.20
1.00
1.00
2.03
1.01
0.98
9.09
f1 (ppm)

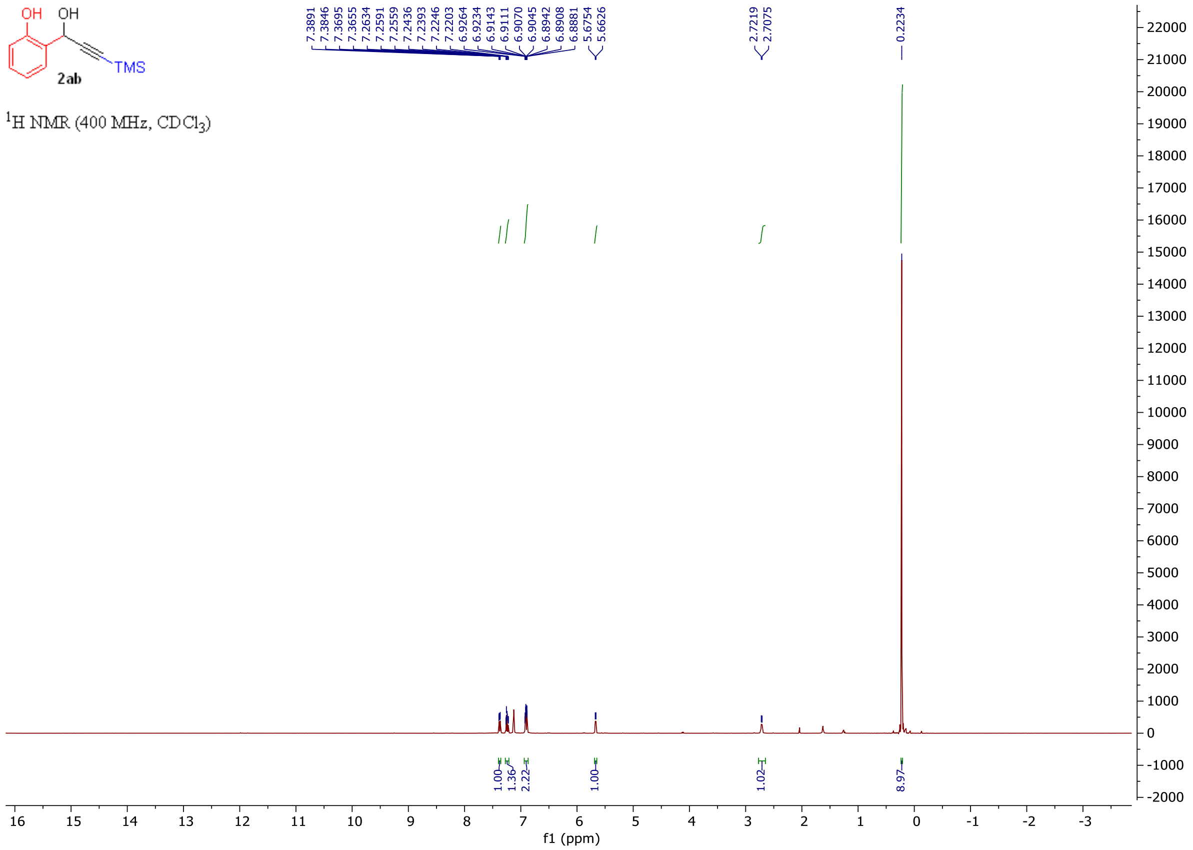
OH
OH
TMS
2ab
1H NMR (400 MHz, CDCl3)
7.3891
7.3846
7.3695
7.3655
7.2634
7.2591
7.2559
7.2436
7.2393
7.2246
7.2203
6.9264
6.9234
6.9143
6.9111
6.9070
6.9045
6.8942
6.8908
6.8881
5.6754
5.6626
2.7219
2.7075
0.2234
1.00
1.36
2.22
1.00
1.02
8.97
f1 (ppm)

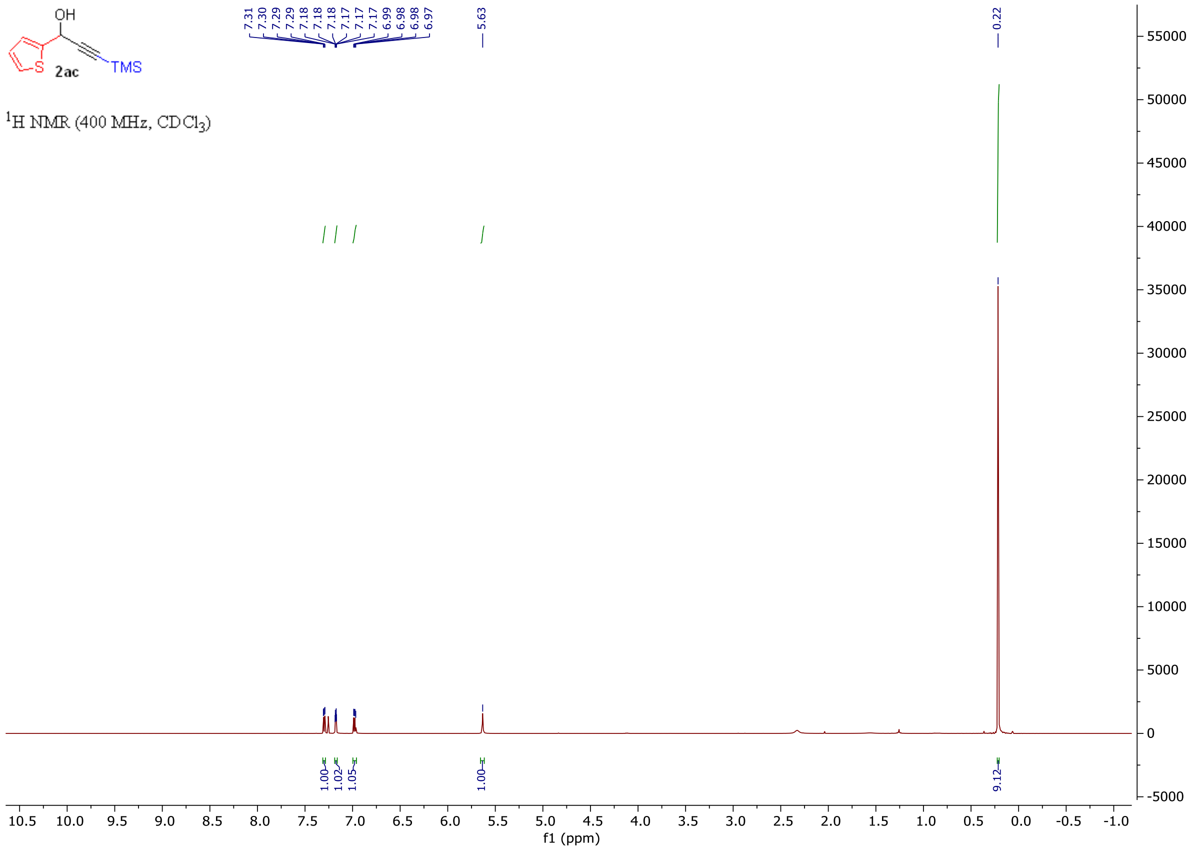
2ac
1H NMR (400 MHz, CDCl3)
f1 (ppm)

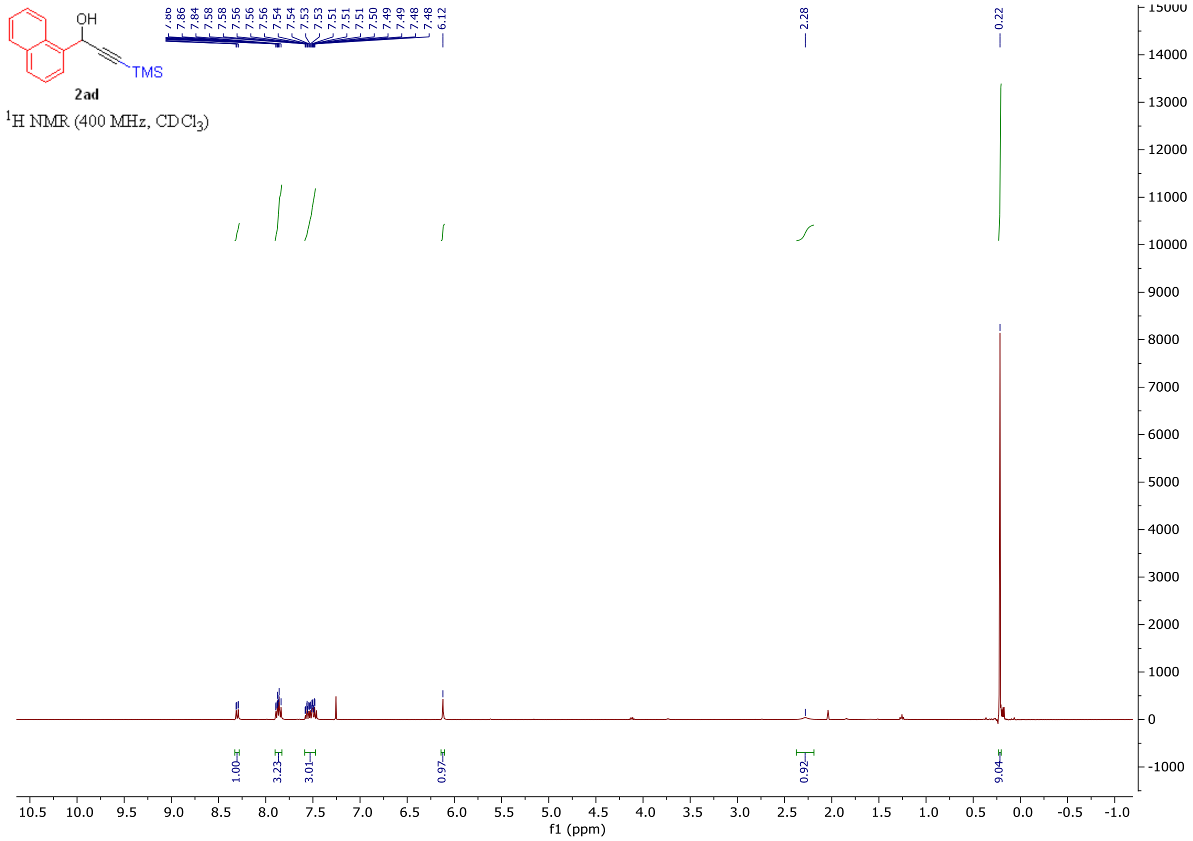
2ad
1H NMR (400 MHz, CDCl3)
f1 (ppm)

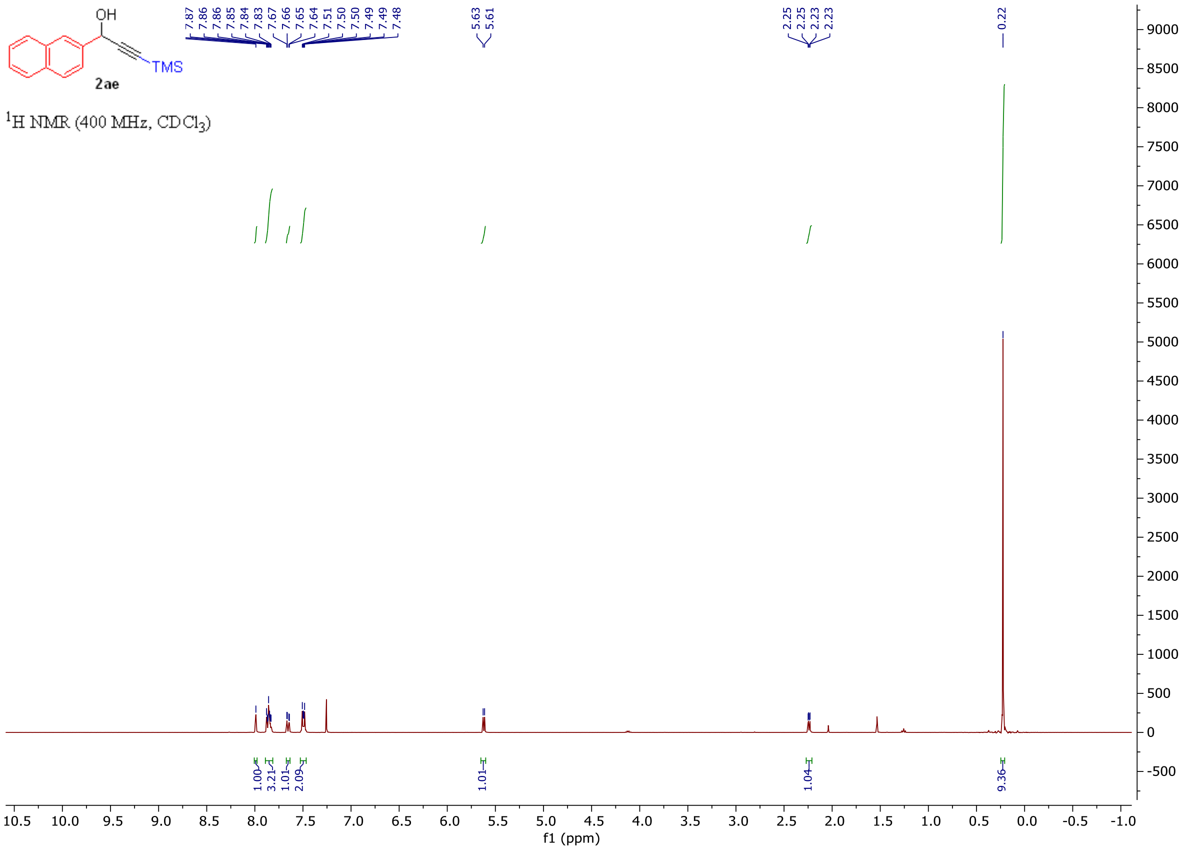
OH
TMS
2ae
^{1}H NMR (400 MHz, CDCl$_3$)
f1 (ppm)

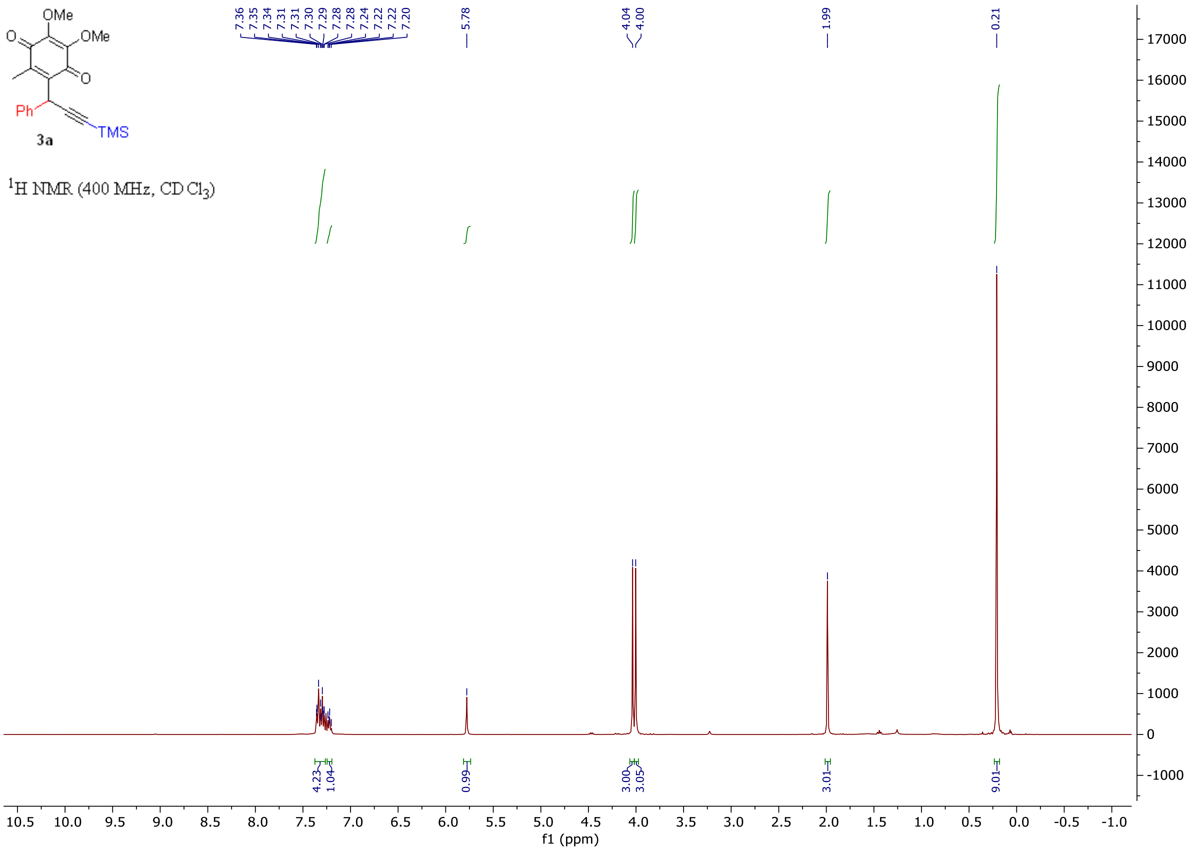
OMe
OMe
Ph
TMS
3a
^{1}H NMR (400 MHz, CDCl$_3$)
f1 (ppm)

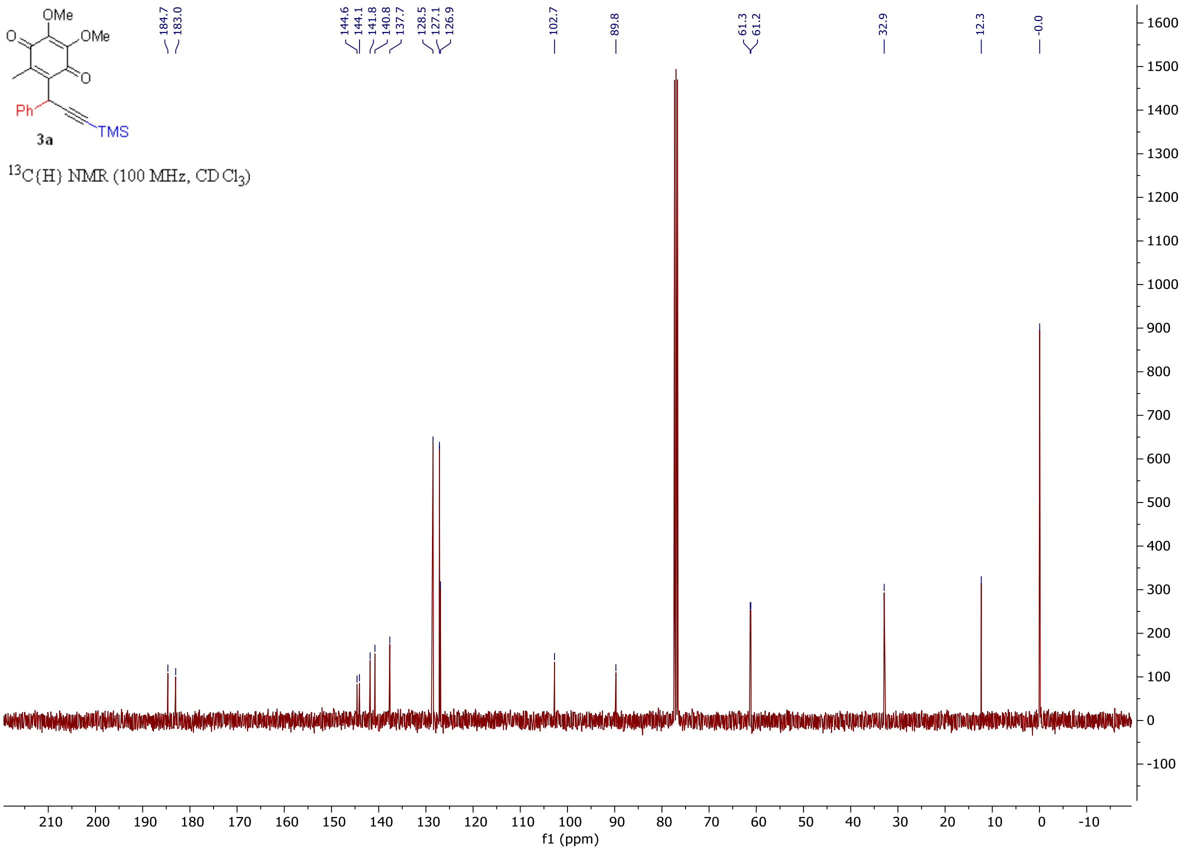
3a
13C{H} NMR (100 MHz, CDCl3)
184.7
183.0
144.6
144.1
141.8
140.8
137.7
128.5
127.1
126.9
102.7
89.8
61.3
61.2
32.9
12.3
-0.0
f1 (ppm)

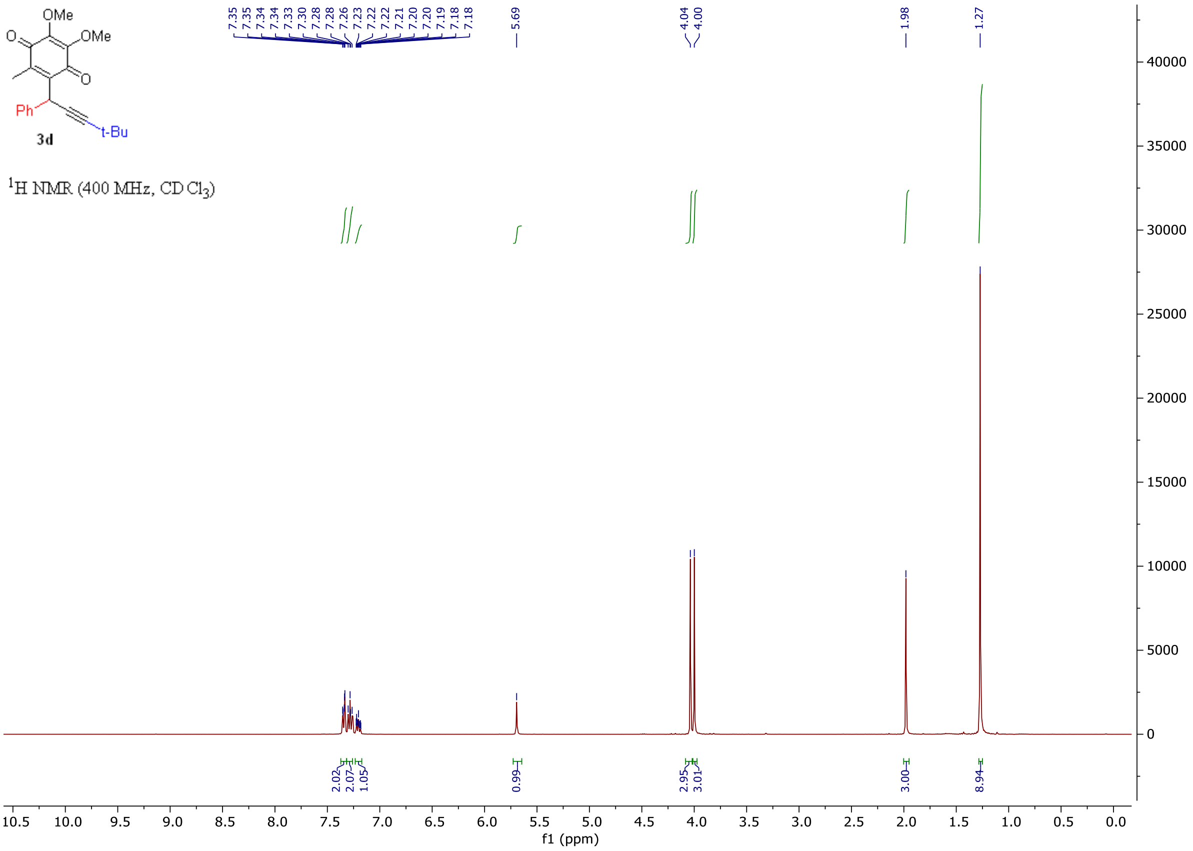
3d
1H NMR (400 MHz, CDCl3)
7.35
7.35
7.34
7.34
7.33
7.30
7.28
7.28
7.26
7.23
7.22
7.22
7.21
7.20
7.20
7.19
7.18
7.18
5.69
4.04
4.00
1.98
1.27
2.02
2.07
1.05
0.99
2.95
3.01
3.00
8.94
f1 (ppm)

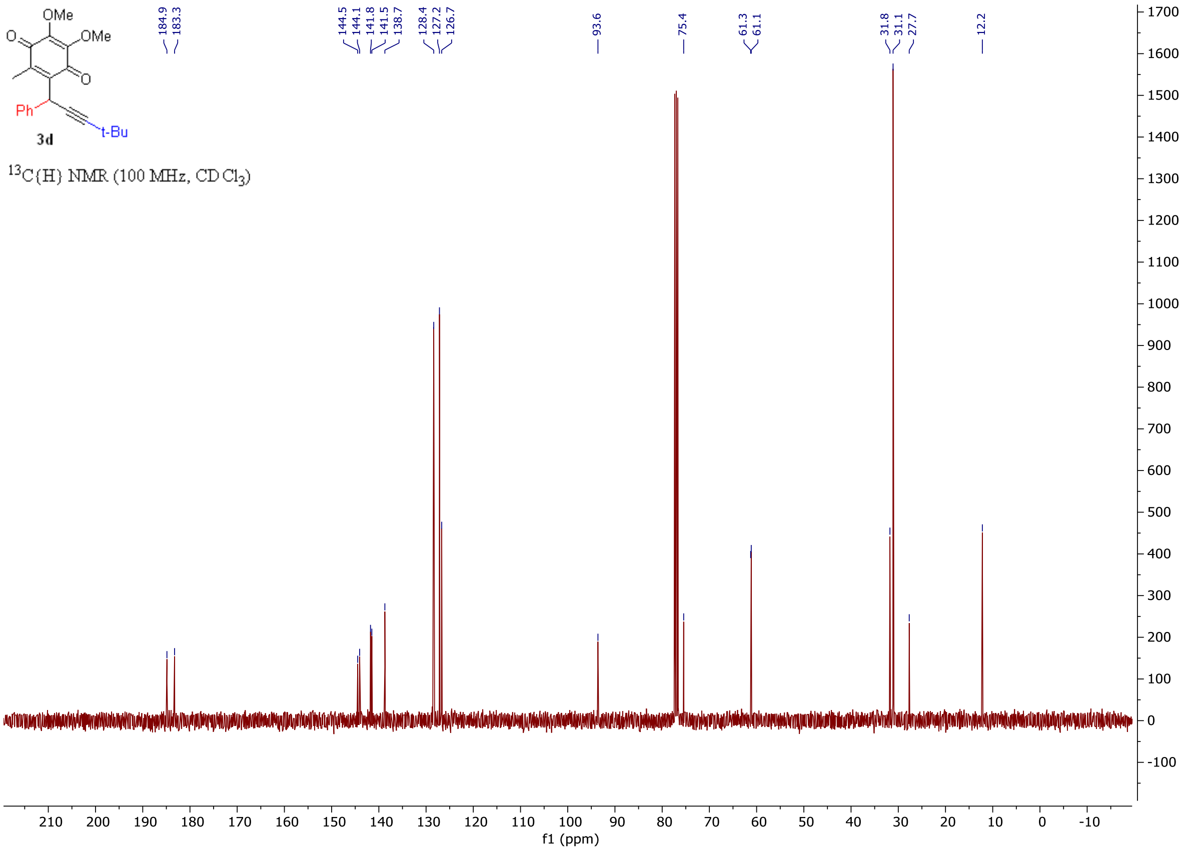

OMe
O
OMe
O
Ph
t-Bu
3d
^{13}C{H} NMR (100 MHz, CDCl$_3$)
184.9
183.3
144.5
144.1
141.8
141.5
138.7
128.4
127.2
126.7
93.6
75.4
61.3
61.1
31.8
31.1
27.7
12.2
f1 (ppm)

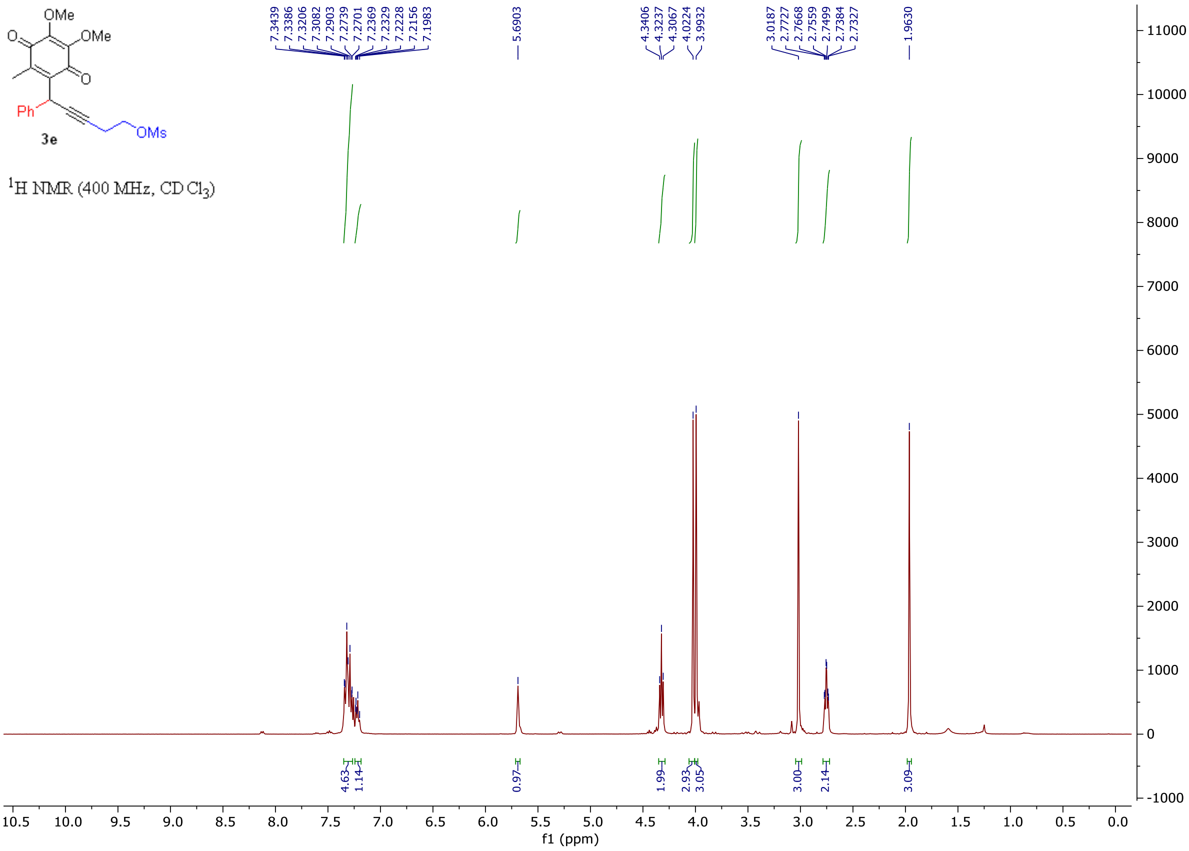

OMe
O
OMe
O
Ph
OMs
3e
^{1}H NMR (400 MHz, CDCl$_3$)
7.3439
7.3386
7.3206
7.3082
7.2903
7.2739
7.2701
7.2369
7.2329
7.2228
7.2156
7.1983
5.6903
4.3406
4.3237
4.3067
4.0224
3.9932
3.0187
2.7727
2.7668
2.7559
2.7499
2.7384
2.7327
1.9630
4.63
1.14
0.97
1.99
2.93
3.05
3.00
2.14
3.09
f1 (ppm)

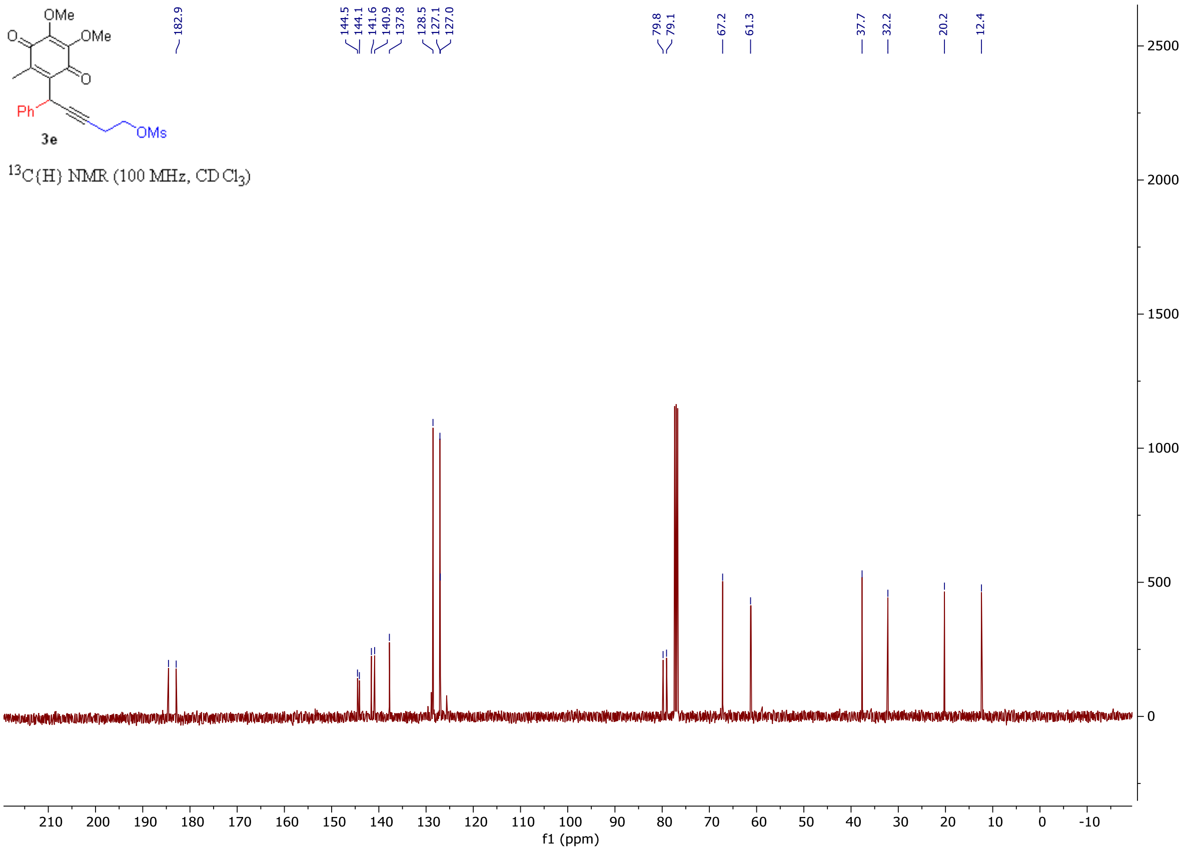

OMe
OMe
O
O
Ph
OMs
3e
$^{13}C\{H\}$ NMR (100 MHz, $CDCl_3$)
182.9
144.5
144.1
141.6
140.9
137.8
128.5
127.1
127.0
79.8
79.1
67.2
61.3
37.7
32.2
20.2
12.4
f1 (ppm)

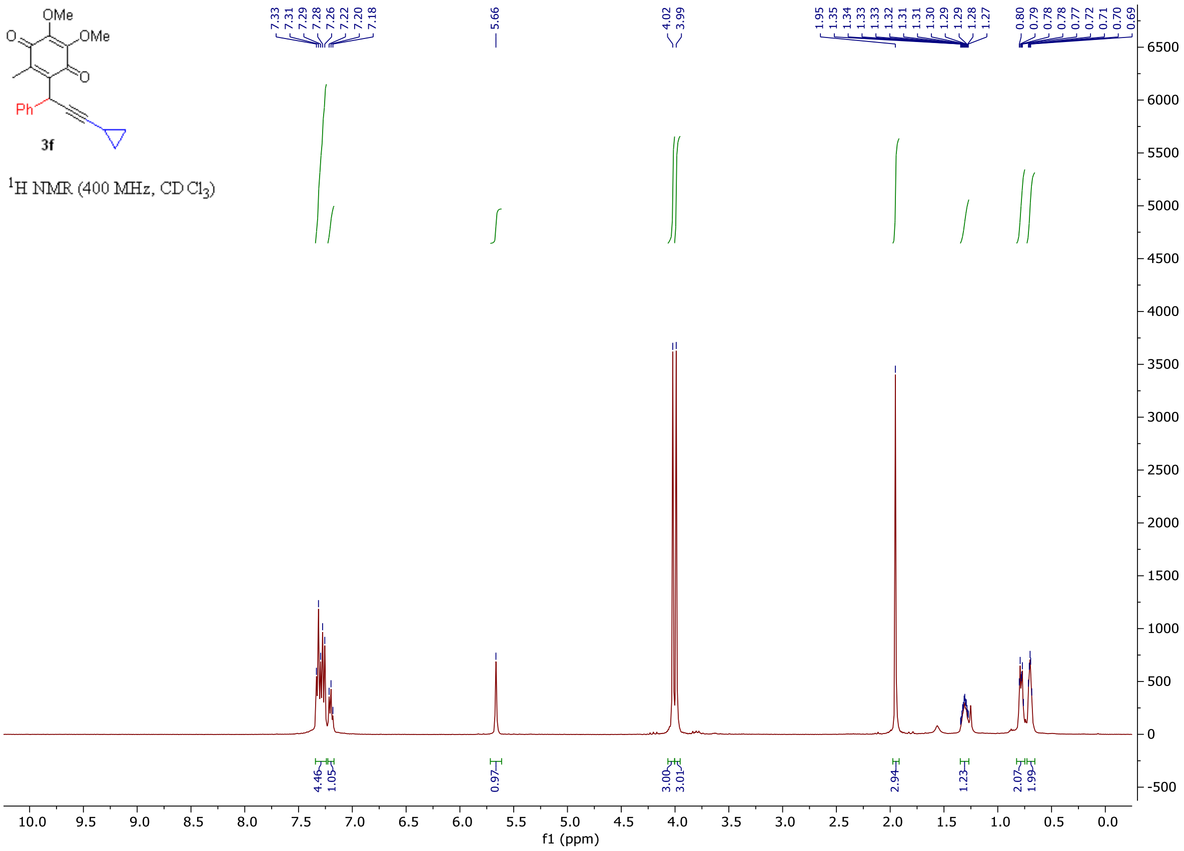

OMe
OMe
O
O
Ph
3f
^{1}H NMR (400 MHz, $CDCl_3$)
7.33
7.31
7.29
7.28
7.26
7.22
7.20
7.18
5.66
4.02
3.99
1.95
1.35
1.34
1.33
1.33
1.32
1.31
1.31
1.30
1.29
1.29
1.28
1.27
0.80
0.79
0.78
0.78
0.77
0.72
0.71
0.70
0.69
4.46
1.05
0.97
3.00
3.01
2.94
1.23
2.07
1.99
f1 (ppm)

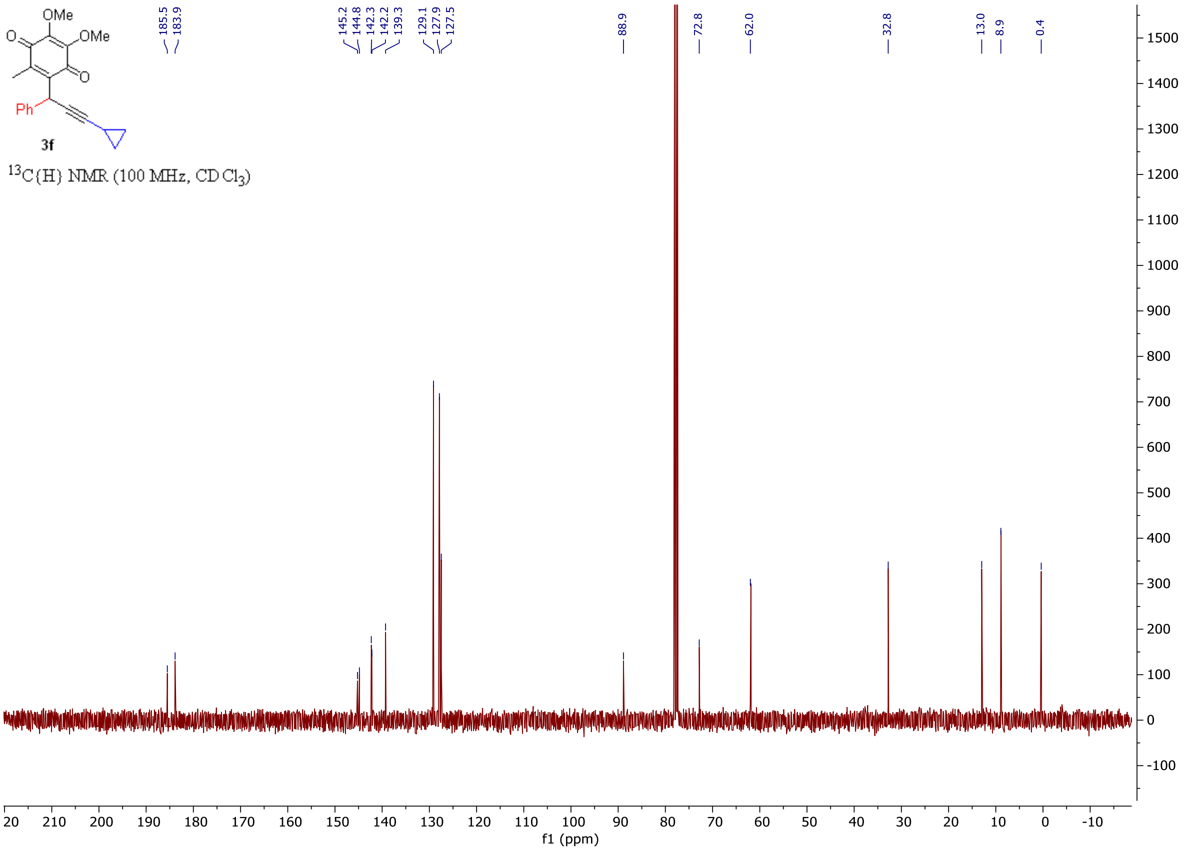
OMe
OMe
O
O
Ph
3f
13C{H} NMR (100 MHz, CDCl3)
185.5
183.9
145.2
144.8
142.3
142.2
139.3
129.1
127.9
127.5
88.9
72.8
62.0
32.8
13.0
8.9
0.4
f1 (ppm)

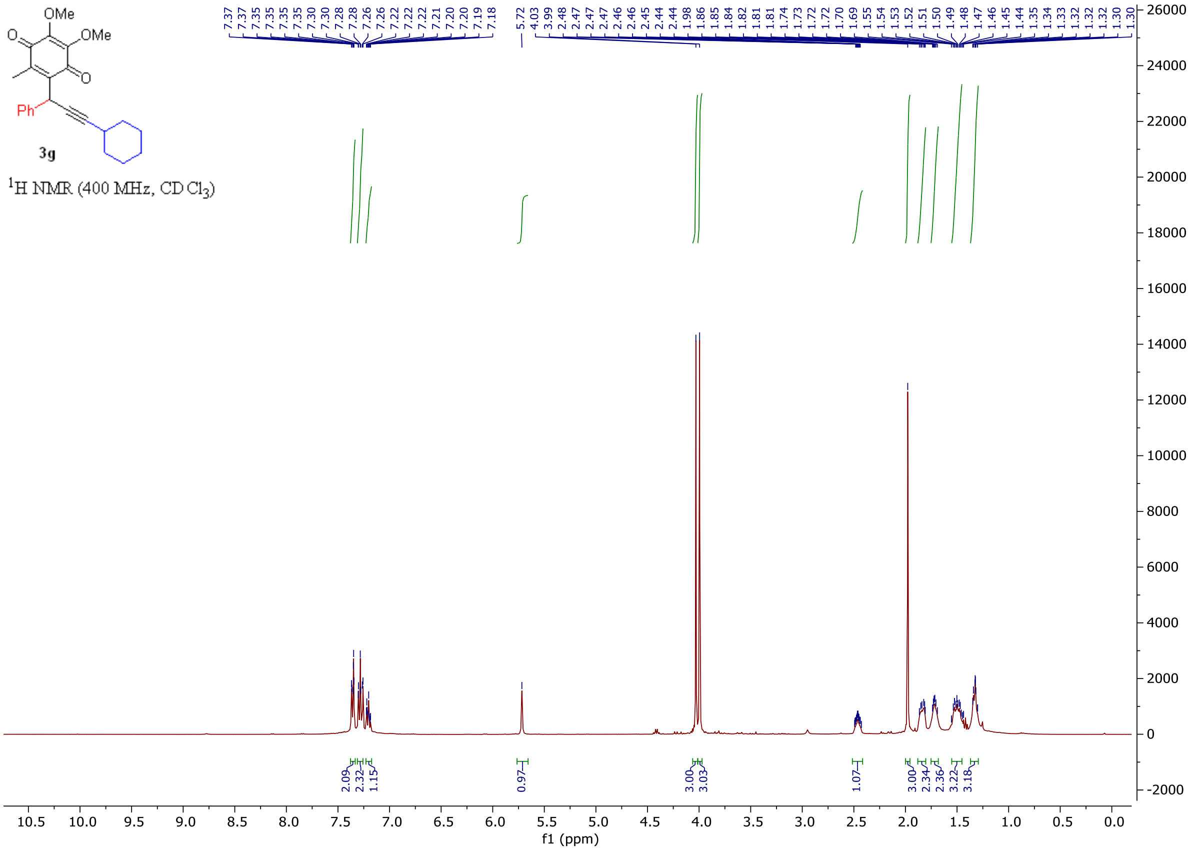
OMe
OMe
O
O
Ph
3g
1H NMR (400 MHz, CDCl3)
7.37
7.37
7.35
7.35
7.35
7.30
7.28
7.26
7.26
7.22
7.22
7.21
7.20
7.19
7.18
5.72
4.03
3.99
2.48
2.47
2.47
2.46
2.45
2.44
1.98
1.86
1.85
1.84
1.82
1.81
1.74
1.73
1.72
1.70
1.69
1.55
1.54
1.53
1.52
1.51
1.50
1.49
1.48
1.47
1.46
1.45
1.44
1.35
1.34
1.33
1.32
1.32
1.30
1.30
2.09
2.32
1.15
0.97
3.00
3.03
1.07
3.00
2.34
2.36
3.22
3.18
f1 (ppm)

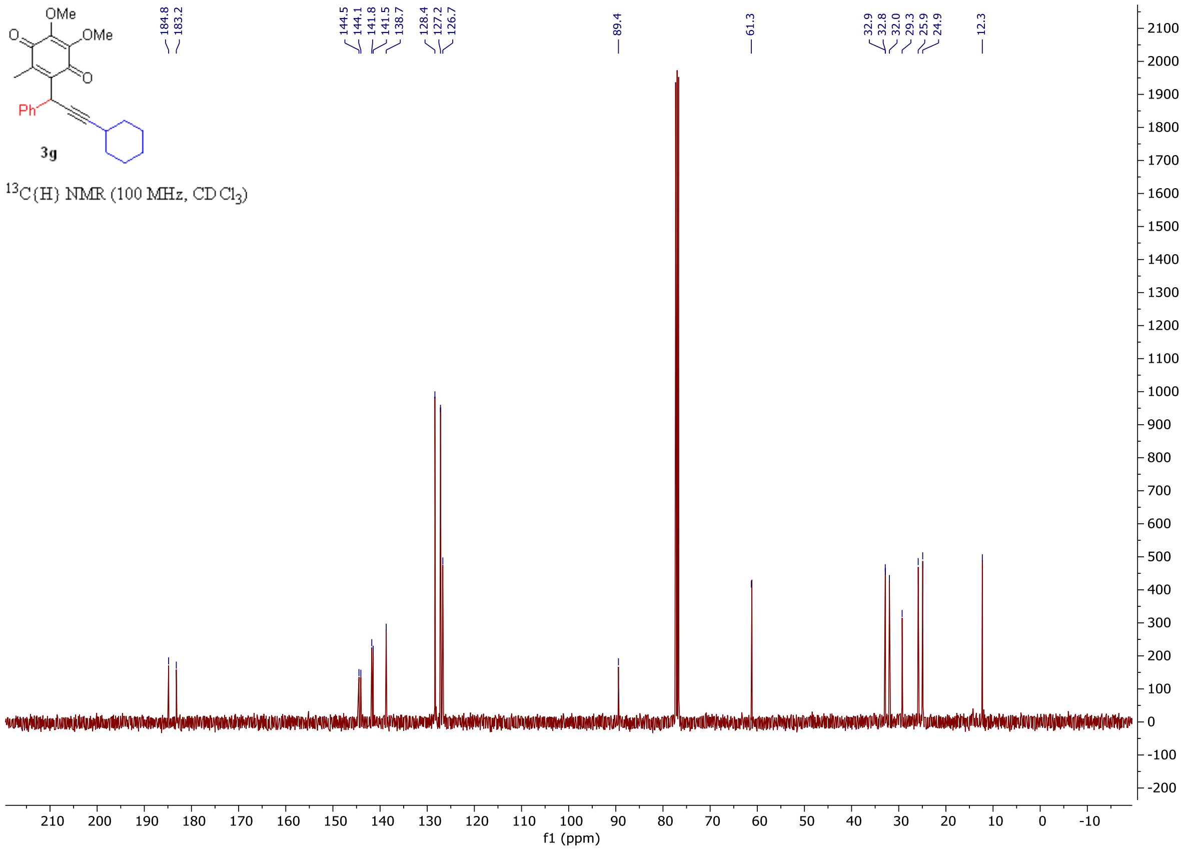

OMe
OMe
Ph
3g
^{13}C{H} NMR (100 MHz, CDCl$_3$)
184.8
183.2
144.5
144.1
141.8
141.5
138.7
128.4
127.2
126.7
89.4
61.3
32.9
32.8
32.0
29.3
25.9
24.9
12.3
f1 (ppm)

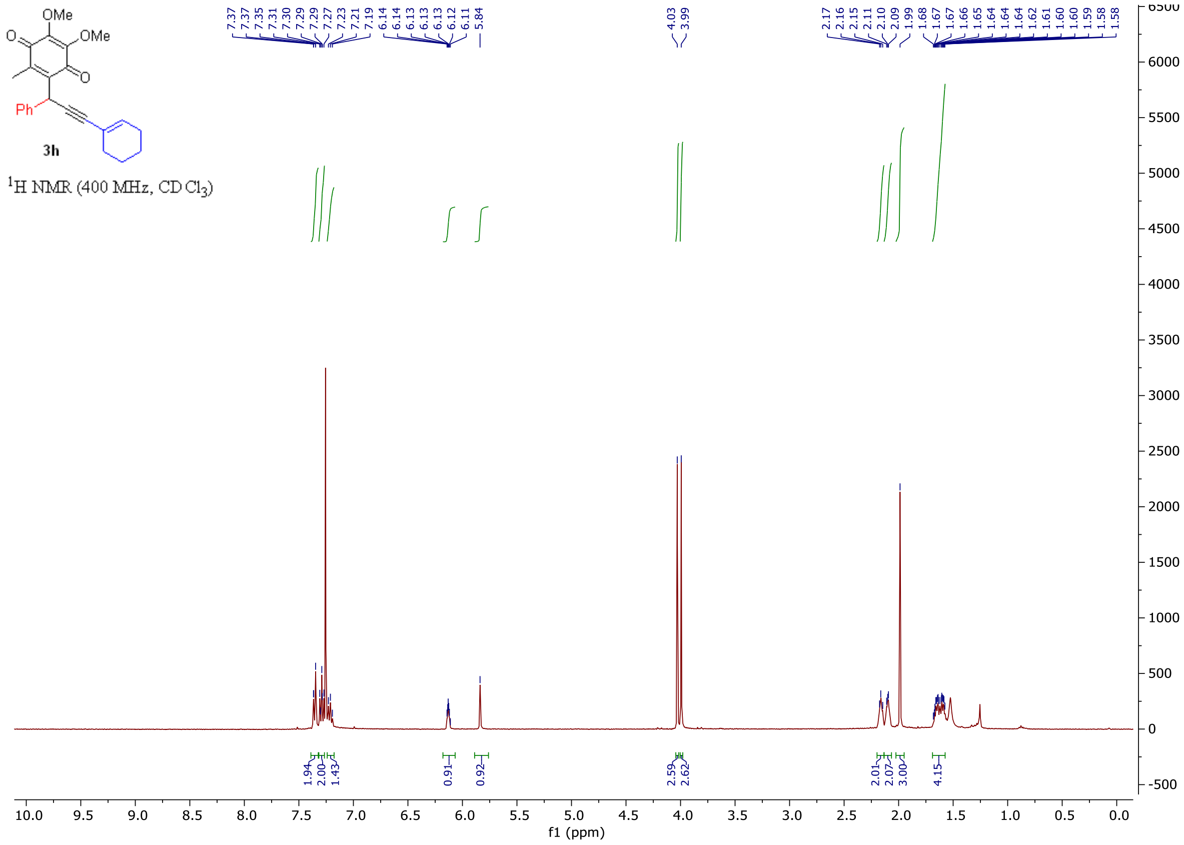

OMe
OMe
Ph
3h
^{1}H NMR (400 MHz, CDCl$_3$)
7.37
7.37
7.35
7.31
7.30
7.29
7.29
7.27
7.23
7.21
7.19
6.14
6.13
6.13
6.13
6.12
6.11
5.84
4.03
3.99
2.17
2.16
2.15
2.11
2.10
2.09
1.99
1.68
1.67
1.66
1.65
1.64
1.64
1.62
1.61
1.60
1.59
1.58
1.58
1.94
2.00
1.43
0.91
0.92
2.59
2.62
2.01
2.07
3.00
4.15
f1 (ppm)

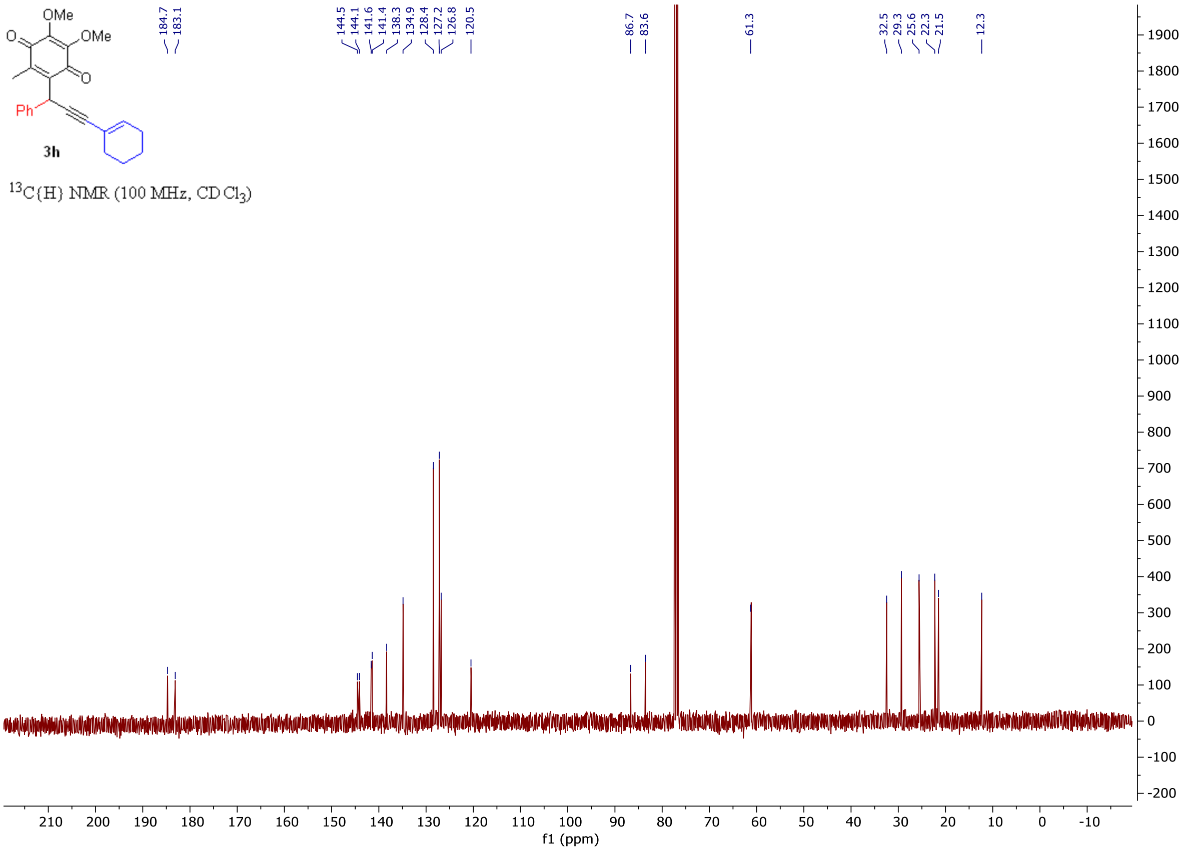
OMe
OMe
Ph
3h
13C{H} NMR (100 MHz, CDCl3)
184.7
183.1
144.5
144.1
141.6
141.4
138.3
134.9
128.4
127.2
126.8
120.5
86.7
83.6
61.3
32.5
29.3
25.6
22.3
21.5
12.3
f1 (ppm)

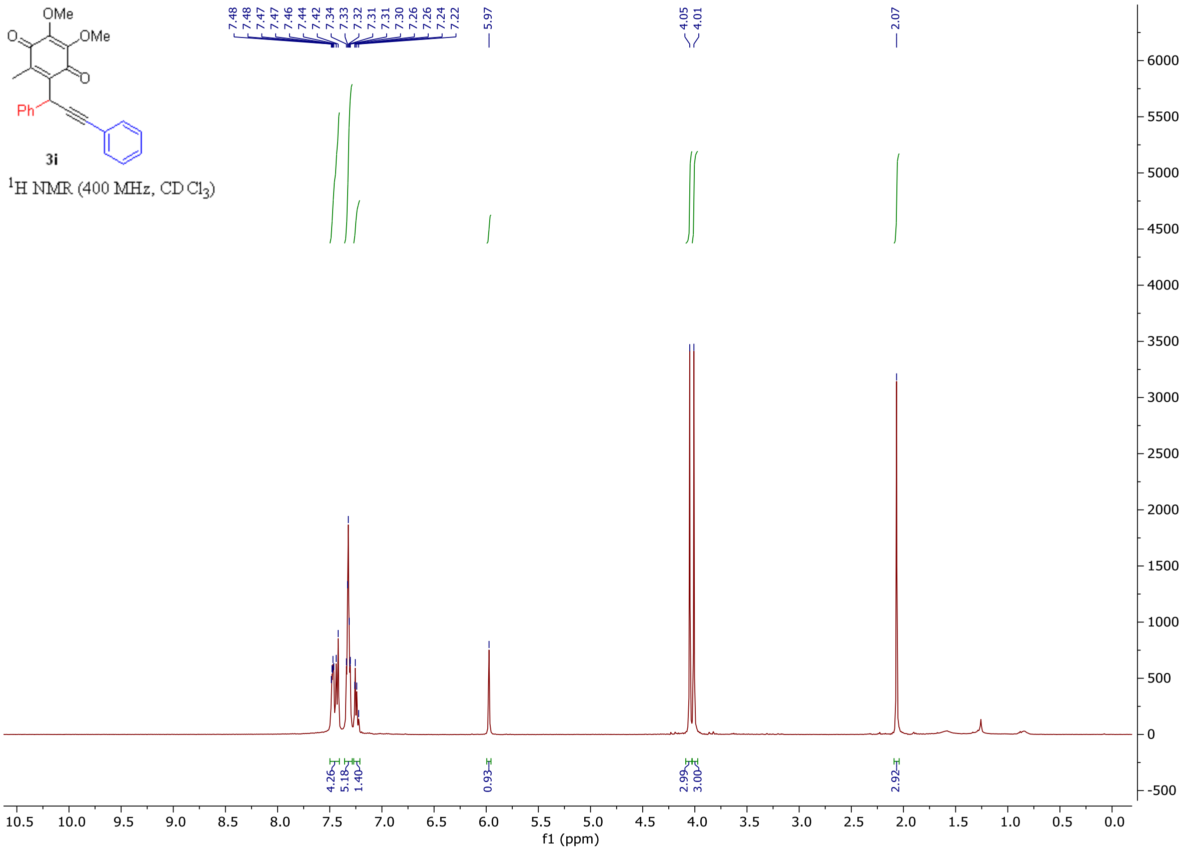
OMe
OMe
Ph
3i
1H NMR (400 MHz, CDCl3)
7.48
7.48
7.47
7.46
7.44
7.42
7.34
7.33
7.32
7.31
7.31
7.30
7.26
7.24
7.22
5.97
4.05
4.01
2.07
4.26
5.18
1.40
0.93
2.99
3.00
2.92
f1 (ppm)

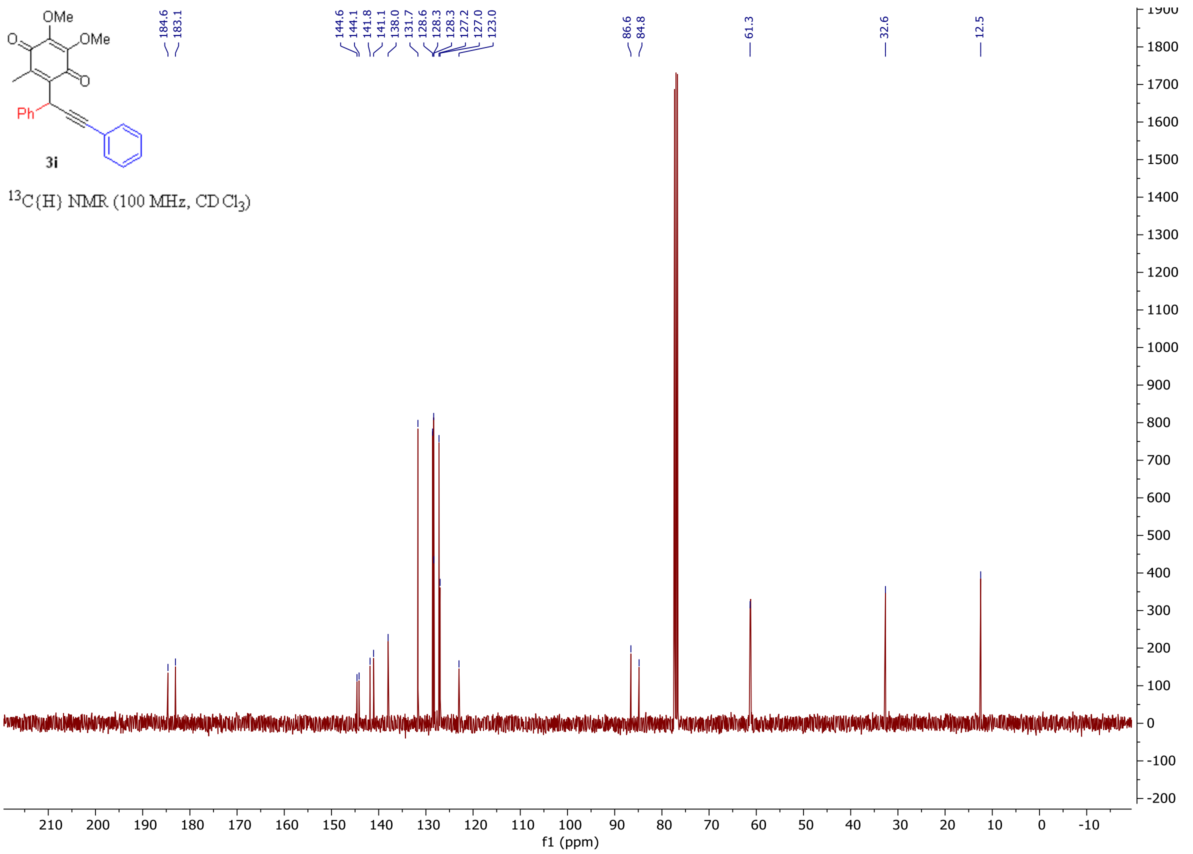
OMe
OMe
Ph
3i
$^{13}C\{H\}$ NMR (100 MHz, $CDCl_3$)
184.6
183.1
144.6
144.1
141.8
141.1
138.0
131.7
128.6
128.3
128.3
127.2
127.0
123.0
86.6
84.8
61.3
32.6
12.5
f1 (ppm)

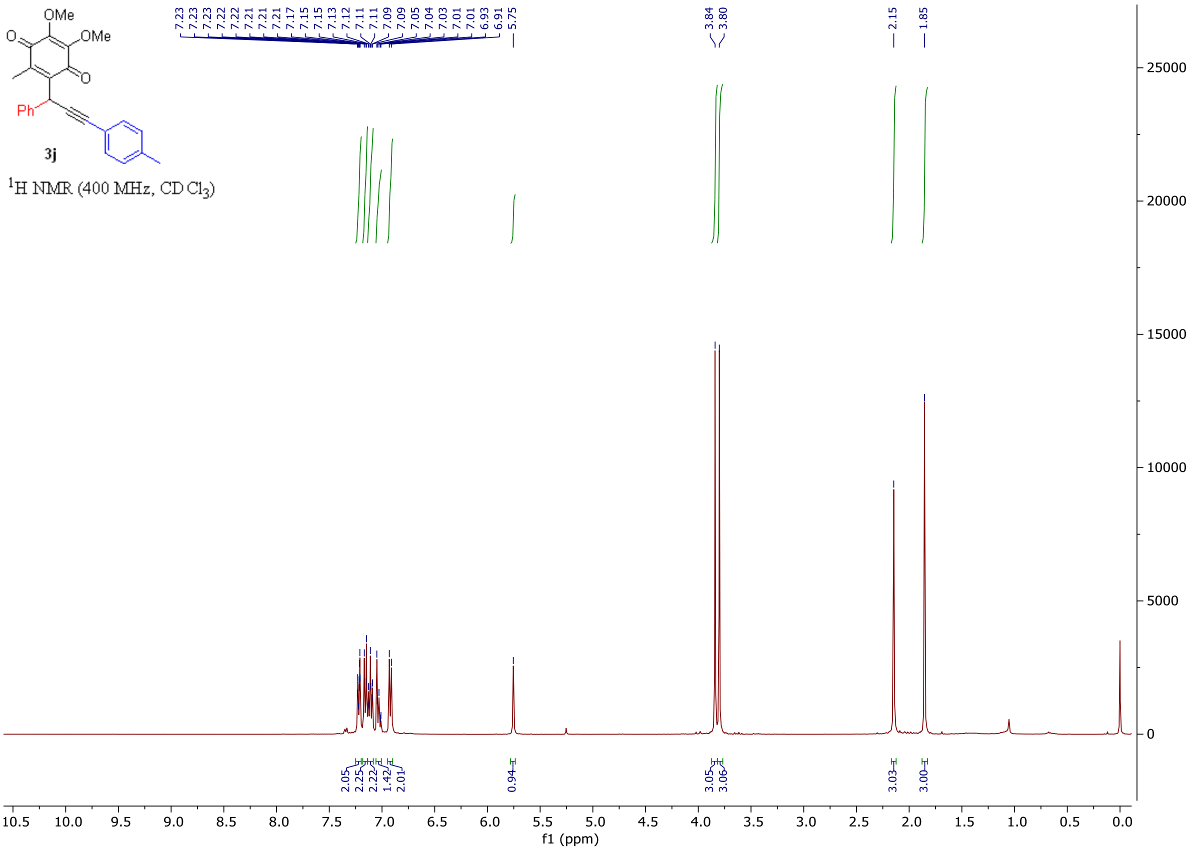
OMe
OMe
Ph
3j
^{1}H NMR (400 MHz, $CDCl_3$)
7.23
7.23
7.23
7.22
7.22
7.21
7.21
7.17
7.15
7.15
7.13
7.12
7.11
7.11
7.09
7.05
7.04
7.03
7.01
7.01
6.93
6.91
5.75
3.84
3.80
2.15
1.85
2.05
2.25
2.22
1.42
2.01
0.94
3.05
3.06
3.03
3.00
f1 (ppm)

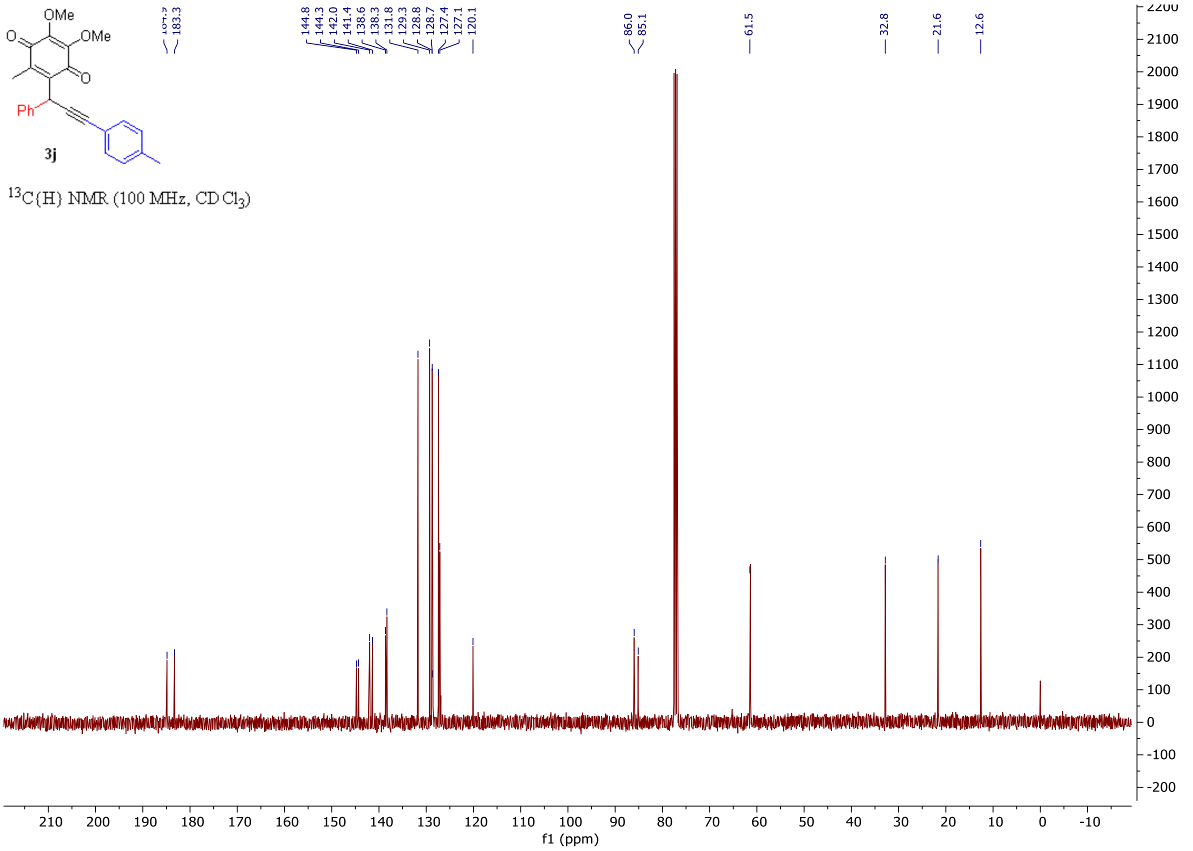
3j
^{13}C{H} NMR (100 MHz, CDCl$_3$)
f1 (ppm)

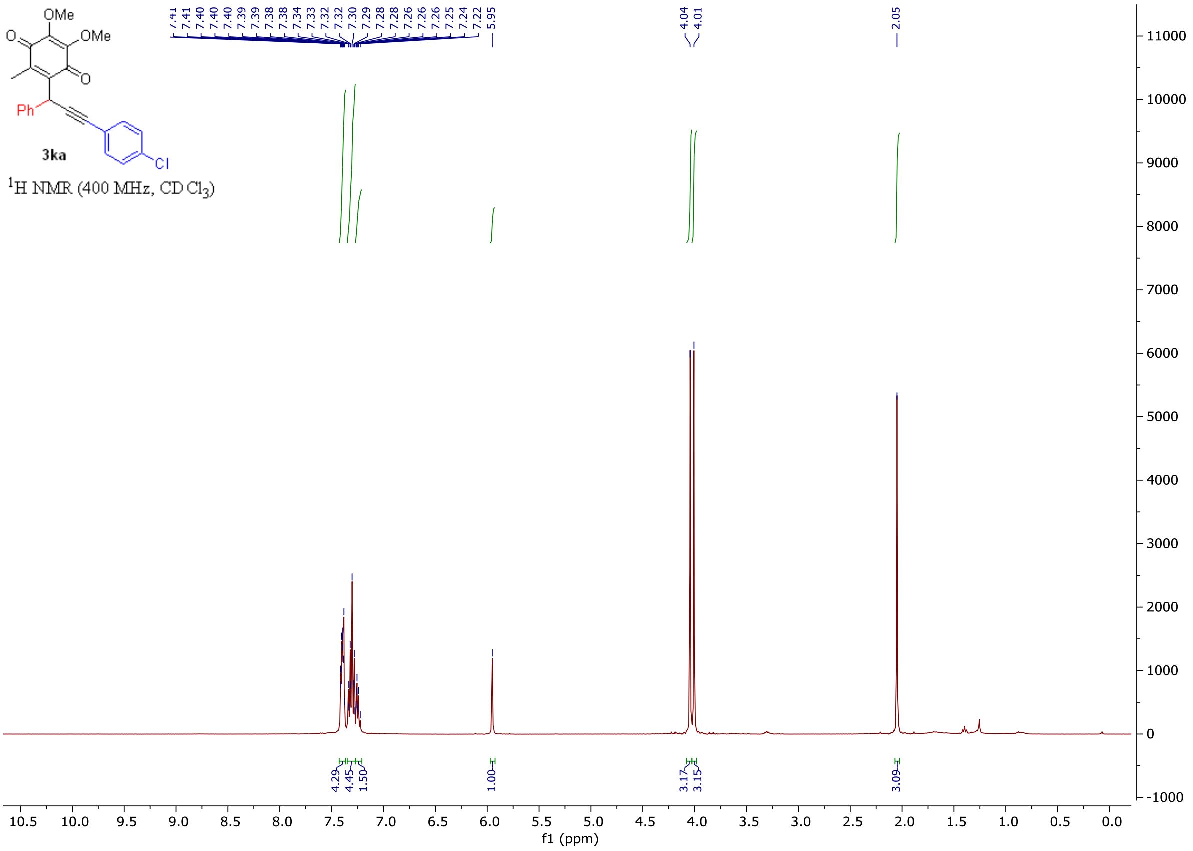
3ka
^{1}H NMR (400 MHz, CDCl$_3$)
f1 (ppm)

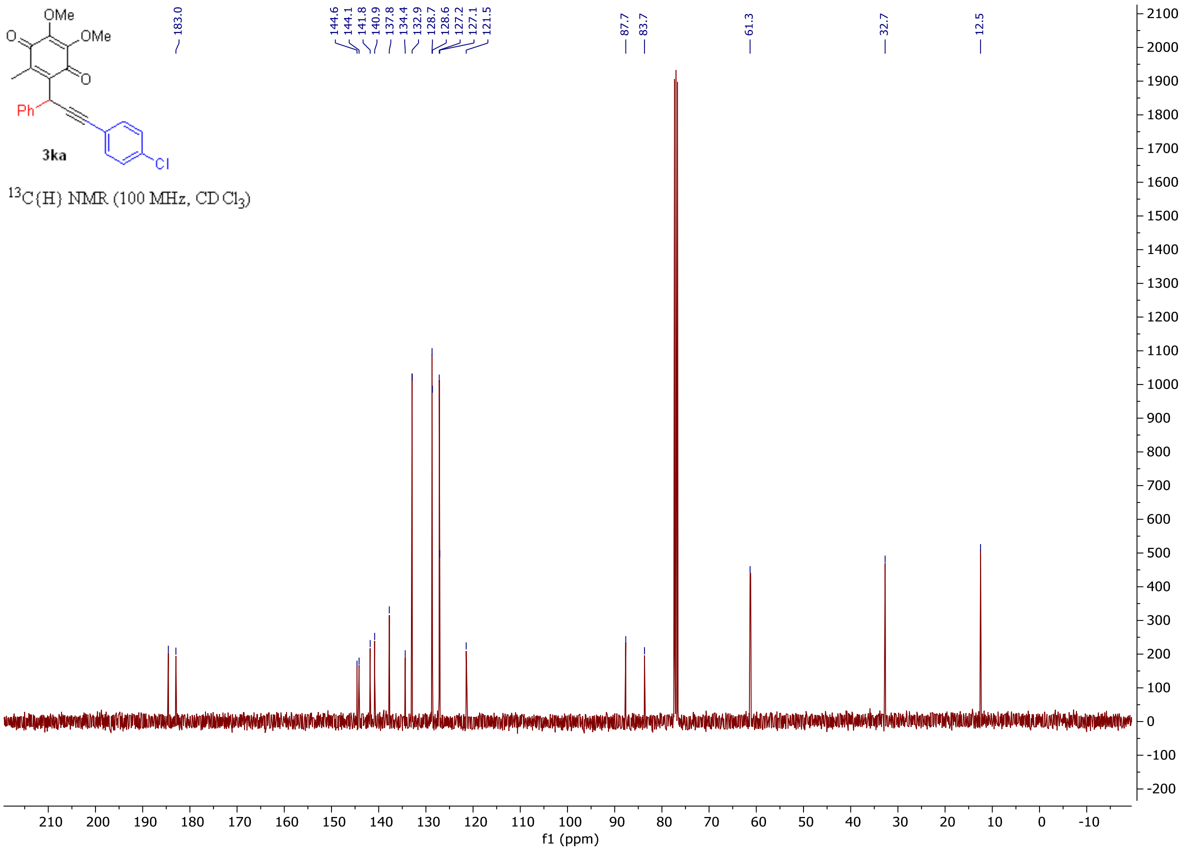

OMe
OMe
Ph
Cl
3ka
13C{H} NMR (100 MHz, CDCl3)
183.0
144.6
144.1
141.8
140.9
137.8
134.4
132.9
128.7
128.6
127.2
127.1
121.5
87.7
83.7
61.3
32.7
12.5
f1 (ppm)

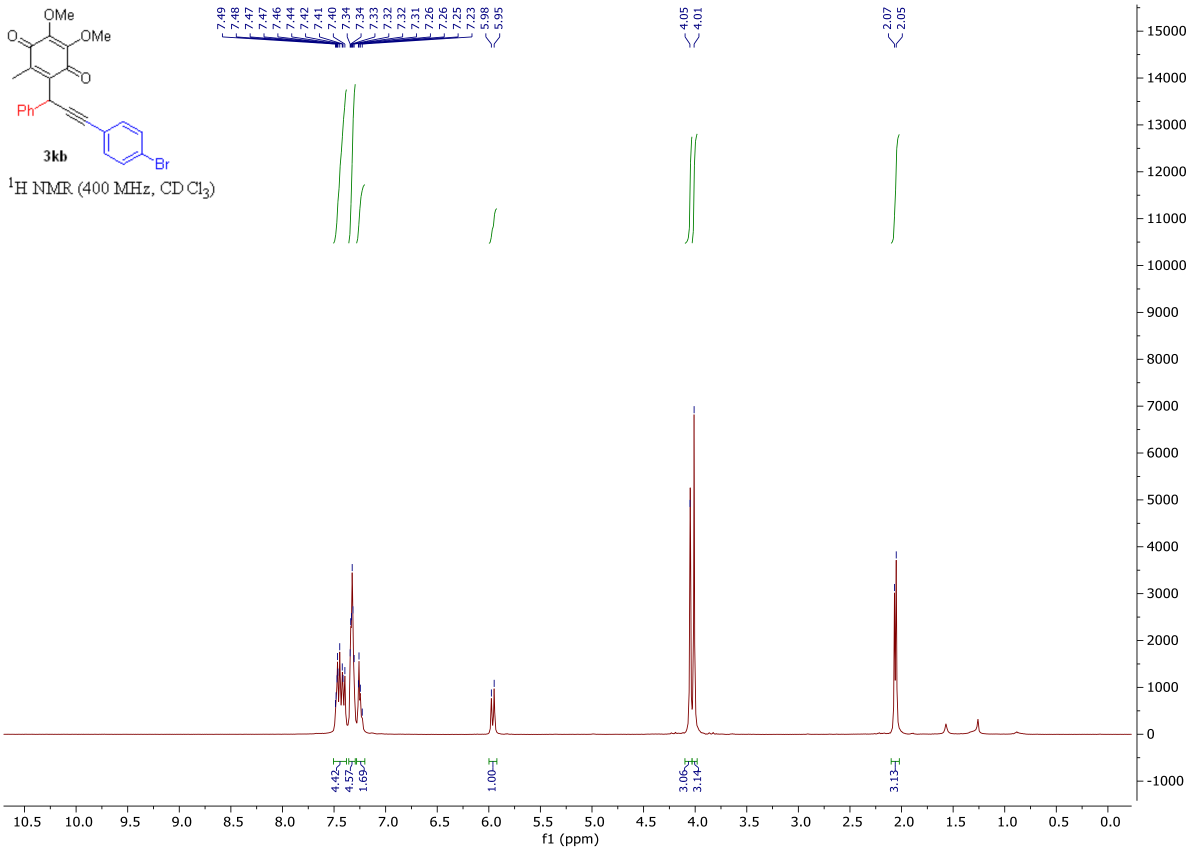

OMe
OMe
Ph
Br
3kb
1H NMR (400 MHz, CDCl3)
7.49
7.48
7.47
7.46
7.44
7.42
7.41
7.40
7.34
7.34
7.33
7.32
7.32
7.31
7.26
7.26
7.25
7.23
5.98
5.95
4.05
4.01
2.07
2.05
4.42
4.57
1.69
1.00
3.06
3.14
3.13
f1 (ppm)

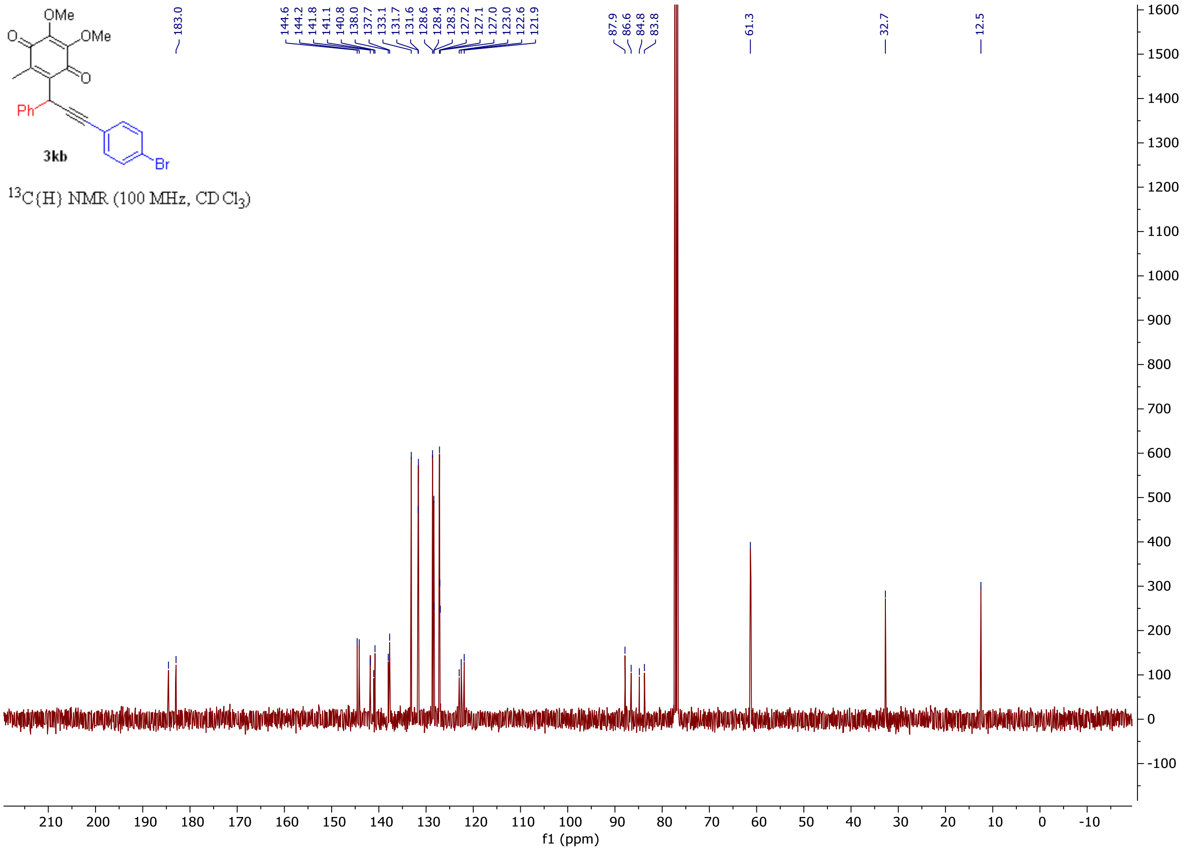
3kb
$^{13}C\{H\}$ NMR (100 MHz, $CDCl_3$)
f1 (ppm)

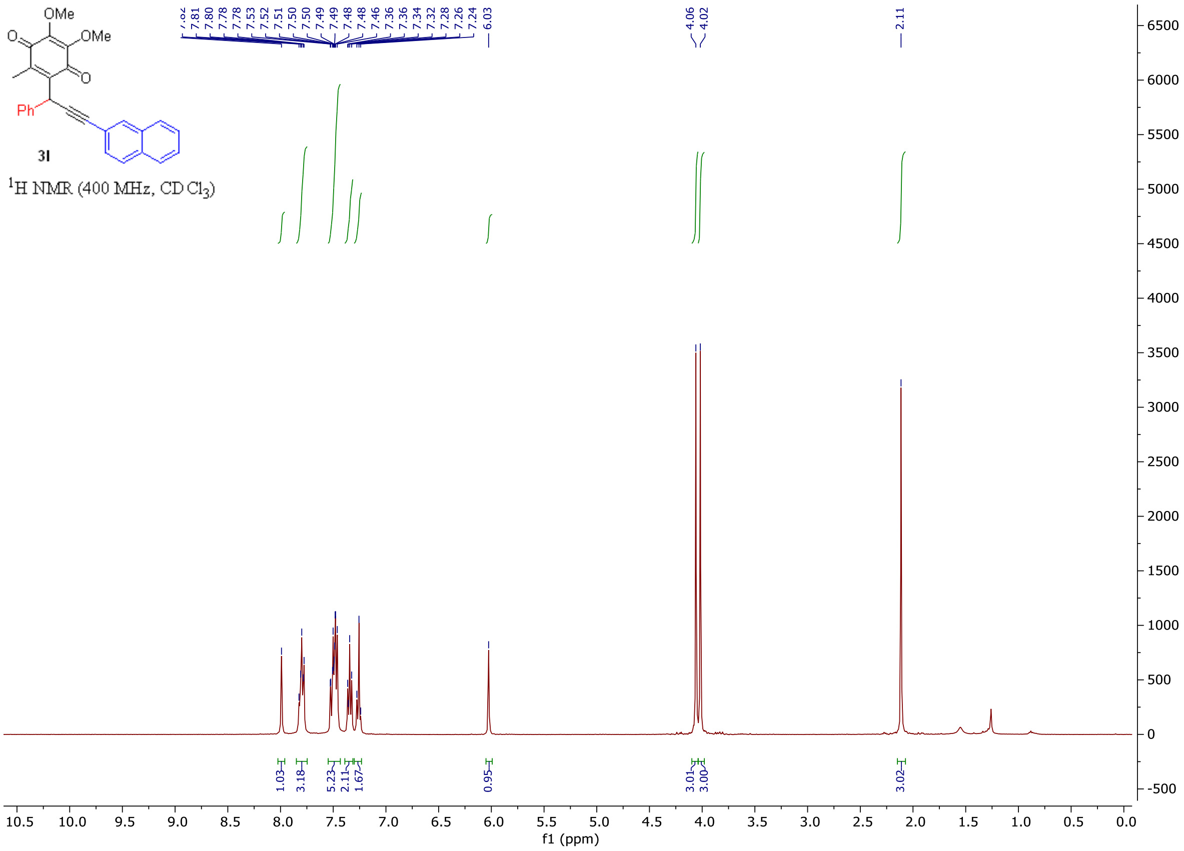
3l
^{1}H NMR (400 MHz, $CDCl_3$)
f1 (ppm)

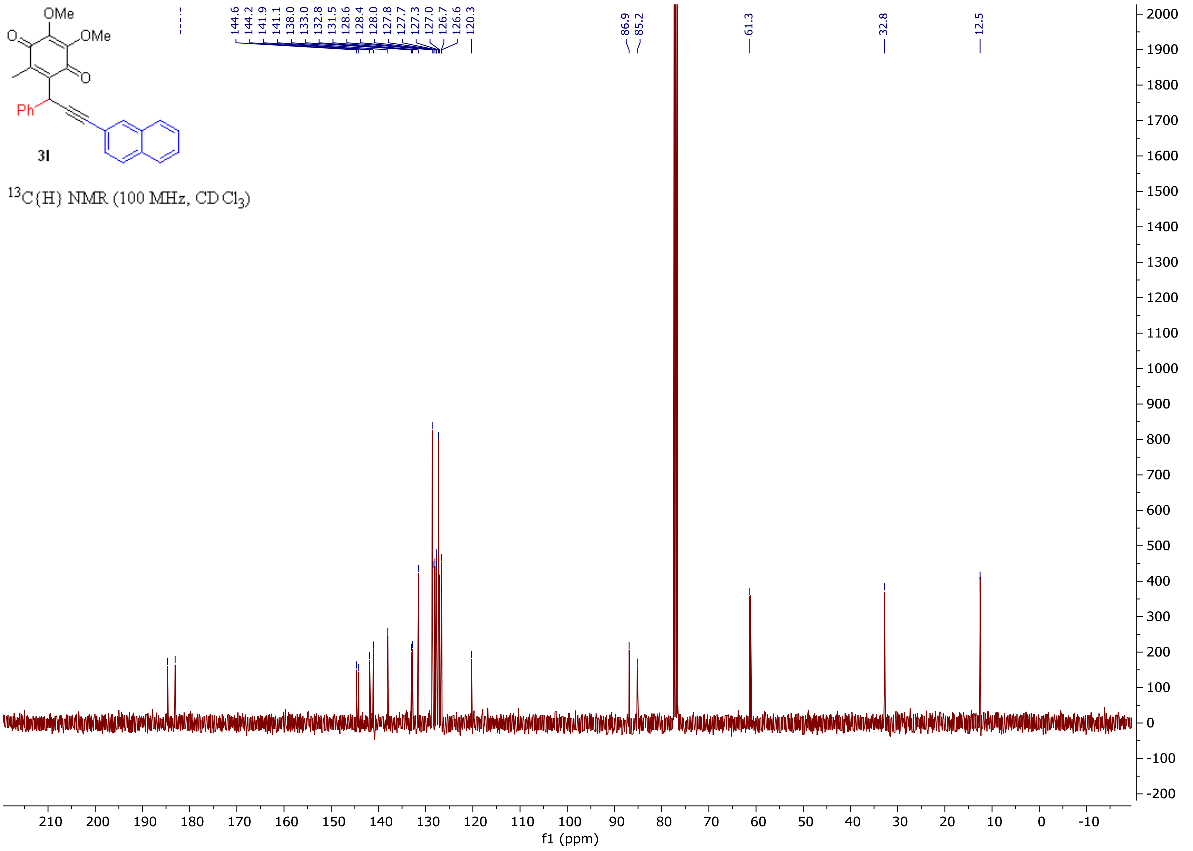
OMe
OMe
O
O
Ph
3l
¹³C{H} NMR (100 MHz, CDCl₃)
144.6
144.2
141.9
141.1
138.0
133.0
132.8
131.5
128.6
128.4
128.0
127.8
127.7
127.3
127.0
126.7
126.6
120.3
86.9
85.2
61.3
32.8
12.5
f1 (ppm)

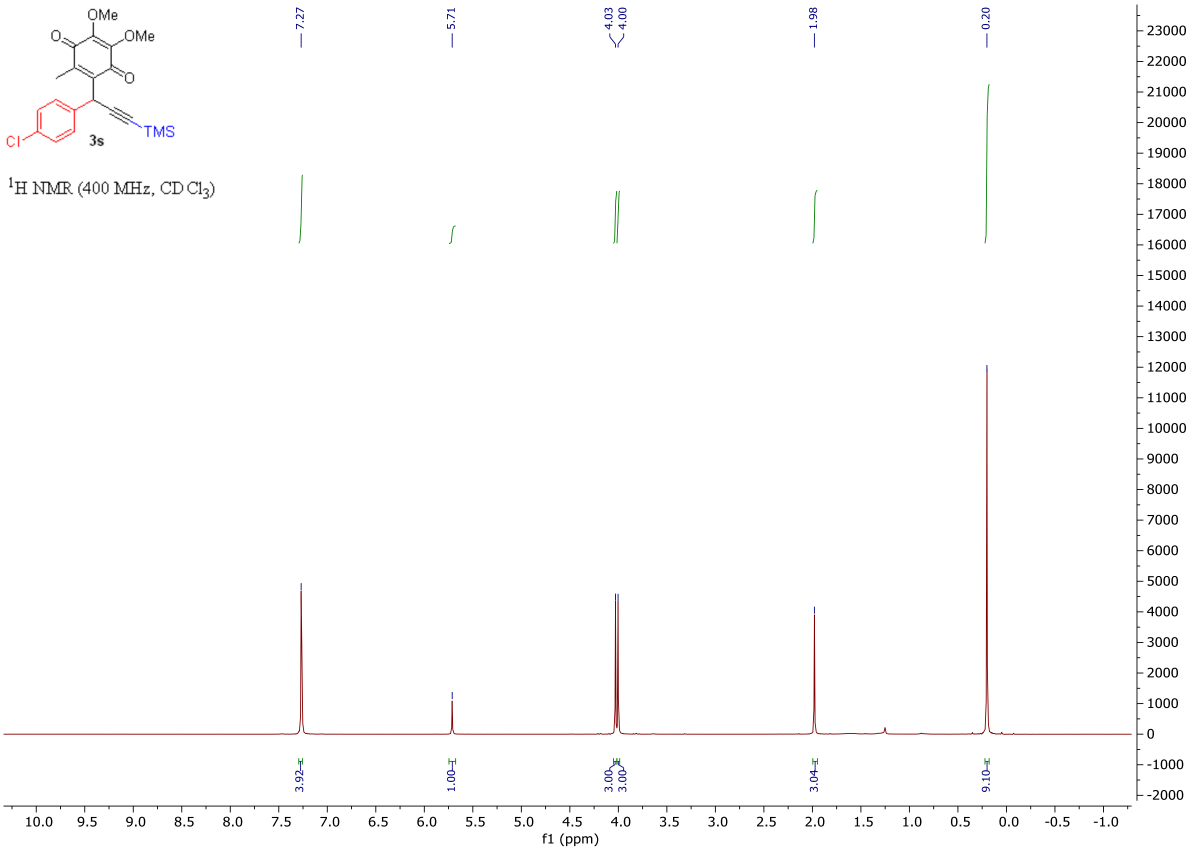
OMe
OMe
O
O
Cl
TMS
3s
¹H NMR (400 MHz, CDCl₃)
7.27
5.71
4.03
4.00
1.98
0.20
3.92
1.00
3.00
3.00
3.04
9.10
f1 (ppm)

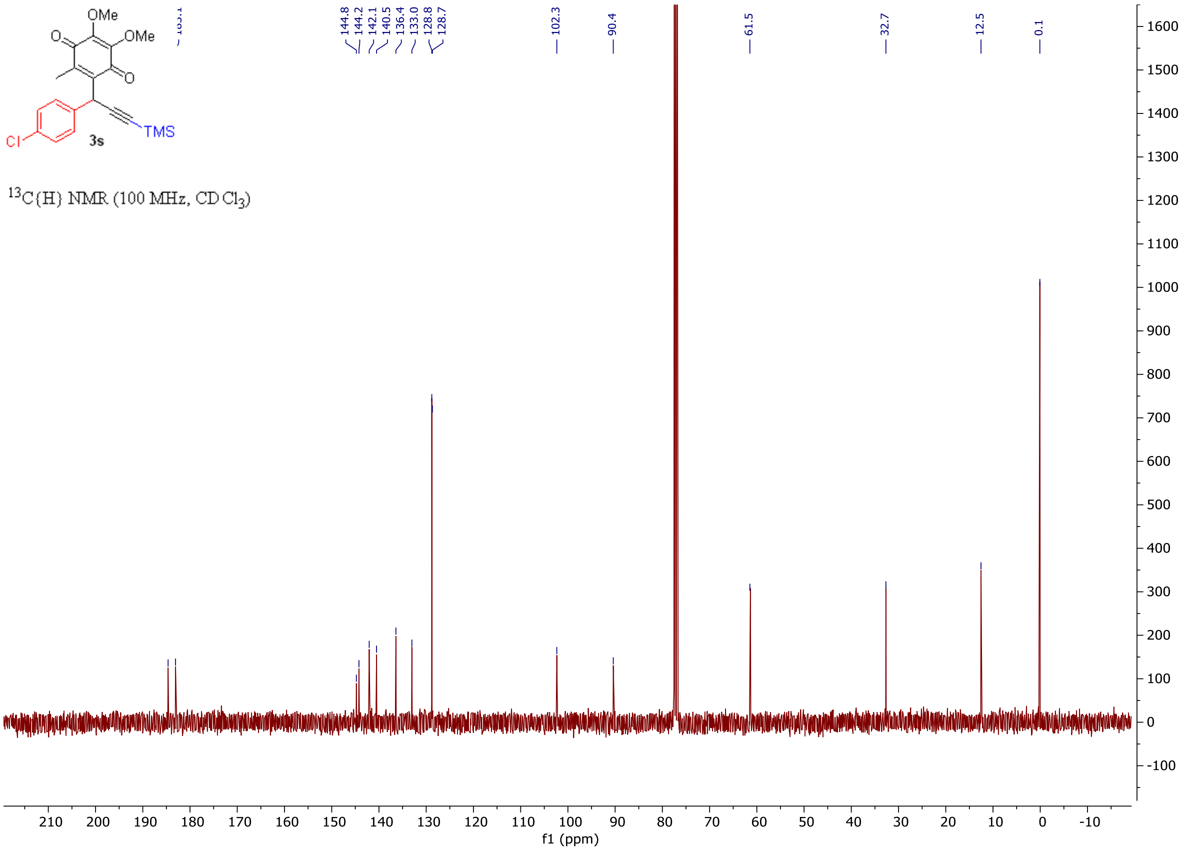
3s
TMS
$^{13}C\{H\}$ NMR (100 MHz, $CDCl_3$)
144.8
144.2
142.1
140.5
136.4
133.0
128.8
128.7
102.3
90.4
61.5
32.7
12.5
0.1
f1 (ppm)

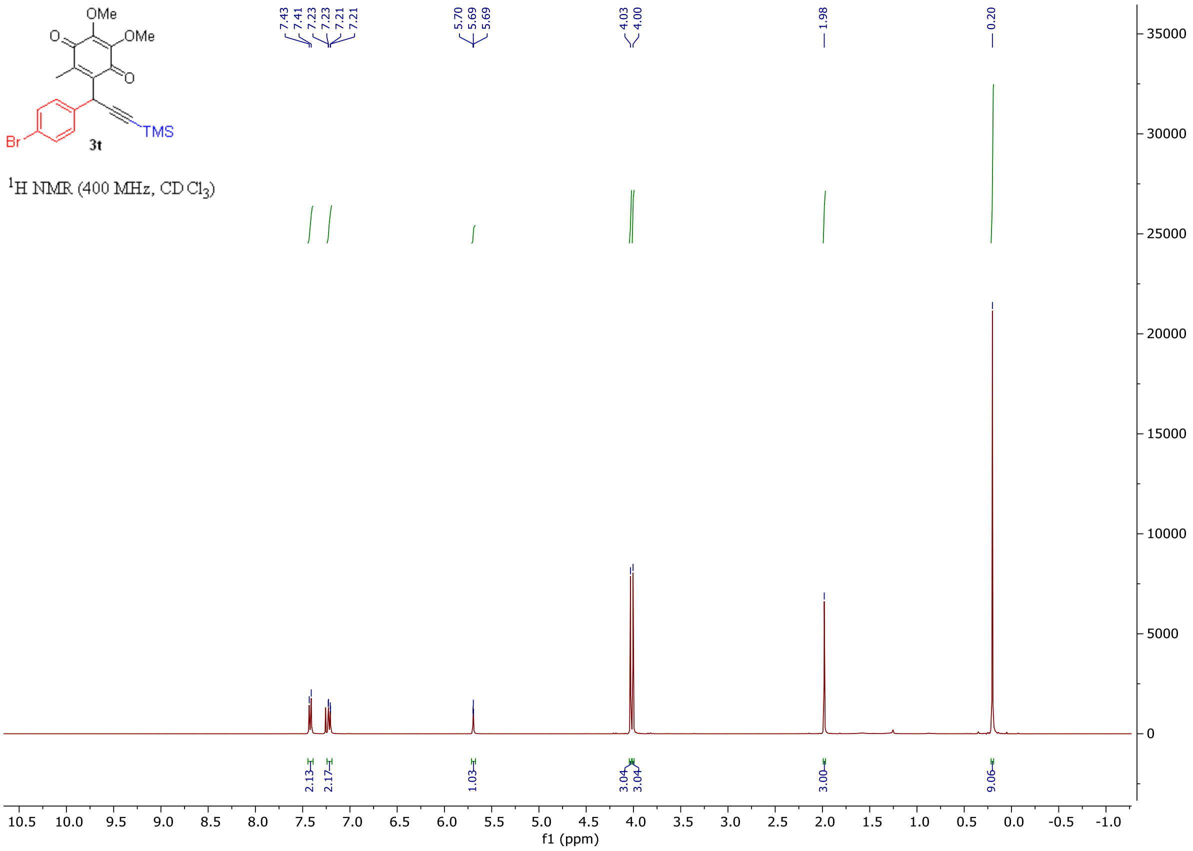
3t
TMS
^1H NMR (400 MHz, $CDCl_3$)
7.43
7.41
7.23
7.23
7.21
7.21
5.70
5.69
5.69
4.03
4.00
1.98
0.20
2.13
2.17
1.03
3.04
3.04
3.00
9.06
f1 (ppm)

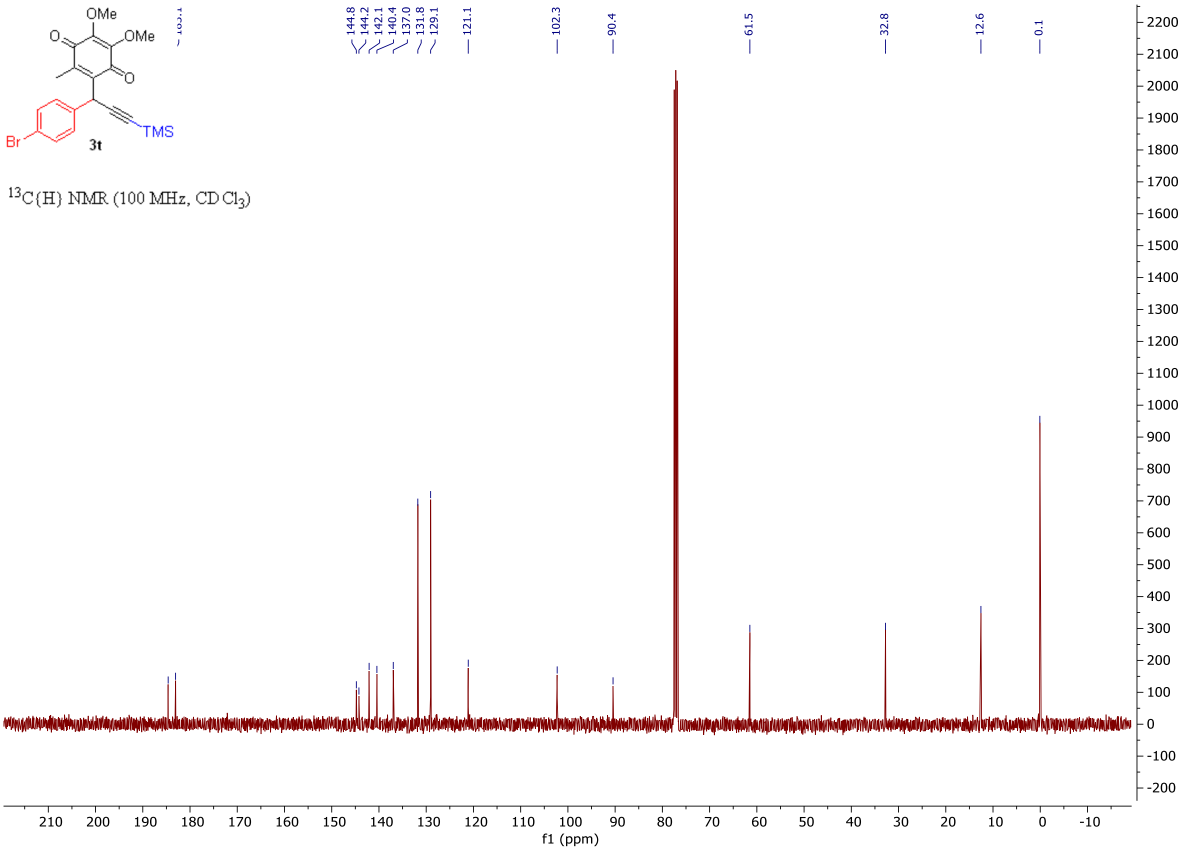
OMe
OMe
TMS
Br
3t
$^{13}C\{H\}$ NMR (100 MHz, $CDCl_3$)
183.1
144.8
144.2
142.1
140.4
137.0
131.8
129.1
121.1
102.3
90.4
61.5
32.8
12.6
0.1
f1 (ppm)

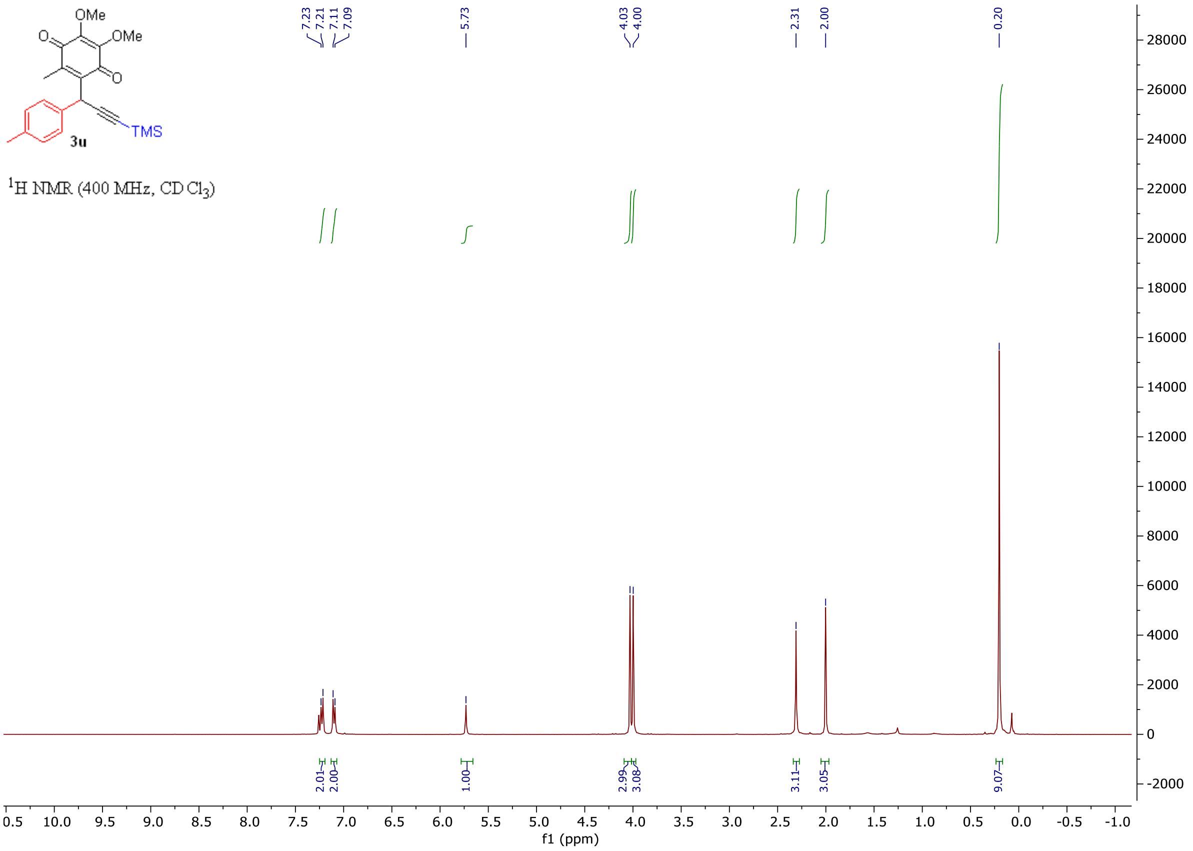
OMe
OMe
TMS
3u
^{1}H NMR (400 MHz, $CDCl_3$)
7.23
7.21
7.11
7.09
5.73
4.03
4.00
2.31
2.00
0.20
2.01
2.00
1.00
2.99
3.08
3.11
3.05
9.07
f1 (ppm)

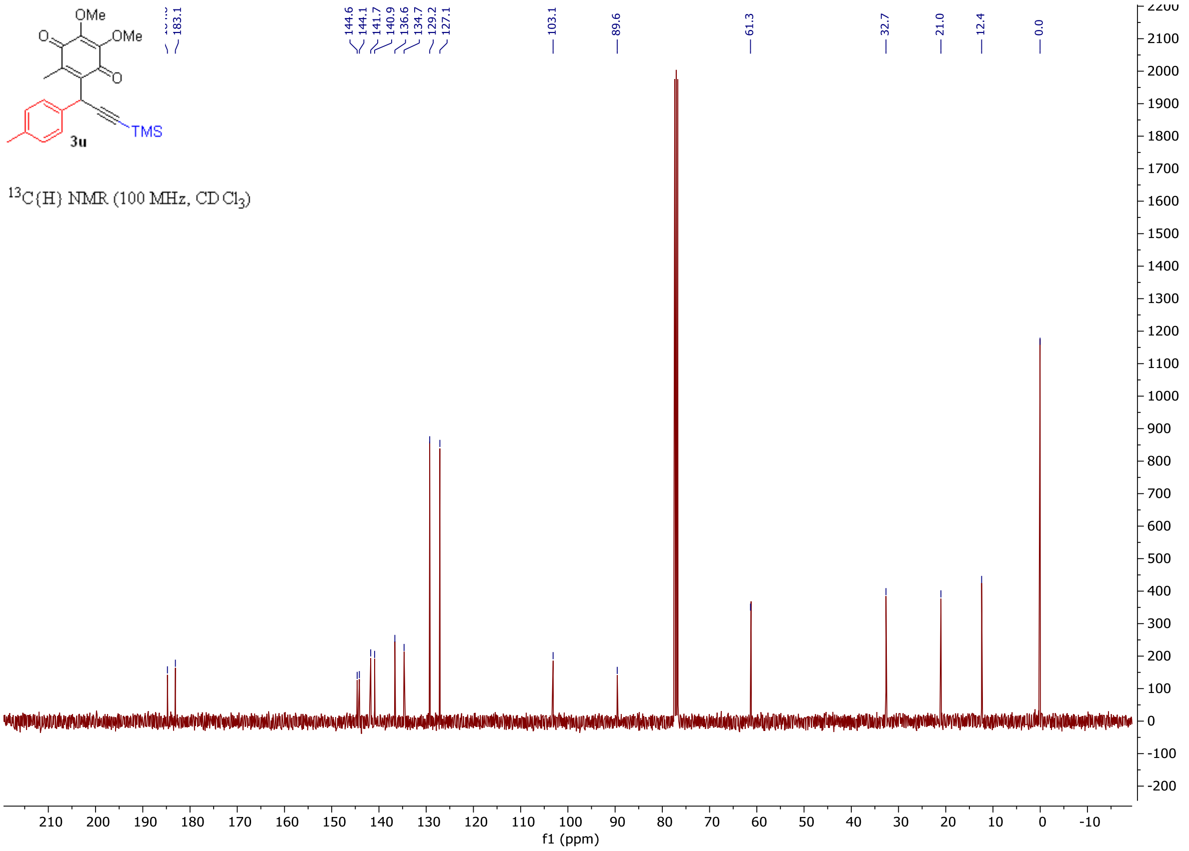
OMe
OMe
TMS
3u
13C{H} NMR (100 MHz, CDCl3)
183.1
144.6
144.1
141.7
140.9
136.6
134.7
129.2
127.1
103.1
89.6
61.3
32.7
21.0
12.4
0.0
f1 (ppm)

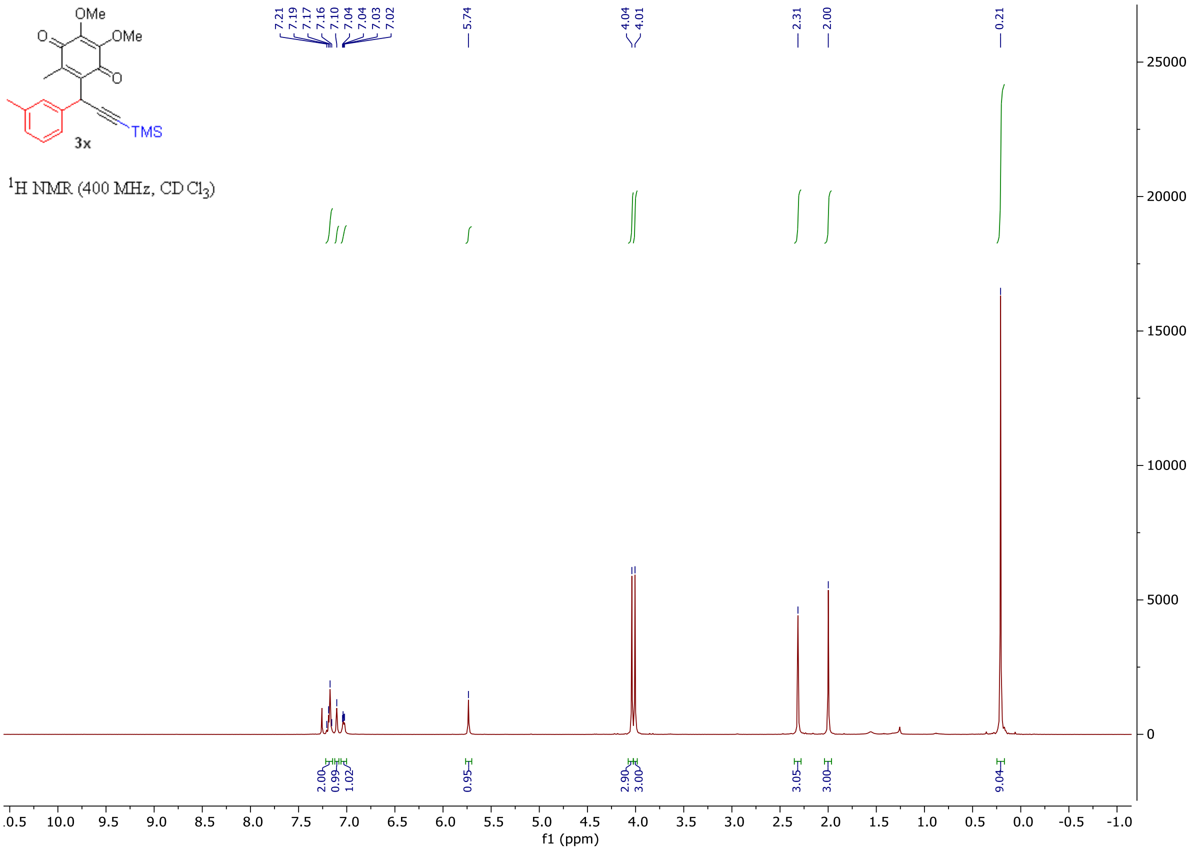
OMe
OMe
TMS
3x
1H NMR (400 MHz, CDCl3)
7.21
7.19
7.17
7.16
7.10
7.04
7.04
7.03
7.02
5.74
4.04
4.01
2.31
2.00
0.21
2.00
0.99
1.02
0.95
2.90
3.00
3.05
3.00
9.04
f1 (ppm)

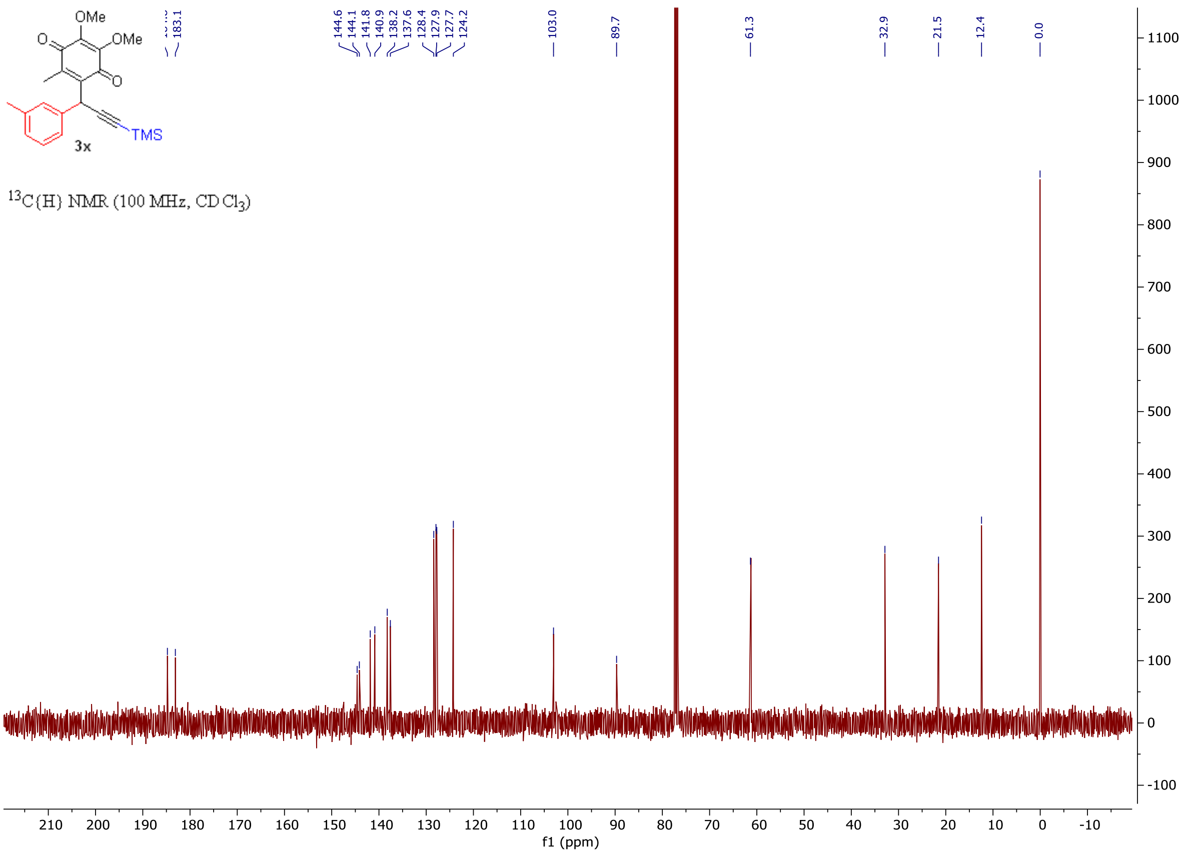

OMe
OMe
O
O
TMS
3x
$^{13}C\{H\}$ NMR (100 MHz, CDCl$_3$)
183.1
144.6
144.1
141.8
140.9
138.2
137.6
128.4
127.9
127.7
124.2
103.0
89.7
61.3
32.9
21.5
12.4
0.0
f1 (ppm)

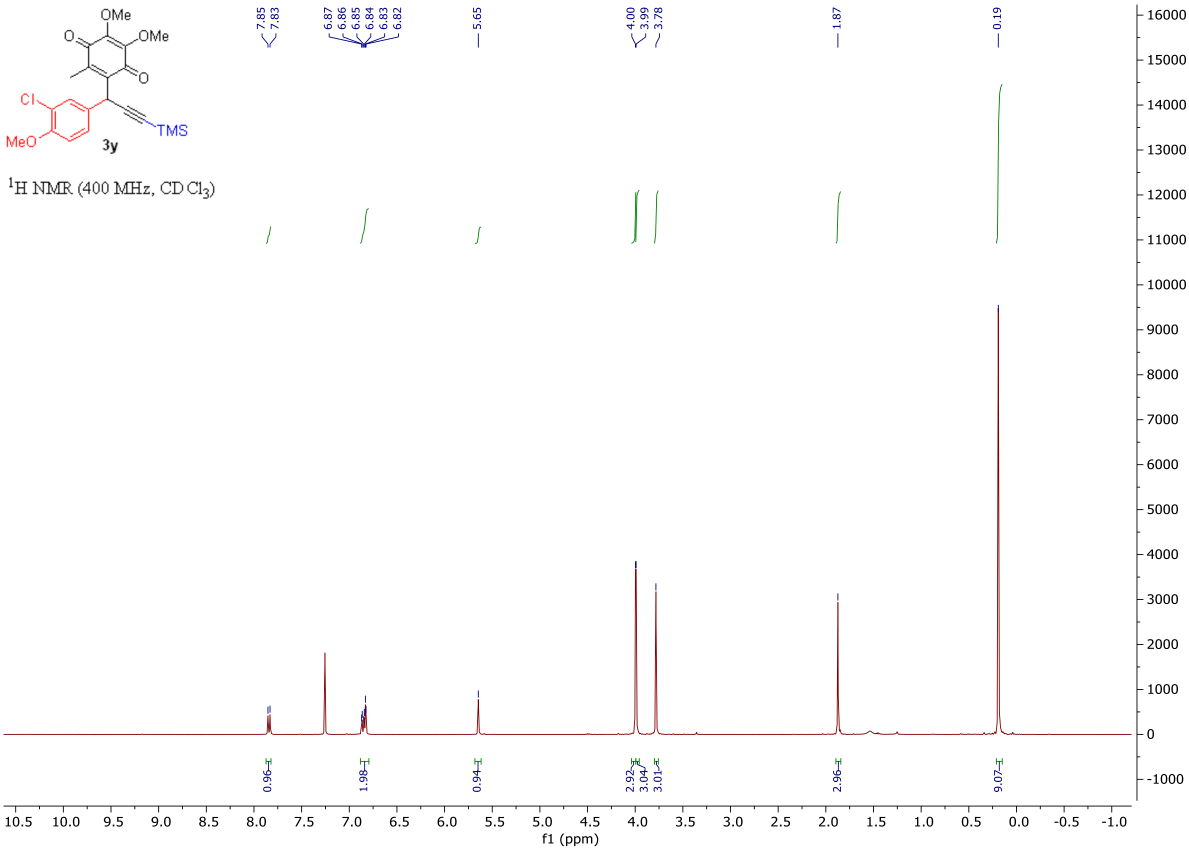

OMe
OMe
O
O
Cl
MeO
TMS
3y
^{1}H NMR (400 MHz, CDCl$_3$)
7.85
7.83
6.87
6.86
6.85
6.84
6.83
6.82
5.65
4.00
3.99
3.78
1.87
0.19
0.96
1.98
0.94
2.92
3.04
3.01
2.96
9.07
f1 (ppm)

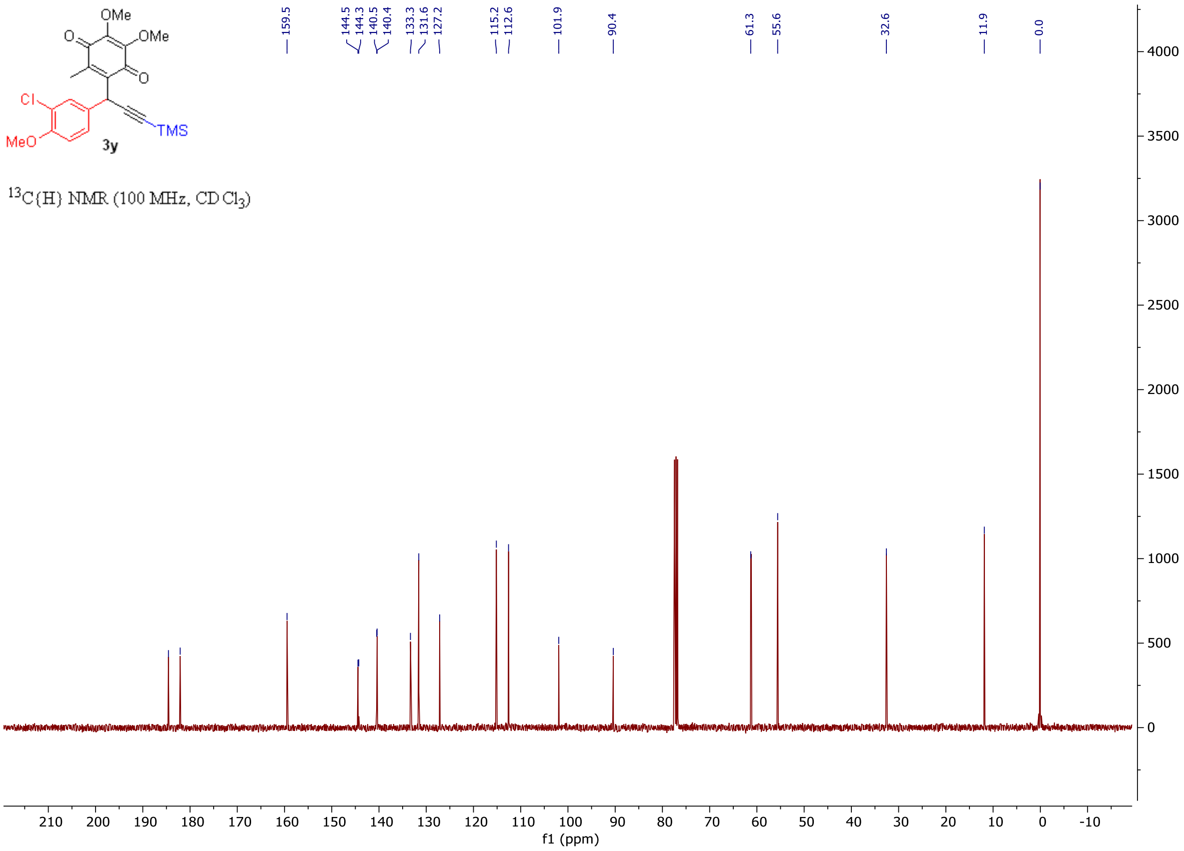
OMe
O
OMe
O
Cl
MeO
TMS
3y
^{13}C{H} NMR (100 MHz, CDCl$_3$)
f1 (ppm)

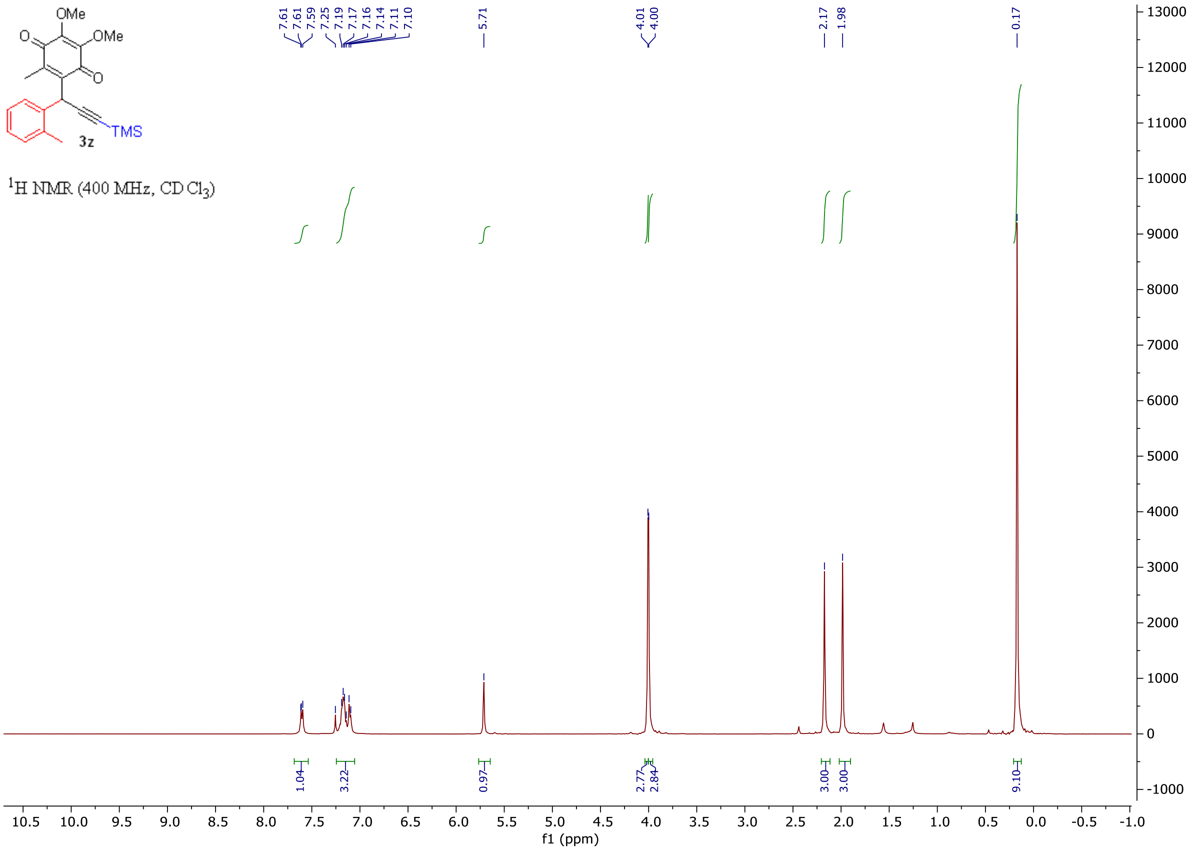
OMe
O
OMe
O
TMS
3z
^{1}H NMR (400 MHz, CDCl$_3$)
f1 (ppm)

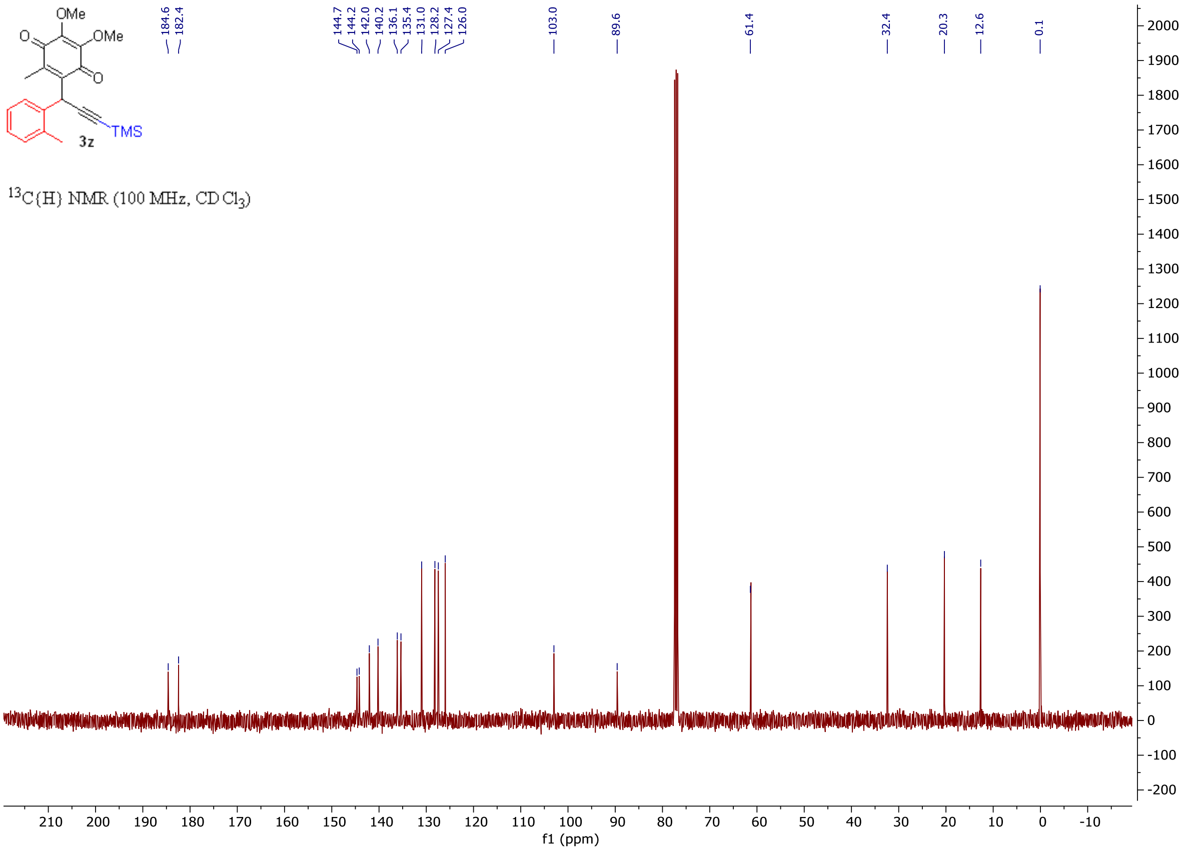

OMe
OMe
TMS
3z
$^{13}C\{H\}$ NMR (100 MHz, $CDCl_3$)
184.6
182.4
144.7
144.2
142.0
140.2
136.1
135.4
131.0
128.2
127.4
126.0
103.0
89.6
61.4
32.4
20.3
12.6
0.1
f1 (ppm)

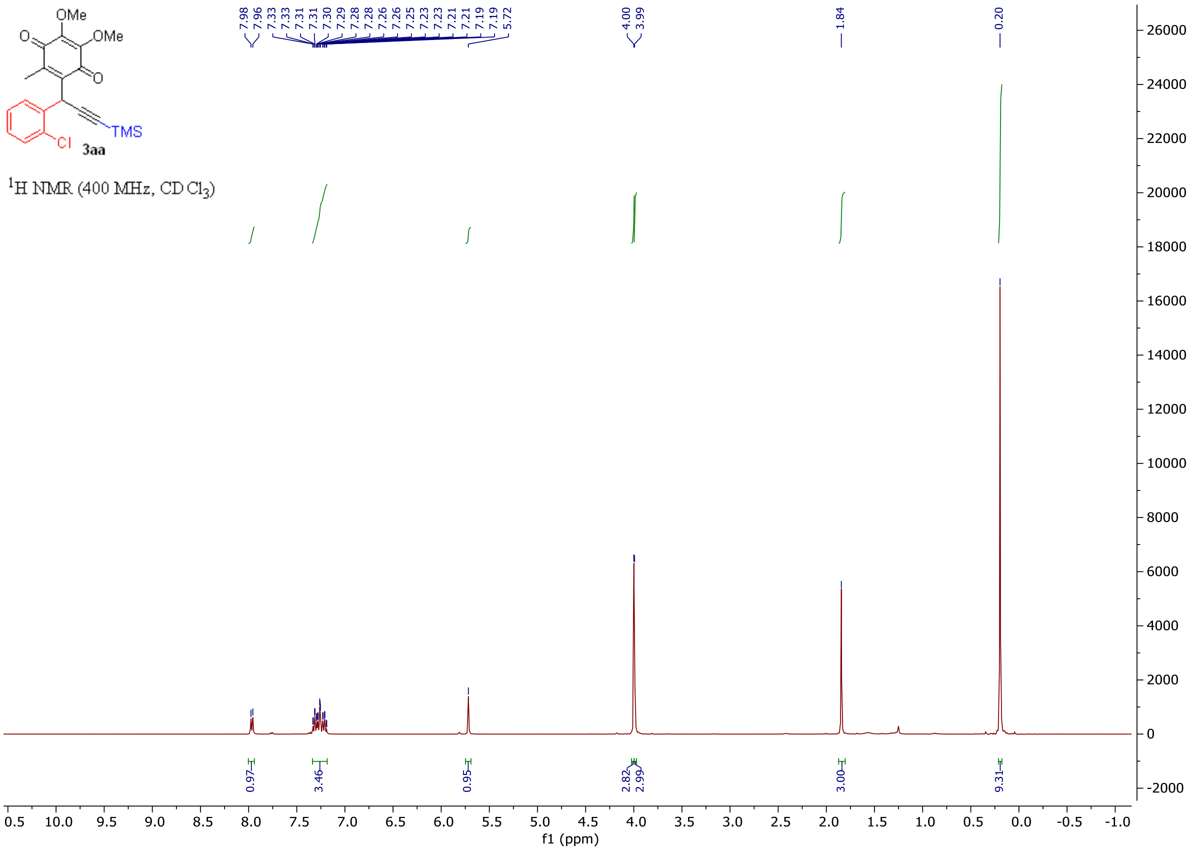

OMe
OMe
TMS
Cl
3aa
^{1}H NMR (400 MHz, $CDCl_3$)
7.98
7.96
7.33
7.33
7.31
7.30
7.29
7.28
7.28
7.26
7.26
7.25
7.23
7.23
7.21
7.19
7.19
5.72
4.00
3.99
1.84
0.20
0.97
3.46
0.95
2.82
2.99
3.00
9.31
f1 (ppm)

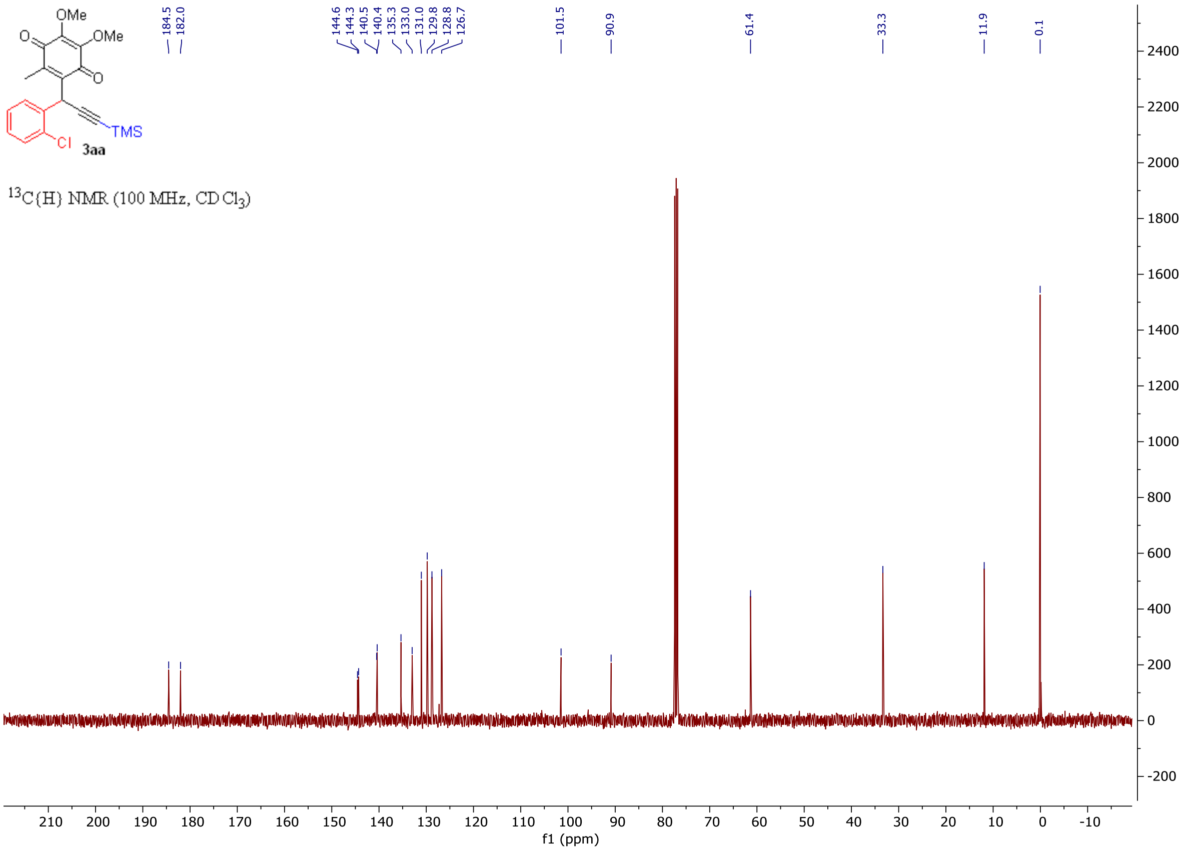
3aa
13C{H} NMR (100 MHz, CDCl3)
184.5
182.0
144.6
144.3
140.5
140.4
135.3
133.0
131.0
129.8
128.8
126.7
101.5
90.9
61.4
33.3
11.9
0.1
f1 (ppm)

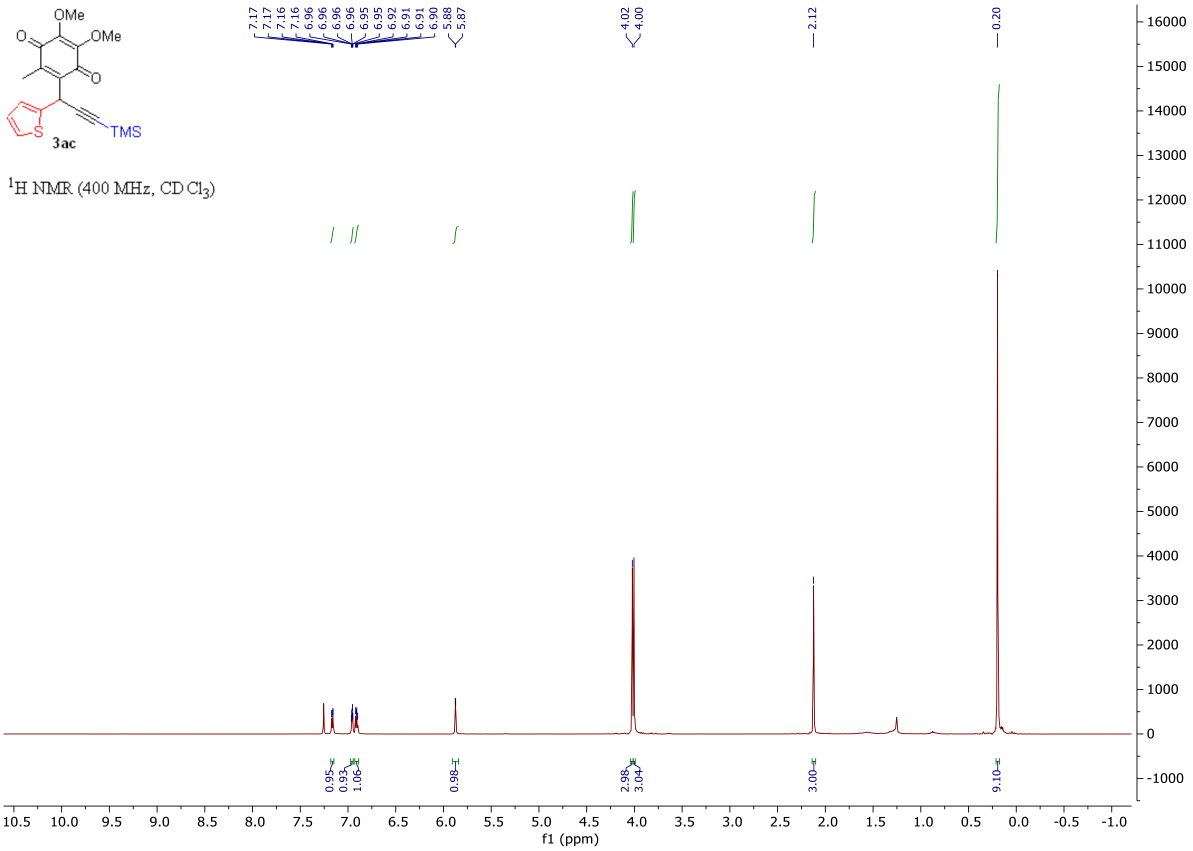
3ac
1H NMR (400 MHz, CDCl3)
7.17
7.17
7.16
6.96
6.96
6.96
6.95
6.95
6.92
6.91
6.91
6.90
5.88
5.87
4.02
4.00
2.12
0.20
0.95
0.93
1.06
0.98
2.98
3.04
3.00
9.10
f1 (ppm)

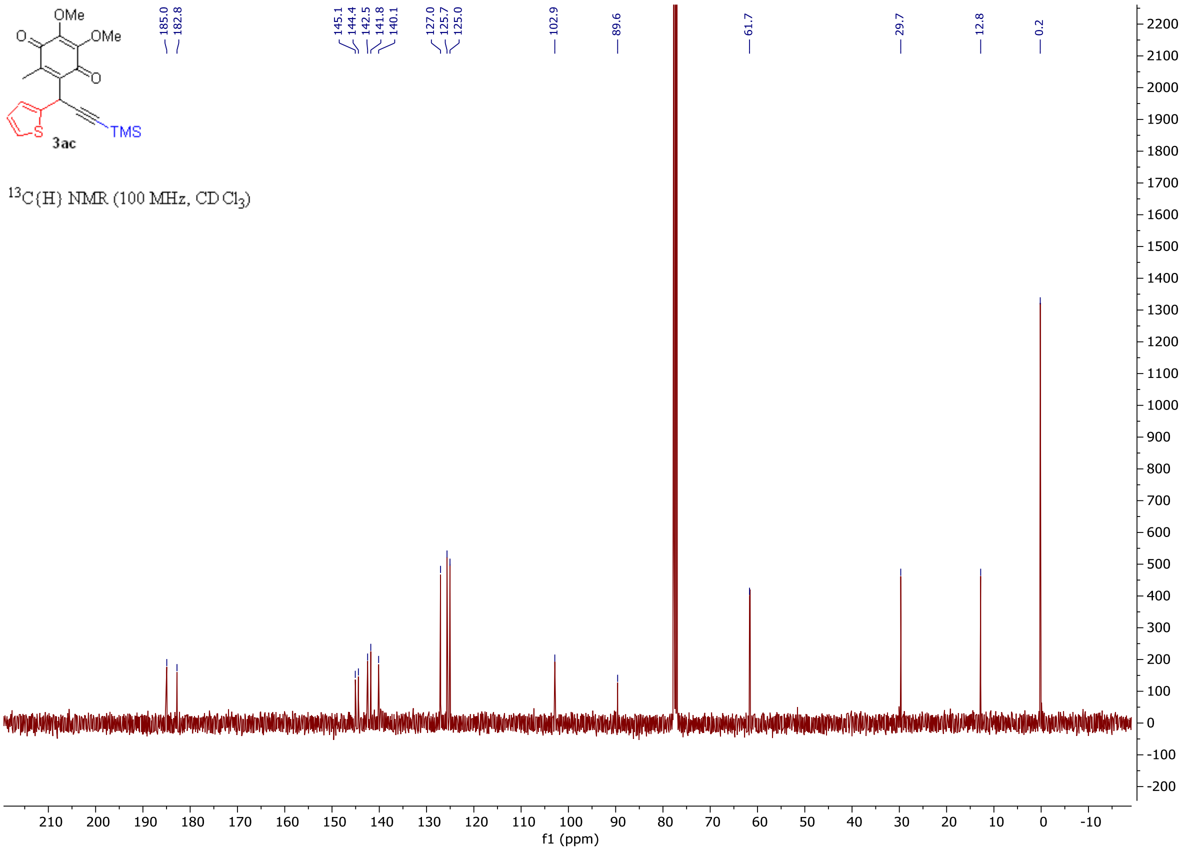
OMe
OMe
TMS
3ac
$^{13}C\{H\}$ NMR (100 MHz, CDCl$_3$)
185.0
182.8
145.1
144.4
142.5
141.8
140.1
127.0
125.7
125.0
102.9
89.6
61.7
29.7
12.8
0.2
f1 (ppm)

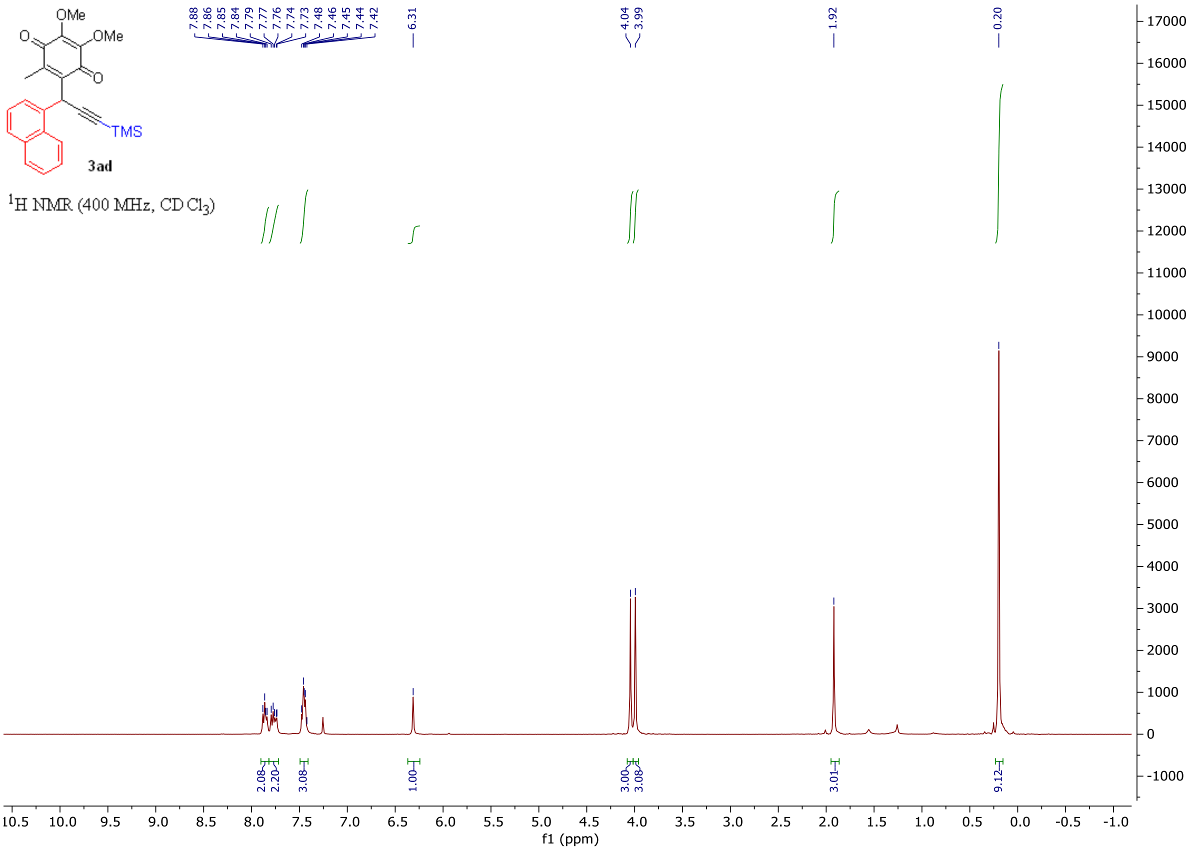
OMe
OMe
TMS
3ad
^{1}H NMR (400 MHz, CDCl$_3$)
7.88
7.86
7.85
7.84
7.79
7.77
7.76
7.74
7.73
7.48
7.46
7.45
7.44
7.42
6.31
4.04
3.99
1.92
0.20
2.08
2.20
3.08
1.00
3.00
3.08
3.01
9.12
f1 (ppm)

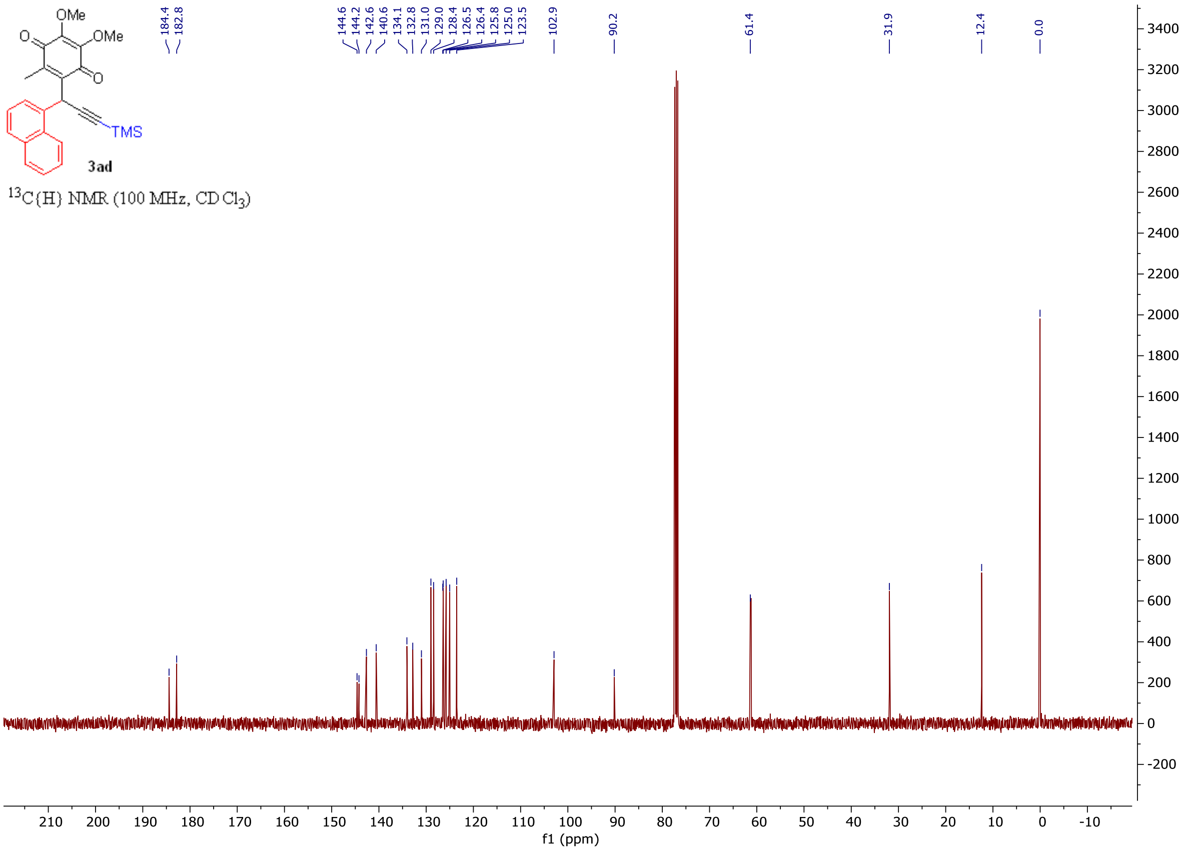
OMe
OMe
O
O
TMS
3ad
$^{13}C\{H\}$ NMR (100 MHz, $CDCl_3$)
184.4
182.8
144.6
144.2
142.6
140.6
134.1
132.8
131.0
129.0
128.4
126.5
126.4
125.8
125.0
123.5
102.9
90.2
61.4
31.9
12.4
0.0
f1 (ppm)

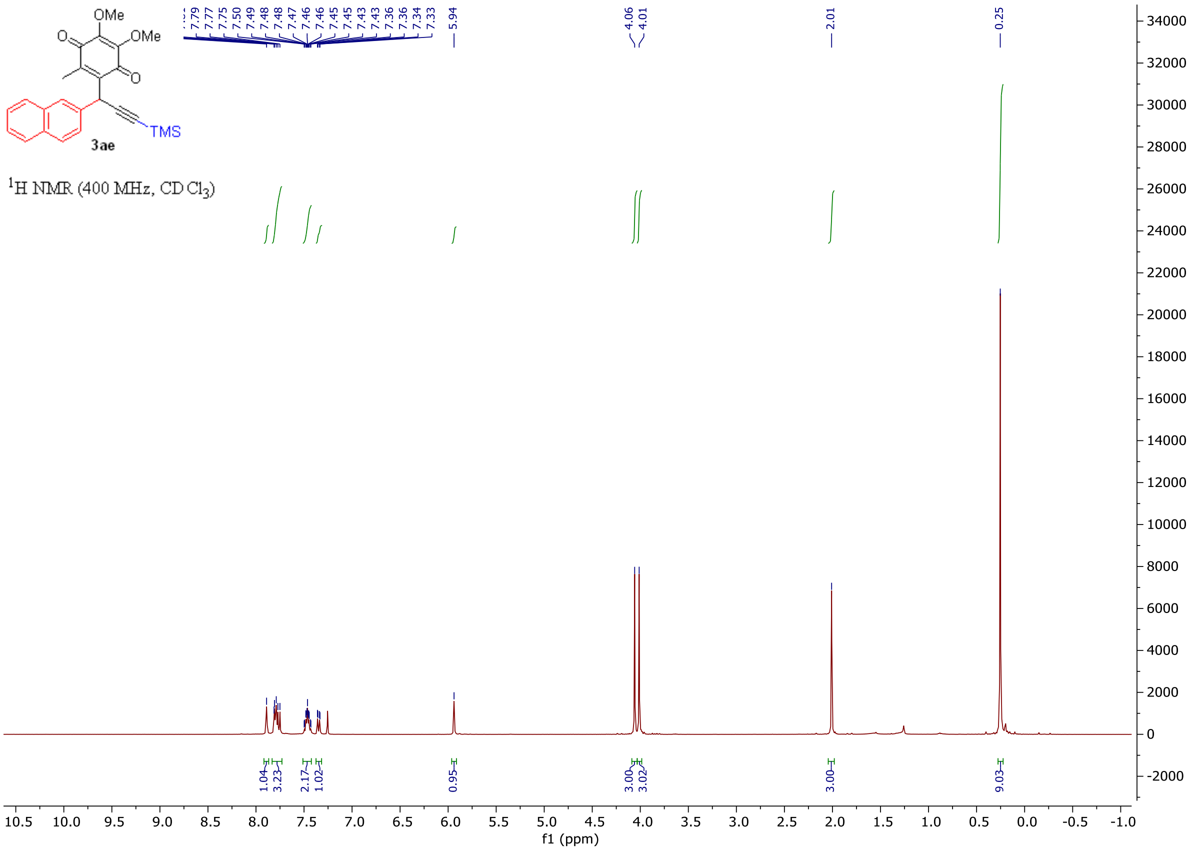
OMe
OMe
O
O
TMS
3ae
^1H NMR (400 MHz, $CDCl_3$)
7.79
7.77
7.75
7.50
7.49
7.48
7.48
7.47
7.46
7.46
7.45
7.45
7.43
7.36
7.36
7.34
7.33
5.94
4.06
4.01
2.01
0.25
1.04
3.23
2.17
1.02
0.95
3.00
3.02
3.00
9.03
f1 (ppm)

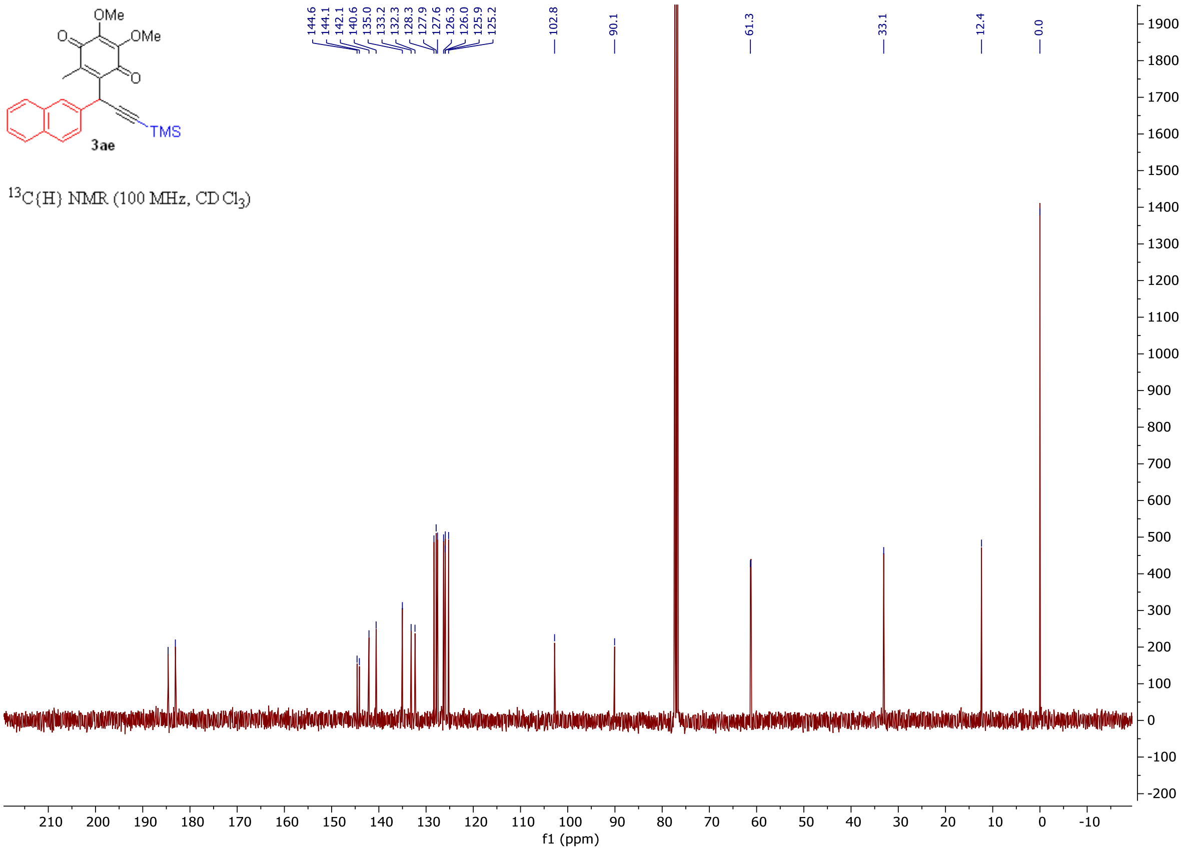
OMe
OMe
TMS
3ae
13C{H} NMR (100 MHz, CDCl3)
144.6
144.1
142.1
140.6
135.0
133.2
132.3
128.3
127.9
127.6
126.3
126.0
125.9
125.2
102.8
90.1
61.3
33.1
12.4
0.0
f1 (ppm)

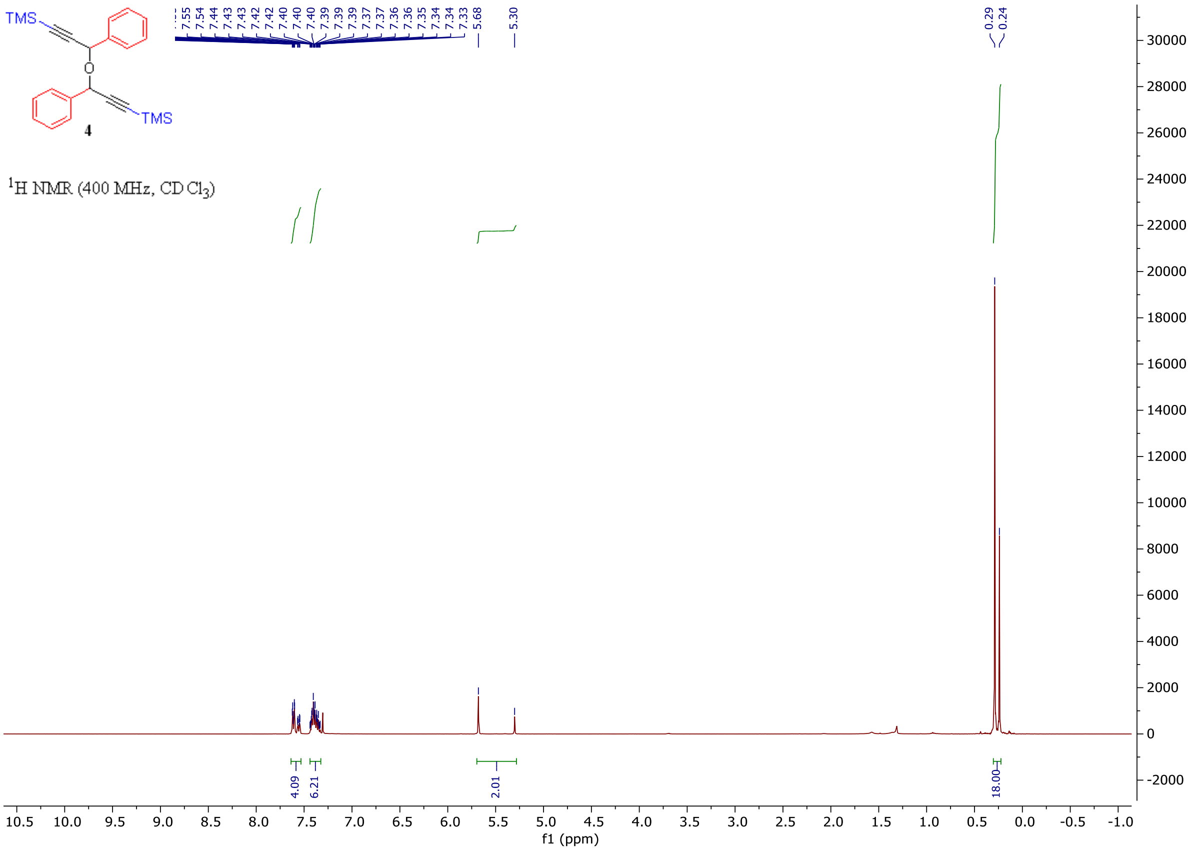
TMS
TMS
4
1H NMR (400 MHz, CDCl3)
5.68
5.30
0.29
0.24
4.09
6.21
2.01
18.00
f1 (ppm)

2. Mechanistic studies – LCMS analysis of raw reaction mixture

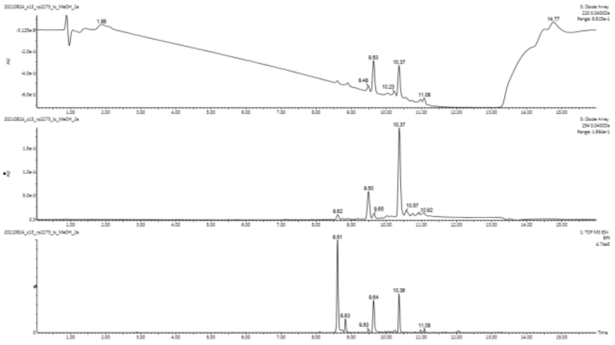

Fig. 1 UV chromatograms (λ=220 nm – top, λ=254 nm – middle) and Base Peak chromatogram – bottom for sample: QAT-163

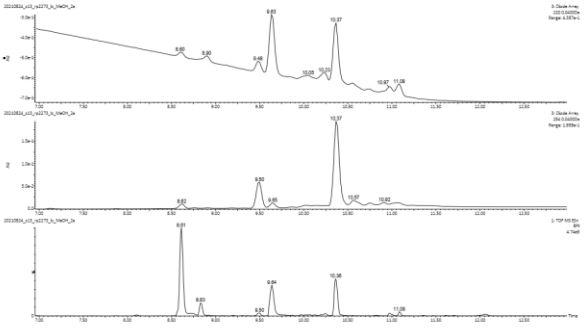

Fig. 2 Close -ups of UV chromatograms (λ=220 nm – top, λ=254 nm – middle) and Base Peak chromatogram – bottom for sample: QAT-163

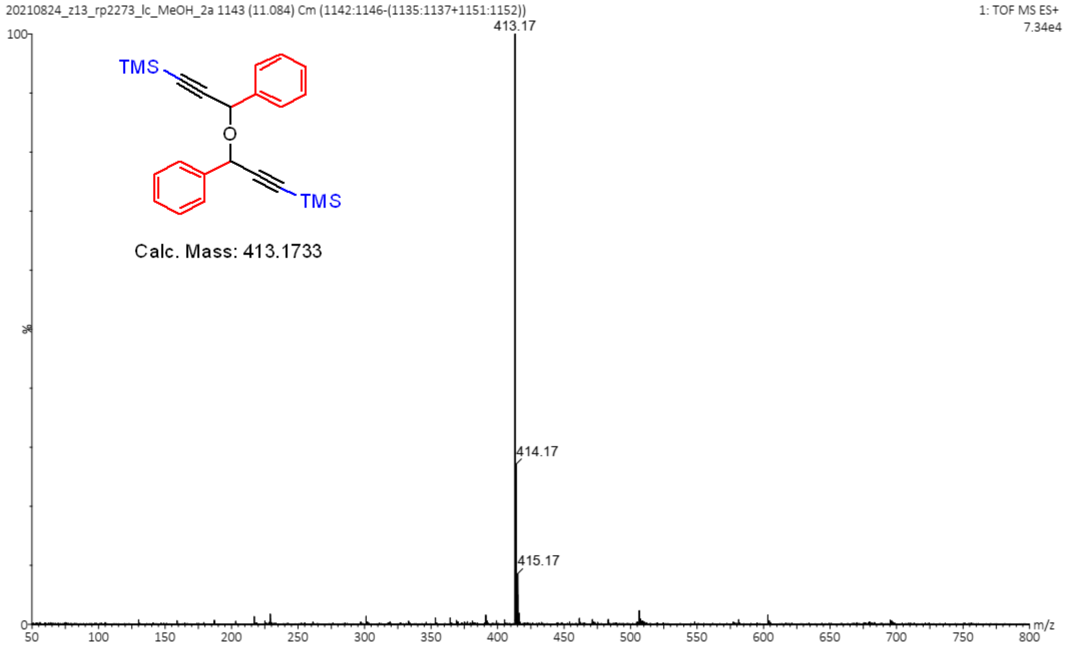

Fig.11 MS spectrum of the rt=11.08 peak

Experimental Instrument Parameters - MS	QAT-163
Polarity	ESI +
Capillary Voltage (kV)	3.00
Source temp. [C]	120.0
Sampling Cone	60.0
Source Offset	50.0
Source Gas Flow (mL/min)	0.00
Desolvation temp. [C]	250.0
Cone Gas [L/h]	100.0
Desolvation Gas Flow [L/h]	700.0
Nebuliser Gas Flow[bar]	4.5

Experimental Instrument Parameters - LC	QAT-163			
Solvent A	0.1% formic acid in water			
Solvent B	Methanol			
Run time	16.00 min			
Flow rate	0.30 ml/min			
Gradient table	time [min]	%A	%B	Curve
	0.00	95	5	-
	0.50	95	5	6
	10.00	0	100	6
	12.00	0	100	6
	13.00	95	5	6
	16.00	95	5	6

QAT-134

z13_rp2209 7 (0.245) Cm (7:9)

1: TOF MS ES+
1.36e5

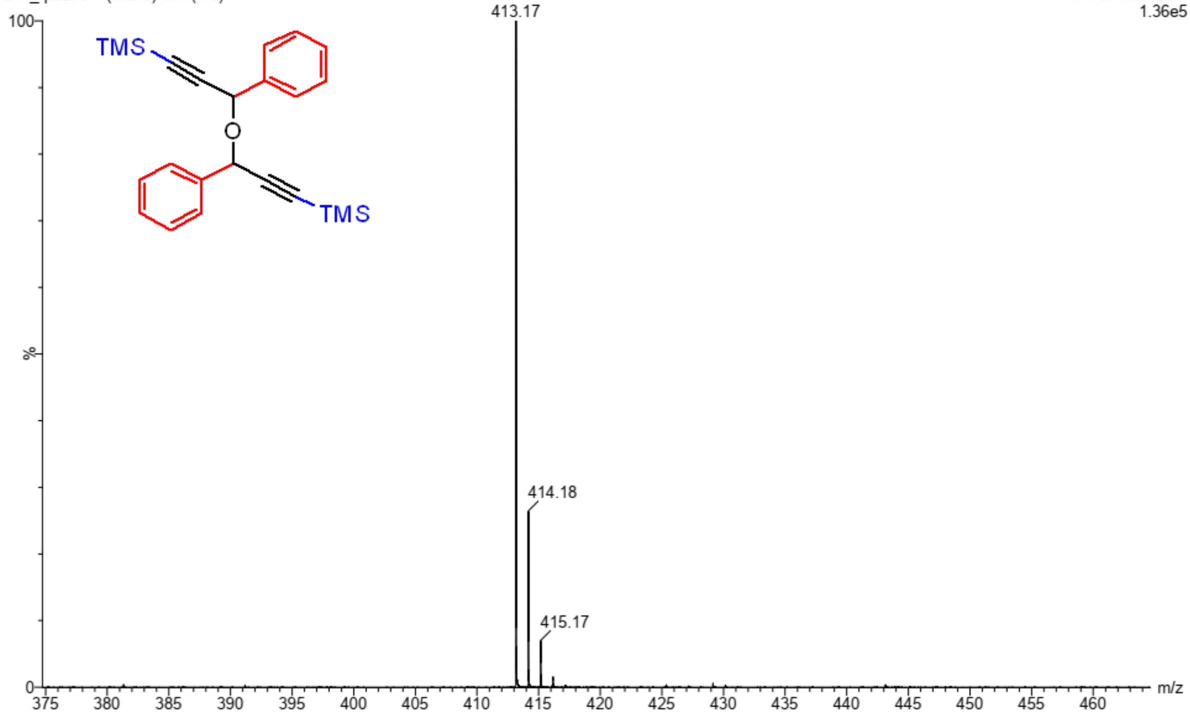

Single Mass Analysis

Tolerance = 3.0 mDa / DBE: min = -1.5, max = 100.0

Element prediction: Off

Number of isotope peaks used for i-FIT = 3

Monoisotopic Mass, Even Electron Ions

20 formula(e) evaluated with 2 results within limits (all results (up to 1000) for each mass)

Elements Used:

C: 0-100 H: 0-200 O: 0-1 Na: 0-1 Si: 2-2

Mass	Calc. Mass	mDa	PPM	DBE	Formula	i-FIT	i-FIT Norm	Fit Conf %	C	H	O	Na	Si
413.1740	413.1733	0.7	1.7	11.5	C24 H30 O Na Si2	755.1	0.044	95.65	24	30	1	1	2
	413.1757	-1.7	-4.1	14.5	C26 H29 O Si2	758.2	3.136	4.35	26	29	1		2
